# Redox‐Active Guanidines in Proton‐Coupled Electron‐Transfer Reactions: Real Alternatives to Benzoquinones?

**DOI:** 10.1002/chem.201903438

**Published:** 2019-10-23

**Authors:** Ute Wild, Olaf Hübner, Hans‐Jörg Himmel

**Affiliations:** ^1^ Anorganisch-Chemisches Institut Ruprecht-Karls-Universität Heidelberg Im Neuenheimer Feld 270 69120 Heidelberg Germany

**Keywords:** guanidine, oxidation, oxidative coupling, proton-coupled electron transfer, redox reaction

## Abstract

Guanidino‐functionalized aromatics (GFAs) are readily available, stable organic redox‐active compounds. In this work we apply one particular GFA compound, 1,2,4,5‐tetrakis(tetramethylguanidino)benzene, in its oxidized form in a variety of oxidation/oxidative coupling reactions to demonstrate the scope of its proton‐coupled electron transfer (PCET) reactivity. Addition of an excess of acid boosts its oxidation power, enabling the oxidative coupling of substrates with redox potentials of at least +0.77 V vs. Fc^+^/Fc. The green recyclability by catalytic re‐oxidation with dioxygen is also shown. Finally, a direct comparison indicates that GFAs are real alternatives to toxic halo‐ or cyano‐substituted benzoquinones.

## Graphical abstract

**Acid addition** boosts the oxidative power of redox‐active guanidines in their oxidized state, making them a real alternative to toxic benzoquinone derivatives for oxidation/oxidative coupling reactions (see figure).

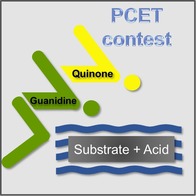

Proton‐coupled electron transfer (PCET) is important for biological and bioinspired (photosynthetic) processes as well as synthetic chemistry,^1^, ^2^, ^3^ and has been studied intensively mechanistically.^4^, ^5^ Quinones are especially versatile organic PCET reagents. Their redox‐properties and the p*K*_a_ values of their corresponding hydroquinones can be varied by the introduction of substituents,^6^, ^7^, ^8^ and also by electronic excitation.^9^, ^10^ Figure 1 shows as examples the three benzoquinones BQ, CA and DDQ. The 1 e^−^ redox potentials (*E*_red_ vs. Fc^+^/Fc) of DDQ (+0.14 V) and CA (−0.35 V) are significantly higher than that of BQ (−0.88 V).^8^ However, the reduction of the p*K*_a_ value of the reduced form that accompanies the increase of *E*_red_ of the oxidized form leads to a certain “leveling” effect on the PCET reactivity.^7^, ^8^ Benzoquinones with a relatively high oxidation potential, for example, CA and DDQ, are used in a number of PCET reactions as stoichiometric oxidation reagents, often in combination with a strong acid,^11^, ^12^, ^13^ and in some reactions also catalytically, for example, DDQ together with nitrite.^14^ Low‐potential *p*‐benzoquinone derivatives were used as redox‐mediators in biomimetic catalysis and as redox catalysts (often together with transition metal complexes).^15^, ^16^, ^17^ Moreover, low‐potential *o*‐quinone‐type catalysts were recently shown to enable manifold (bioinspired) aerobic oxidations.^18^, ^19^, ^20^, ^21^, ^22^, ^23^, ^24^, ^25^, ^26^

**Figure 1:**
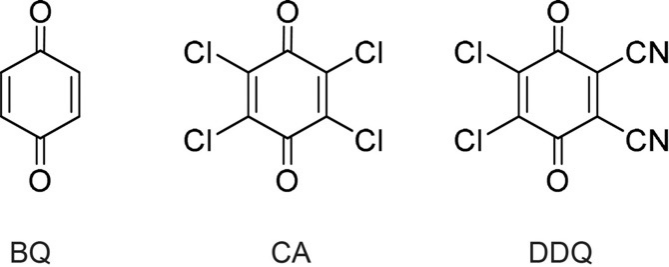
Lewis structures of *p*‐benzoquinone (BQ), chloranil (CA), and 2,3‐dichloro‐5,6‐dicyano‐benzoquinone (DDQ).

Despite of the outstanding success story of quinones, some drawbacks oppose their large‐scale applications. Hence cyano‐ or halo‐substituted benzoquinones like **CA** are highly toxic, as they induce reactive oxygen species and oxidative stress, showing an inflammatory response both in vivo and in vitro.^27^ Moreover, the recycling of the quinones is sometimes problematic due to side reactions.^11^ Strong oxidizing reagents are required for high‐potential quinones. For low‐potential quinones catalytic oxidation of the hydroquinone with dioxygen is possible,^28^ but could be hampered by the formation of quinhydrones (the 1:1 complex between benzoquinone and hydroquinone exhibits a binding energy of more than 20 kJ mol^−1^)^29^ at high concentrations.^28^

We recently developed a new class of PCET reagents, namely redox‐active guanidines, that do not have these disadvantages.^30^, ^31^, ^32^, ^33^ One example is 1,2,4,5‐tetrakis‐(tetramethylguanidino)benzene (**1**), which could be readily oxidized to the dication **1** ^2+^ (Scheme 1). The loss of aromaticity upon oxidation leads to significantly different C−C bond distances in the C_6_ ring and a distinct colour change from pale yellow for neutral **1** to intense green for the dication **1** ^2+^. Proton‐coupled electron transfer (PCET) reactions of oxidized 1,2,4,5‐tetrakis(tetramethylguanidino)benzene (**1** ^2+^, Scheme 1)^34^ with some inorganic (thiol to disulfides, phosphines to diphosphines)^31^ and organic substrates with relatively low redox‐potential (e.g., phenols to biphenols, catechols to benzoquinones) were already reported.^31^, ^32^ The π‐system of **1** ^2+^ accepts the electrons and the nitrogen lone pairs accept the protons. Using a copper co‐catalyst, **1** ^2+^ could be used as an organocatalyst with dioxygen as the terminal oxidant.^32^ The p*K*_a_ value of ca. 25.3 for (**1**+H)^+^ in CH_3_CN sharply decreases upon oxidation. Interestingly, green **1** ^2+^ still is a Lewis^35^ and Brønsted base, and is protonated with HBF_4_ **⋅**Et_2_O to blue (**1**+H)^3+^ and orange (**1**+2 H)^4+^ (with a p*K*_a_ value of ca. 13 in CH_3_CN close to CF_3_COOH, Scheme 1).^36^ The reduction potential increases from **1** ^2+^ (*E*_1/2_=−0.73 V vs. Fc^+^/Fc in CH_3_CN) to (**1**+2H)^4+^ by ca. 0.7 V.^36^

**Scheme 1:**
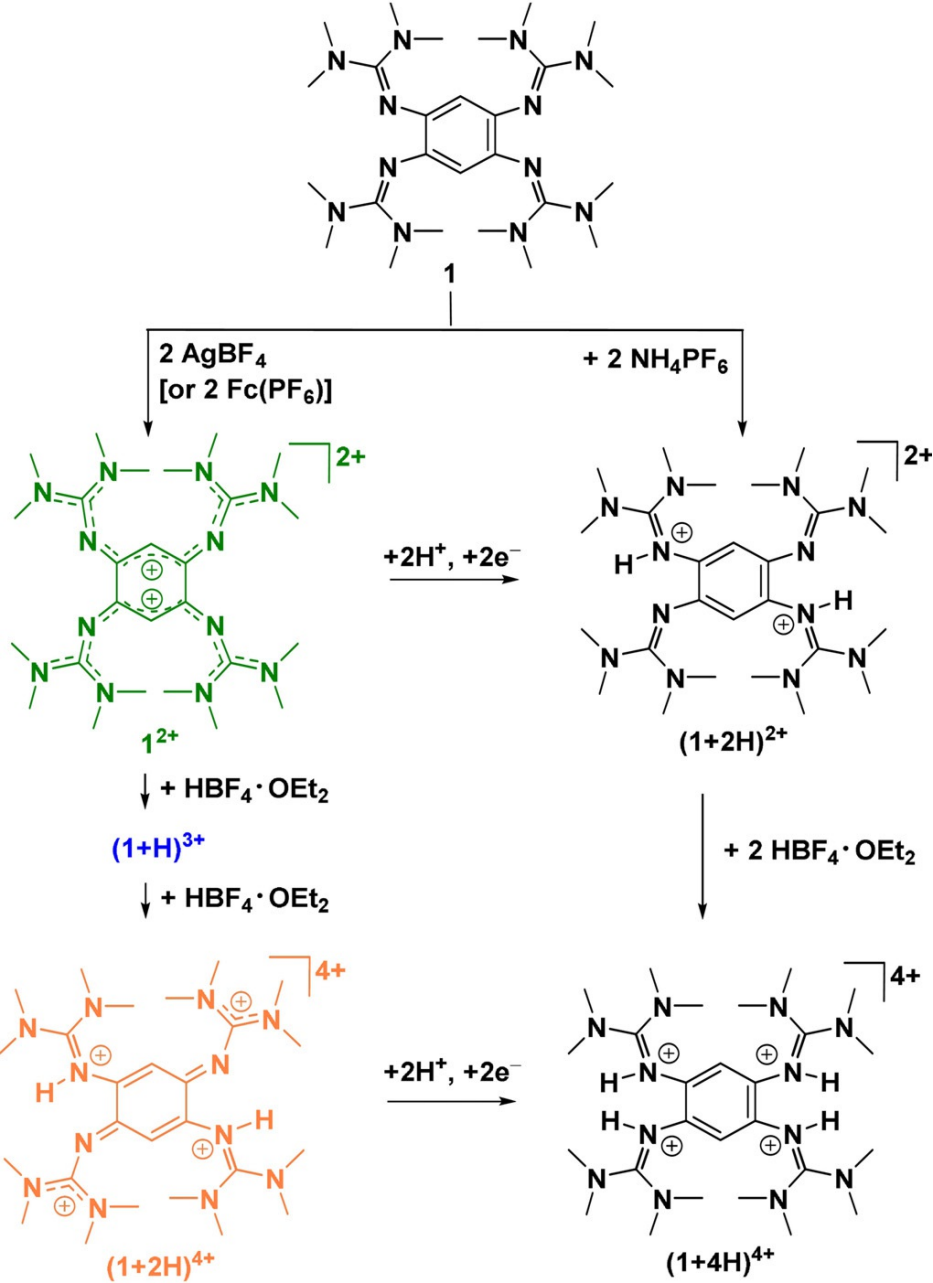
Lewis structures of characterized, stable states, starting with neutral 1,2,4,5‐tetrakis(tetramethylguanidino)‐benzene (**1**), relevant for the PCET reactivity. The colors characteristic for the oxidized states are highlighted.

Herein, we demonstrate the wide scope of its PCET reactivity, especially in combination with strong acids. Nine oxidative coupling/ oxidation reactions were studied with substrates that differ largely in their redox potentials. In addition, we show that efficient regeneration of the PCET reagent **1**(BF_4_)_2_ is possible by catalytic oxidation with dioxygen. Finally, we compare its PCET properties with benzoquinones.

The salts **1**(BF_4_)_2_ (oxidized GFA) and (**1**+2 H)(BF_4_)_4_ (oxidized and protonated GFA)^36^ as well as **1**(BF_4_)_2_ in combination with an excess of the strong acid HBF_4_ **⋅**OEt_2_ were applied. The substrates are grouped in low‐potential [Eqs. (1–4)] and high potential substrates [Eqs. (5–9)], for example, +0.52 V for NPh_3_ (**S_6_**),^37^ +0.66 V for 4,4′‐dibromo‐triphenylamine (**S_7_**), +0.74 V for 3,3′′,4,4′′‐tetramethoxy‐*o*‐terphenyl (**S_9_**),13b and +0.77 V vs. Fc^+^/Fc for 4‐nitro‐triphenylamine (**S_8_**).^38^ To allow for a direct comparison, all reactions were carried out in CH_3_CN solution. The yields were estimated from NMR signal integration (see the Supporting Information).

Oxidative coupling of 2,6‐di‐*tert*‐butyl‐phenol (**S_1_**) to the diketone (**P_1_**) gives best results (82 % yield) with **1**(BF_4_)_2_ [Eq. (1)] rather than (**1**+2 H)(BF_4_)_4_. Oxidation of 3,5‐di‐*tert*‐butyl‐catechol (**S_2_**) to the *o*‐ benzoquinone (**P_2_**) is fast with **1**(BF_4_)_2_, but gives slightly better yields with (**1**+2 H)(BF_4_)_4_ [Eq. (2)]. Both reactions are presumably initiated by deprotonation. Catechol deprotonation is favored by the intramolecular O−H⋅⋅⋅O bridge in the resulting monoanion.^7^, ^39^

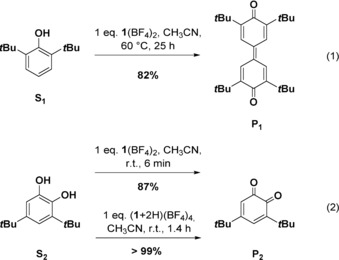



The oxidative coupling of benzylamine (**S_3_**) to give *N*‐(phenylmethylene)benzenemethanamine (**P_3_**) and the oxidation of *o*‐phenylene‐diamine (**S_4_**) to give 2,3‐diaminophenazine (**P_4_**) give best results with (**1**+2 H)(BF_4_)_4_ (79 % respectively 96 % yield, see Eqs. (3) and (4)]. With **1**(BF_4_)_2_, these reactions are much slower, giving less than 10 % yield after 25 h at 60 °C (see the Supporting Information for details). UV/Vis and ^1^H NMR spectra (see the Supporting Information) indicate that (**1**+2 H)^4+^ first protonates the amine, in line with the acidity of (**1**+2 H)^4+^.^36^

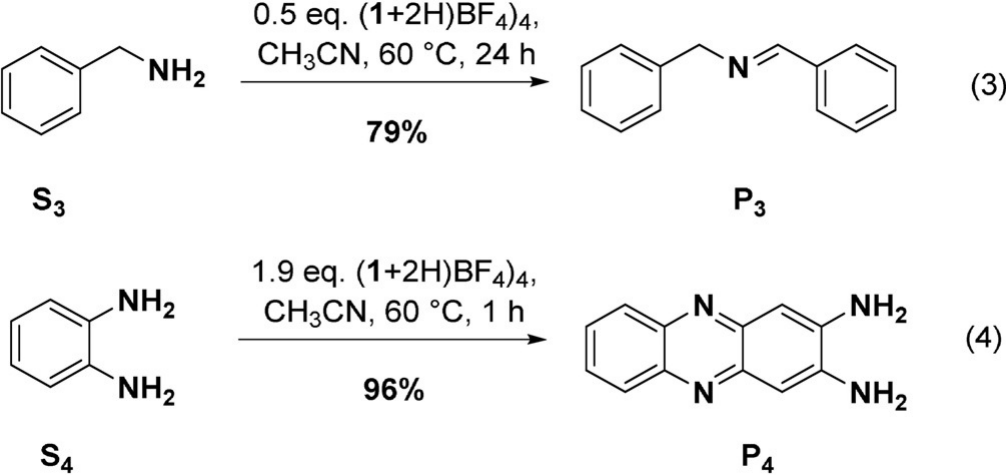



When benzylamine oxidation was repeated with 0.5 equivalents of **1**(BF_4_)_2_ and slightly more than 1 equivalent of NH_4_PF_6_, the reaction proceeded with a similar rate and slightly better yield (84 %).

Next we inspected the reactivity toward substrates with higher redox potentials, requiring the addition of excess acid. Happily, oxidative coupling of triphenylamine and derivatives with electron‐withdrawing and ‐donating groups [**S_5_**–**S_8_**, Eq. (5)–(8)] is accomplished in less than 2.3 h with excellent yields with a combination of **1**(BF_4_)_2_ (equimolar amount) and excess HBF_4_⋅OEt_2_ (see the Supporting Information). The results demonstrate the superior functional‐group tolerance of such coupling reactions. UV/Vis experiments showed the presence of reaction intermediates, arising from substrate oxidation (see the Supporting Information).12h, 40, 41 In principle, the triphenylamine derivatives could be protonated by the strong acid. However, in all cases fast oxidation was observed, indicating that protonation plays no significant role.
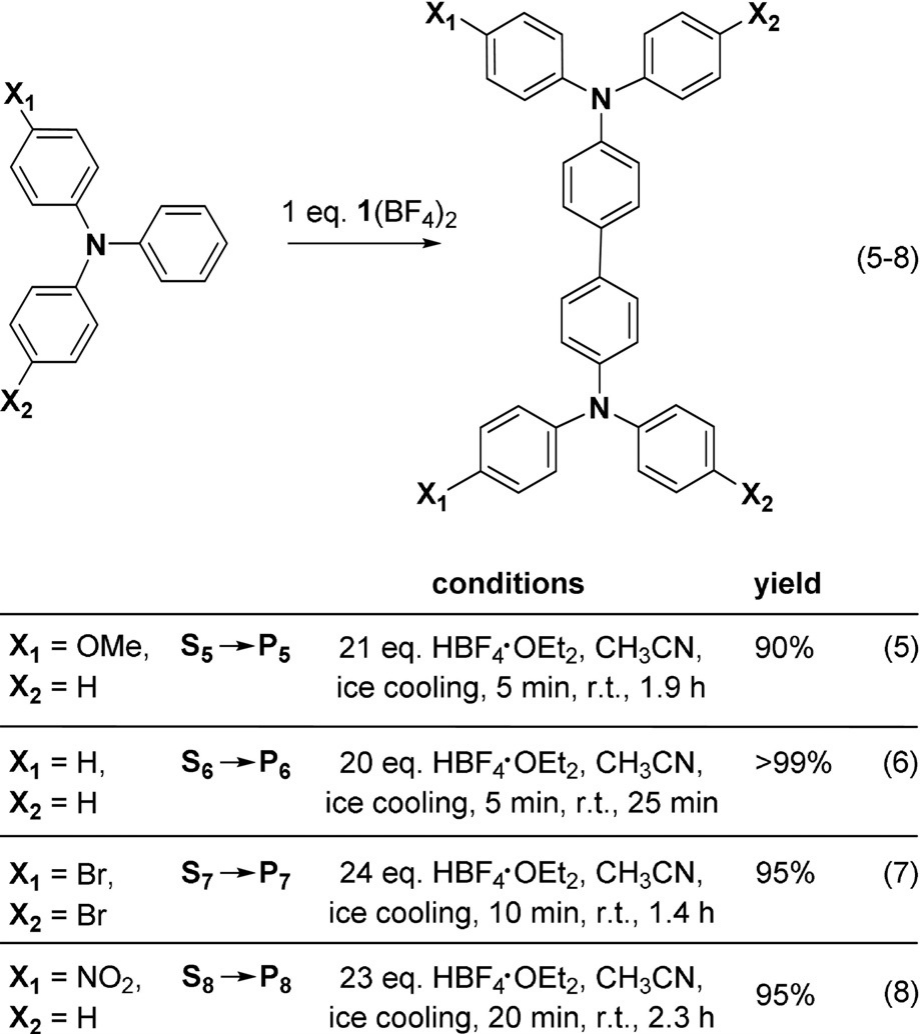



Finally, we tested an intramolecular oxidative coupling reaction of 3,3′′,4,4′′‐tetramethoxy‐*o*‐terphenyl (**S_9_**) [Eq. (9)], a substrate with a high oxidation potential of 0.74 V vs. Fc^+^/Fc. Application of 1 equivalent of **1**(BF_4_) with an excess of HBF_4_⋅OEt_2_ leads to 99 % triphenylene coupling product.
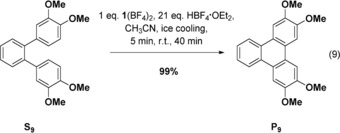



Obviously, (**1**+2 H)(BF_4_)_4_ forms from **1**(BF_4_)_2_ upon acid addition, but the oxidation potential of these organic substrates is still higher than the reduction potential of (**1**+2 H)(BF_4_)_4_.^36^ Hence the addition of excess acid boosts the oxidation power, as also found for benzoquinones in aqueous^42^, ^43^ and aprotic solutions.12b, 12h, 12k, 44, 45

The guanidinium salt could easily be separated from the reaction mixture. We already showed that (**1**+2 H)^2+^ can be quantitatively reconverted to **1** ^2+^ by catalytic oxidation with dioxygen.^32^ In new experiments we tested the recyclability of **1**(BF_4_)_2_ from the reduced tetraprotonated compound (**1**+4 H)(BF_4_)_4_, that is formed in the experiments with (**1**+2 H)(BF_4_)_4_ or an excess of acid. Indeed, quantitative formation of **1**(BF_4_)_2_ (NMR studies, see the Supporting Information) within 30 min at 60 °C was achieved by catalytic oxidation with dioxygen in the presence of 2 equivalents of NEt_3_ (Scheme 2) with a simple, commercially available catalyst (3 mol % of a 1:1 mixture of CuCl_2_/[Cu(H_2_O)_6_](BF_4_)_2_), independent of the concentration of (**1**+4 H)(BF_4_)_4_ (62, 17, and 8 mmol L^−1^). A complex formation between the product (**1** ^2+^) and the reactant [(**1**+2 H)^2+^ or even (**1**+4 H)^4+^], as observed in the case of benzoquinone (quinhydrone complex), is prohibited by strong electrostatic repulsion.

**Scheme 2:**
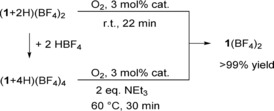
Regeneration of **1**(BF_4_)_2_ from the reduced and two‐ or fourfold protonated forms (cat.=[CuCl_2_/Cu(H_2_O)_6_(BF_4_)_2_]).

The reaction between dihydro‐benzoquinone and **1** ^2+^ in CH_3_CN leads quantitatively in 35 min at r.t. to BQ,^31^ showing that **1** ^2+^ is a stronger PCET reagent than BQ. To gain more information, we calculated the energetics of the reactions in Table 1 by using the B3LYP functional in combination with a def2‐SV(P) or def2‐TZVP basis set. The solvent effect was estimated with the conductor‐like screening model (COSMO) at a relative permittivity *ϵ*_r_ of 40. Calculations with and without BF_4_^−^ counter‐ions gave similar results (see the Supporting Information); we here present results with BF_4_^−^. According to these calculations, **1**(BF_4_)_2_ is similar to BQ with respect to the thermodynamics of its PCET reactions, and slightly weaker than CA. On the other hand, (**1**+2 H)(BF_4_)_4_ is a significantly stronger PCET reagent than all three quinones BQ, CA and DDQ.

**Table 1:** Reaction energies, enthalpies (at 0 K) and Gibbs free energies (at 298 K) for the reaction between the benzoquinones BQ, CA or DDQ and **1**(BF_4_)_2_ respectively (**1**+2 H)(BF_4_)_4_ from B3LYP+COSMO/def2‐TZVP calculations at *ϵ*_r_=1 and 40.

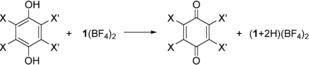						
	X	X′	Δ*E* [kJ mol^−1^]		Δ*H* (0 K) [kJ mol^−1^]	Δ*G* (298 K) [kJ mol^−1^]
			*ϵ*_r_=1	*ϵ*_r_=40	*ϵ*_r_=1	*ϵ*_r_=1
BQ	H	H	−1.8	9.9	1.5	6.0
CA	Cl	Cl	9.9	12.9	12.7	15.2
DDQ	Cl	CN	45.2	59.8	47.3	48.2
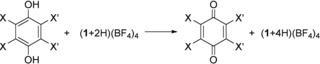						

	X	X′	Δ*E* [kJ mol^−1^]		Δ*H* (0 K) [kJ mol^−1^]	Δ*G* (298 K) [kJ mol^−1^]
			*ϵ*_r_=1	*ϵ*_r_=40	*ϵ*_r_=1	*ϵ*_r_=1
BQ	H	H	−82.3	−67.4	−76.2	−71.0
CA	Cl	Cl	−70.6	−64.3	−65.0	−61.8
DDQ	Cl	CN	−35.3	−17.5	−30.4	−28.8

The effect of hydrogen‐bonding and protonation on the redox‐potential of quinones in aqueous and aprotic solutions^41^, ^46^ was already studied. Moreover, estimates for the p*K*_a_ value of protonated BQ were reported (e.g., from Pourbaix diagrams).^47^, ^48^ On the other hand, monoprotonation of BQ in significant amounts requires the use of superacidic HF/AsF_5_ and low temperature, since the salt (BQ+H)AsF_6_ decomposes already above −60 °C.^49^ By contrast, (**1**+2 H)(BF_4_)_4_ is a storable compound, being stable in the solid state and in solution at ambient conditions.^36^ Consequently, the double‐proton transfer from (**1**+2 H)(BF_4_)_4_ to BQ to give **1**(BF_4_)_2_ and (BQ+2 H)(BF_4_)_2_ (exhibiting almost symmetric F⋅⋅⋅H−O bonds between cation and anion, with F−H: 1.360 Å and O−H: 1.059 Å) was calculated (B3LYP+COSMO/def2‐TZVP) to be associated with a high positive reaction energy of +251 kJ mol^−1^ at *ϵ*_r_=40. Accordingly, no reaction was observed when (**1**+2 H)(BF_4_)_4_ was dissolved together with BQ in CH_3_CN. Interestingly, (BQ+2 H)(BF_4_)_2_ decomposes in the calculations for *ϵ*_r_=1 by fluoride abstraction from the anion to a complex BQ(HF)_2_(BF_3_)_2_ (see the Supporting Information). Moreover, (CA+2 H)(BF_4_)_2_ defines no minimum structure at both *ϵ*_r_=1 and 40, but converges again to the product of fluoride abstraction, CA(HF)_2_(BF_3_)_2_ (Figure 2). The reaction between (**1**+2 H)(BF_4_)_4_ and CA to give, instead of protonated **CA**, the favoured complex CA(HF)_2_(BF_3_)_2_ exhibits a reaction energy of +317 kJ mol^−1^ at *ϵ*_r_=40.

**Figure 2:**
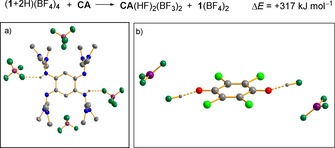
a) Comparison between the experimentally derived structure of the stable compound (**1**+2 H)(BF_4_)_4_ in the solid state (a) and the structure of the CA(HF)_2_(BF_3_)_2_ complex obtained in the attempt to calculate the analogue two‐fold‐protonated CA with two BF_4_^−^ counter‐ions (b).

In summary we demonstrated the preeminent PCET reactivity and efficient recyclability (by green oxidation of (**1**+2 H)(BF_4_)_2_ or (**1**+4 H)(BF_4_)_4_ with dioxygen) of the tetrakisguanidine **1**(BF_4_)_2_. This PCET reagent is readily synthesized (in two steps starting from commercially available 1,2,4,5‐tetraaminobenzene‐tetrahydrochloride), easy to handle, and thermally stable.^30^, ^34^, ^36^, ^50^ The results show that the combination of **1**(BF_4_)_2_ with a strong acid allows the fast and near quantitative oxidative coupling of substrates with high redox potentials (at least +0.77 V vs. Fc^+^/Fc) at mild conditions, making the compound a real alternative to traditionally applied toxic benzoquinone derivatives.

## Conflict of interest

The authors declare no conflict of interest.

## Supporting information

As a service to our authors and readers, this journal provides supporting information supplied by the authors. Such materials are peer reviewed and may be re‐organized for online delivery, but are not copy‐edited or typeset. Technical support issues arising from supporting information (other than missing files) should be addressed to the authors.

Supplementary
