# Supplementary material for: Redox‐Active Guanidines in Proton‐Coupled Electron‐Transfer Reactions: Real Alternatives to Benzoquinones?
Source: Chemistry. 2019 Oct 23;25(70):15988–92. doi: 10.1002/chem.201903438 (PMC7065378; doi:10.1002/chem.201903438)
Supplement: Supplementary file 1 — Supplementary [file CHEM-25-15988-s001.pdf]

CHEMISTRY
A European Journal

Supporting Information

Redox-Active Guanidines in Proton-Coupled Electron-Transfer Reactions: Real Alternatives to Benzoquinones?

Ute Wild, Olaf Hübner, and Hans-Jörg Himmel*[a]

chem_201903438_sm_miscellaneous_information.pdf

Content

No.	Title	Page
1	Experimental Details for oxidative coupling of 2,6-di-*tert*-butyl-phenol to 3,3',5,5'-tetra-*tert*-butyldiphenoquinone	3
2	Experimental Details for oxidation of 3,5-di-*tert*-butylcatechol to the di-*tert*-butyl-*o*-benzoquinone	7
3	Experimental Details for oxidative coupling of benzylamine to *N*-(phenylmethylene)-benzenemethanamine	10
4	Experimental Details for oxidative coupling of *o*-phenylene-diamine to 2,3-diaminophenazine	15
5	Experimental Details for oxidative coupling of 4-methoxytriphenylamine to *N,N'*-bis-(4-methoxyphenyl)-*N,N'*-diphenylbenzidine	19
6	Experimental Details for oxidative coupling of triphenylamine to *N,N,N',N'*-tetraphenylbenzidine	23
7	Experimental Details for oxidative coupling of 4,4'-dibromotriphenylamine to *N,N,N',N'*-tetrakis(4-bromophenyl)-benzidine	32
8	Experimental Details for oxidative coupling of 4-nitrotriphenylamine to *N,N'*-bis-(4-nitrophenyl)-*N,N'*-diphenylbenzidine	36
9	UV-Vis data	40
10	Experimental Details for oxidative coupling of 3,3'',4,4''-tetramethoxy-*o*-terphenyl to 2,3,10,11-tetramethoxy-triphenylene	41
11	Recycling Experiments	45
12	Quantum-chemical calculations	48
13	Acknowledgements	98

General information

All synthetic work was carried out using standard Schlenk techniques. Solvents were dried with an MBraun Solvent Purification System directly prior to use. The following chemicals were purchased and used as delivered: hexamethylbenzene (99%, abcr), 2,6-di-*tert*-butylphenol (99%, Sigma Aldrich), 3,5-di-*tert*-butylcatechol (99%, Acros Organics), benzylamine (99%, Sigma Aldrich), *o*-phenylendiamine (≥98%, Fluka), 4-methoxytriphenylamine (97%, Sigma Aldrich), triphenylamine (98%, Sigma Aldrich), 4,4'-dibromotriphenylamine (98%, Alfa Aesar), 4-nitrotriphenylamine (98%, abcr). The synthesis of 3,3'',4,4''-tetramethoxy-*o*-terphenyl,[1] **1**$(BF_4)_2$,[2] and **1**$(PF_6)_2$[3] followed the literature procedures. Elemental analyses were carried out at the Microanalytical Laboratory of the University of Heidelberg. NMR spectra were recorded on a Bruker DPX 200, Bruker DRX 200, Bruker Avance II 400 or Bruker AVANCE III 600 system. Solvent resonances were taken as references for all ^{1}H NMR spectra. UV-Vis spectra were recorded with a Cary 5000 spectrophotometer and cyclic voltammetry (CV) measurements with an EG&G Princeton Applied Research Potentiostat / Galvanostat 273A or a Methrom Autolab potentiostat PGSTAT 204. A FT-IR Biorad Merlin Excalibur FT300 spectrometer was used for IR measurements. MS-ESI measurements relied on a Bruker microTOF II ESI mass spectrometer.

[1] Linyi Zhai, Ruchi Shukla, Shirya H. Wadumethrige, Rajendra Rathore, *J. Org. Chem.* **2010**, *75*, 4748–4760.
[2] U. Wild, O. Hübner, L. Greb, M. Enders, E. Kaifer, H.-J. Himmel, *Eur. J. Org. Chem.* **2018**, 5910–5915.
[3] U. Wild, S. Federle, A. Wagner, E. Kaifer, H.-J. Himmel, *Chem. Eur. J.* **2016**, *22*, 11971–11976.

1) Experimental details for oxidative coupling of 2,6-di-*tert*-butylphenol to 3,3',5,5'-tetra-*tert*-butyldiphenoquinone

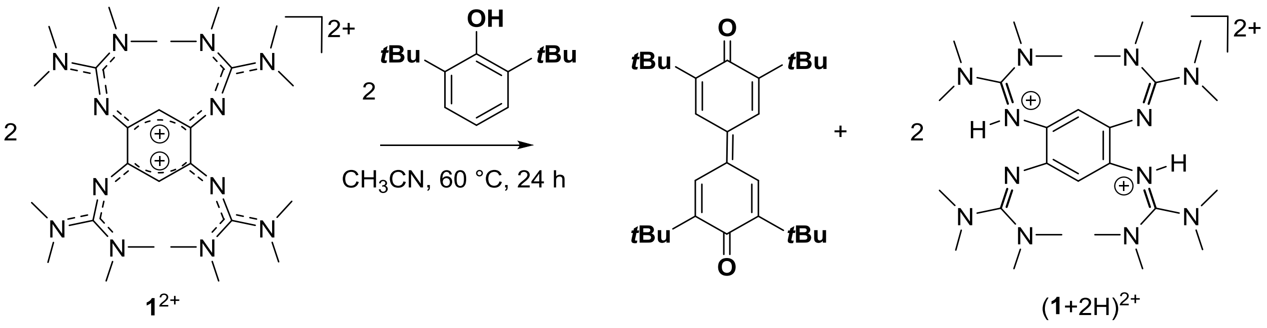

entry	A	B	C	D
**1**$(BF_4)_2$ [mg/µmol]	4.466/6.34	-	5.244/7.44	-
**1**$(PF_6)_2$ [mg/µmol]	-	5.206/6.34	-	5.488/6.69
2,6-di-*tert*-butylphenol [mg/µmol]	1.270/6.16	1.318/6.39	1.514/7.34	1.310/6.34
HMB [mg/µmol]	1.022/6.30	0.800/4.93	1.360/8.38	1.552/9.56
Na_2SO_4 [mg]	-	3.504	-	-
CH_3CN [mL]	0.45	0.45	0.45	0.45
reaction time (temperature)	24 h (60 °C)	24 h (60 °C)	28 h (60 °C)	28 h (60 °C)
conversion phenol to 3,3',5,5'-tetra-*tert*-butyldiphenoquinone (rel. to **1**$^{2+}$/(**1**+2H)$^{2+}$) [%]*	82	86	57	54
ratio (substrate/GFA)	1/1	1/1	1/1	1/1.1

entry	E	F
(**1**+2H)$(BF_4)_4$ [mg/µmol]	3.992/4.54	5.298/6.02
2,6-di-*tert*-butylphenol [mg/µmol]	1.118/5.42	1.226/5.94
HMB [mg/µmol]	0.946/5.83	0.938/5.78
CH_3CN [mL]	0.45	0.45
reaction time (temperature)	25 h (60°C)	24 h (60°C)
conversion phenol to 3,3',5,5'-tetra-*tert*-butyldiphenoquinone (rel. to **1**$^{2+}$/(**1**+2H)$^{2+}$) [%]*)	12	11
ratio (substrate/GFA)	1/0.8	1/1

* The signals of 3,3',5,5'-tetra-*tert*-butyldiphenoquinone could not be used for peak integration, as it precipitates in crystalline form from the reaction mixture.

General protocol (NMR experiments)

In an NMR tube, the reactants **1**(BF_4)$_2$ / **1**(PF_6)$_2$) or (**1**+2H)(BF_4)$_4$, 2,6-di-*tert*-butylphenol were dissolved (with HMB) under an Ar atmosphere in 0.45 mL CD_3CN. The reaction mixture was heated to (60 °C). The conversion was followed by NMR measurements (at room temperature). The overall reaction time refers to the time at (60 °C).

^{1}H NMR spectra (199.87 MHz, 298 K, CD_3CN) for entry A: first measurement

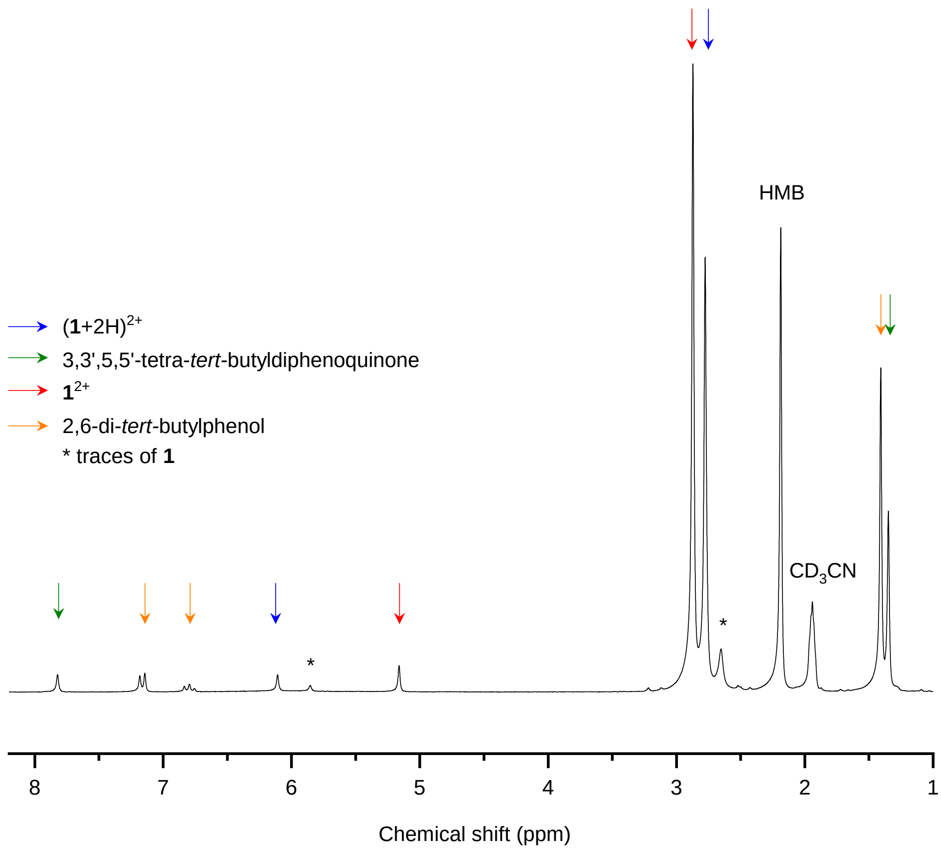

^{1}H NMR spectra (199.87 MHz, 298 K, CD_3CN) for entry A: last measurement

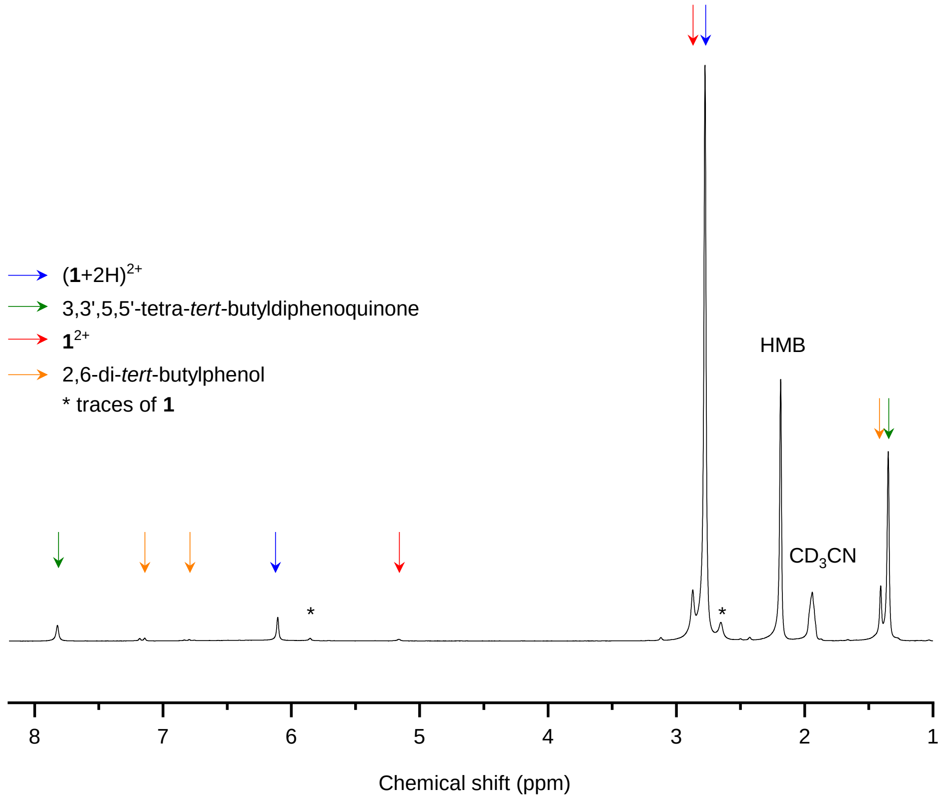

^{1}H NMR 3,3',5,5'-tetra-*tert*-butyldiphenoquinone: (199.87 MHz, 298 K, CD_3CN): δ = 1.35 (s, 36 H), 7.82 (s, 4 H) ppm.

^{1}H NMR spectra (199.87 MHz, 298 K, CD_3CN) for entry C: last measurement

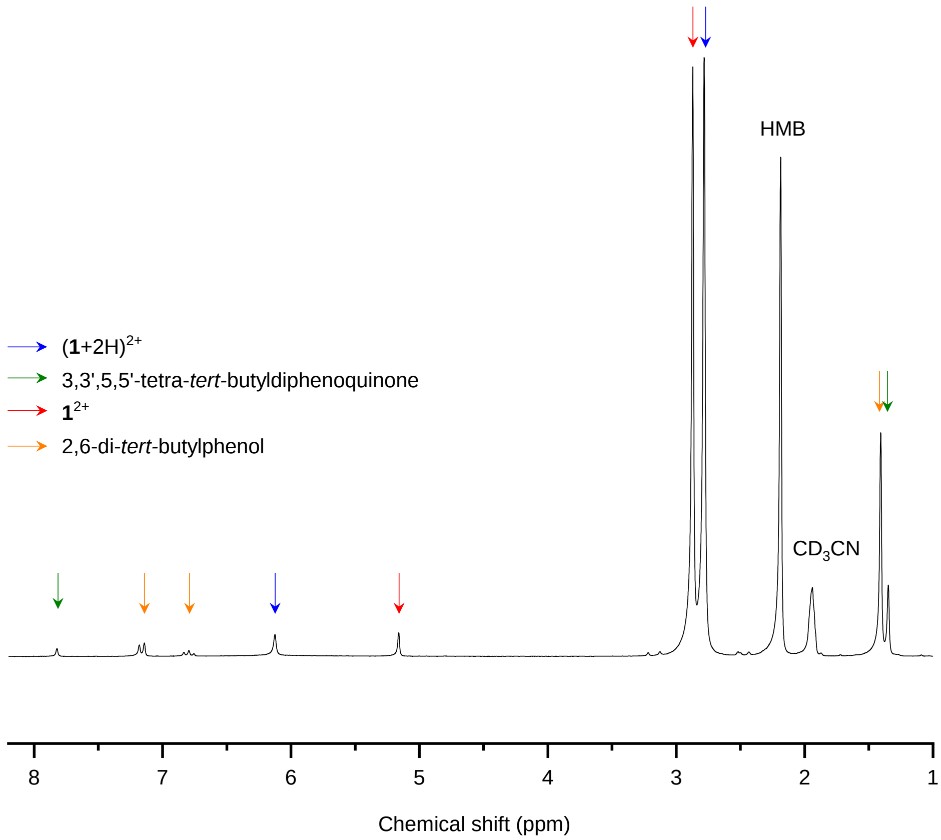

^{1}H NMR spectra (199.87 MHz, 298 K, CD_3CN) for entry E: first measurement

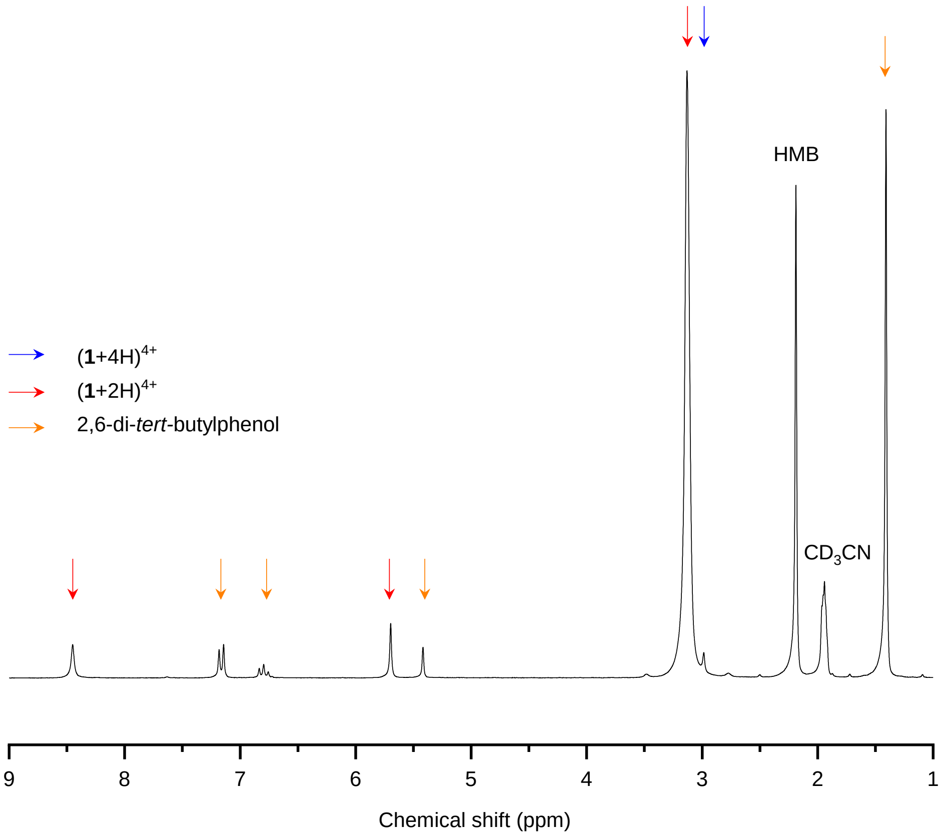

^{1}H NMR spectra (199.87 MHz, 298 K, CD_3CN) for entry E: last measurement

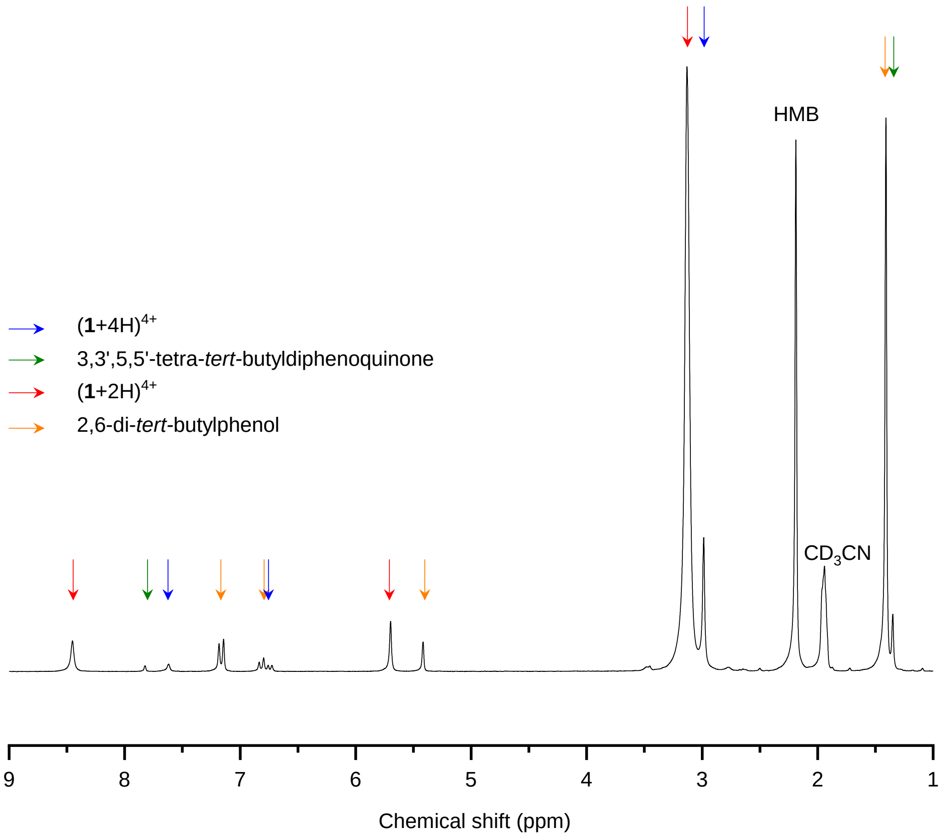

2) Experimental details for oxidation of 3,5-di-*tert*-butylcatechol to di-*tert*-butyl-*o*-benzoquinone

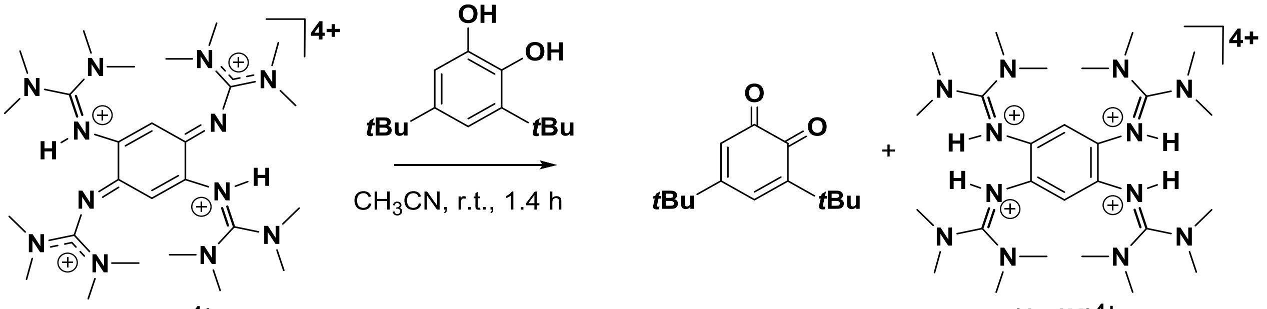

$(\mathbf{1}+2H)^{4+}$ $(\mathbf{1}+4H)^{4+}$

**entry**	**A (373-2)**	**B (334-2)**
**1**$(BF_4)_2$ [mg/µmol]	-	5.286/7.50
(**1**+2H)$(BF_4)_4$ [mg/µmol]	5.882/6.68	-
3,5 di-*tert*-butylcatechol [mg/µmol]	1.460/6.58	1.634/7.35
HMB [mg/µmol]	0.982/6.05	1.188/7.32
CD_3CN [mL]	0.45	0.45
reaction time (temperature)	1.4 h (r.t.)	6 min (r.t.)
conversion (rel. to 3,5 di-*tert*-butylcatechol) [%]	>99	87
conversion (rel. to [$\mathbf{1}+nH^{n+}$]/[$\mathbf{1}^{2+}$])[%]	95	83
ratio (substrate/GFA)	1/1	1/1

General protocol (NMR experiments)

In an NMR tube, the reactants **1**$(BF_4)_2$ or (**1**+2H)$(BF_4)_4$ and 3,5 di-*tert*-butylcatechol were dissolved (with HMB) under an Ar atmosphere in 0.45 mL CD_3CN.

^{1}H NMR spectra (200.13 MHz, 298 K, CD_3CN) for entry A: first measurement

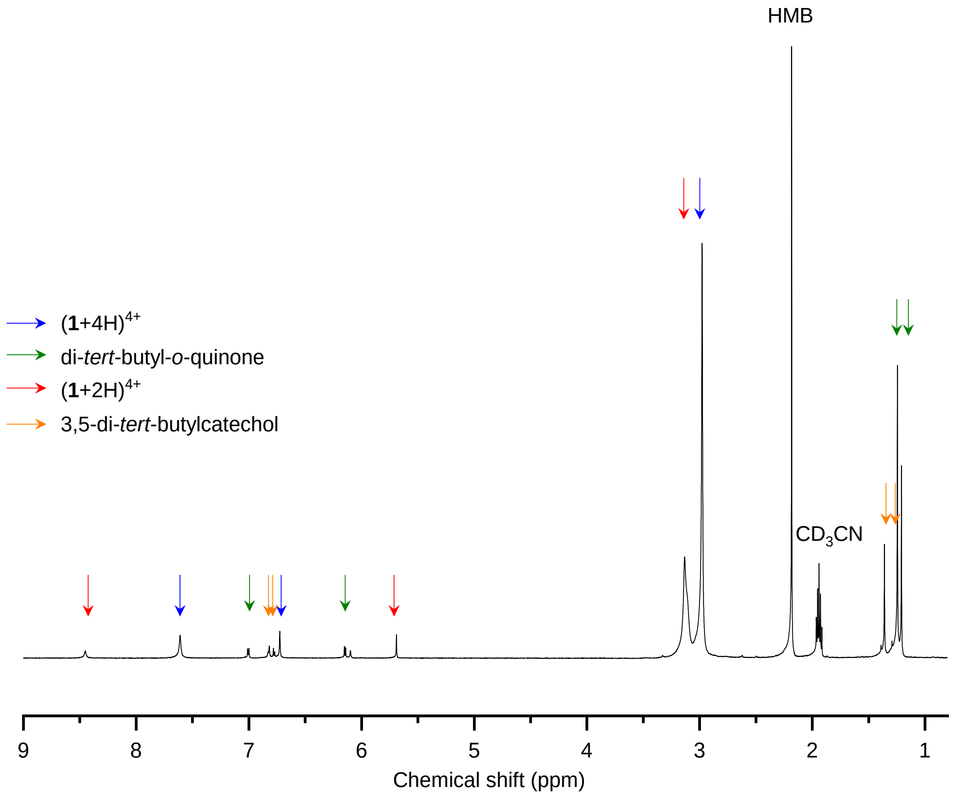

^{1}H NMR spectra (200.13 MHz, 298 K, CD_3CN) for entry A: last measurement

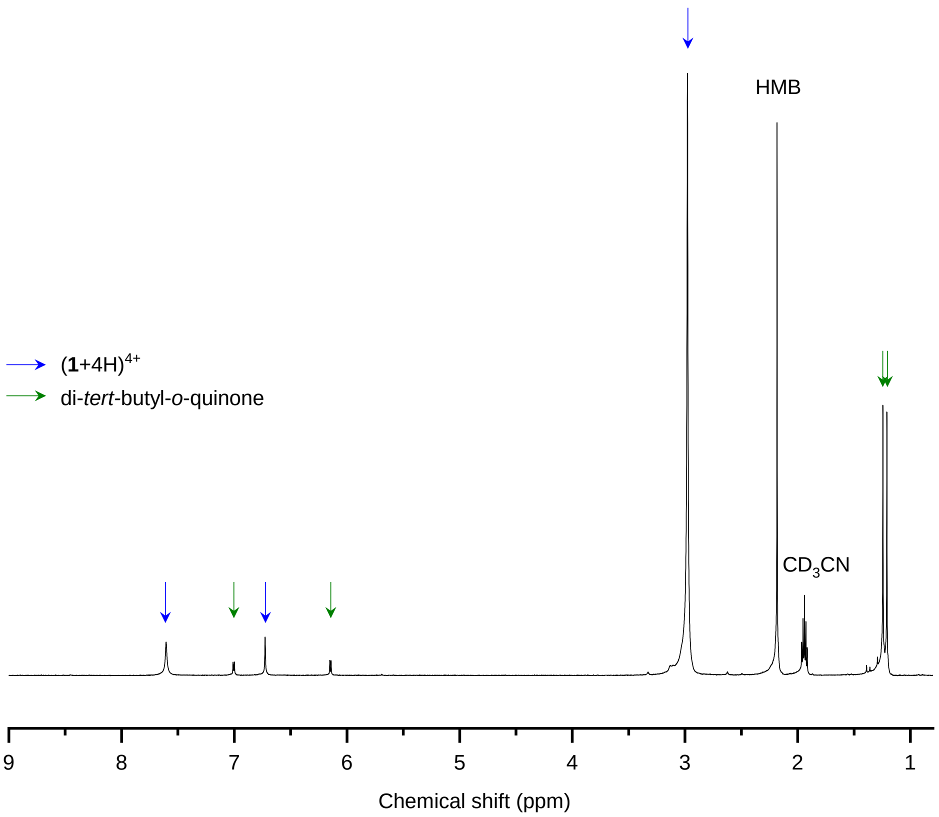

^{1}H NMR of di-*tert*-butyl-*o*-quinone: (200.13 MHz, 298 K, CD_3CN): δ = 1.23 (d, 7.0 Hz, 18 H), 6.15 (d, J = 2.3 Hz, 1 H), 7.01 (d, J = 2.3 Hz, 1 H) ppm.

^{1}H NMR spectra (199.87 MHz, 298 K, CD_3CN) for entry B: last measurement

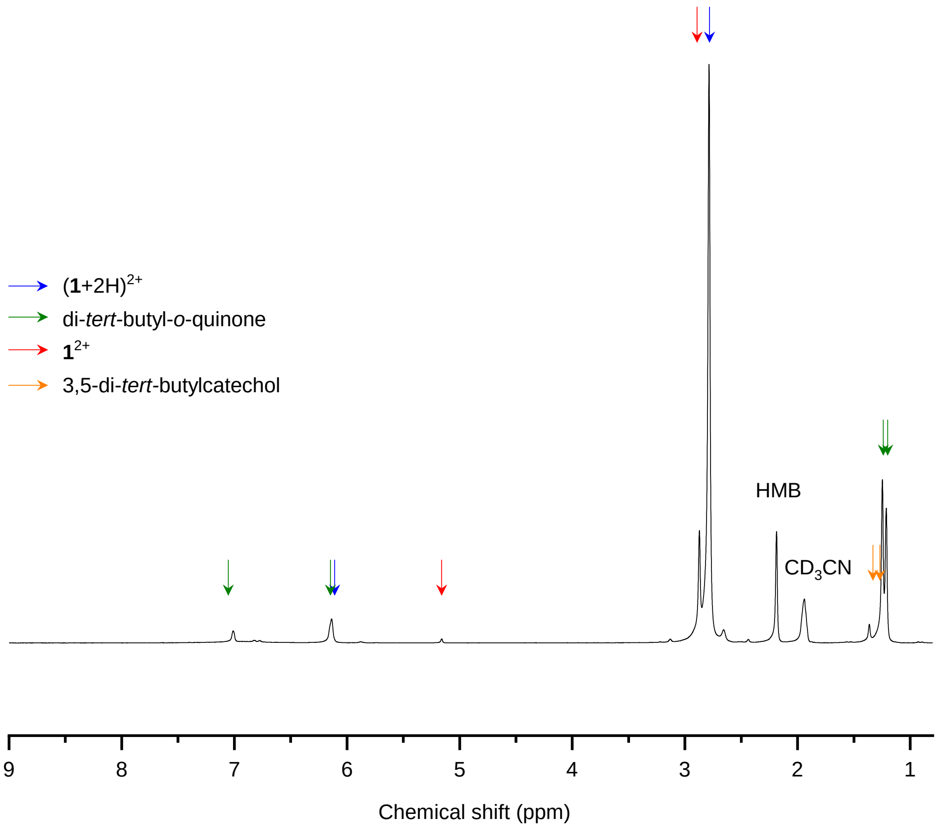

No significant further product formation for prolonged reaction times.

3) Experimental details for oxidative coupling of benzylamine to *N*-(phenylmethylene)-benzenemethanamine

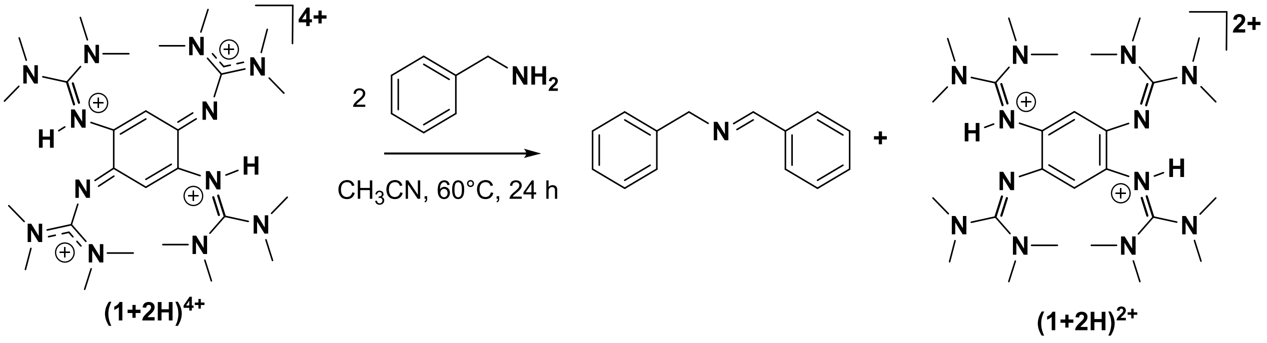

entry	A	B	C	D
**1**$(BF_4)_2$ [mg/µmol]	-	-	5.346/7.59	4.606/6.54
(**1**+2H)$(BF_4)_4$ [mg/µmol]	5.586/6.35	4.87/5.53	-	-
benzylamine (10% in CH_3CN) [µL/mmol]	14/12.80	12/10.97	17/15.54	15/13.71
NH_4PF_6 [mg]	-	-	2.7/16.6	2.3/14.1
HMB [mg/µmol]	0.882/5.44	0.874/5.39	0.920/5.67	1.014/6.25
CH_3CN [mL]	0.45	0.45	0.45	0.47
reaction time (temperature)	24 h (60 °C)	25 h (60 °C)	25.5 h (60 °C)	25 h (60 °C)
conversion (rel. to **1**$^{2+}$/(**1**+2H)$^{2+}$) [%]	79	78	84	79
ratio (substrate/GFA)	1/0.5	1/0.5	1/0.49	1/0.48

entry	E	F
**1**$(BF_4)_2$ [mg/µmol]	5.396/7.66	-
**1**$(PF_6)_2$ [mg/µmol]	-	5.226/6.37
benzylamine (10% in CH_3CN) [µL/mmol]	15/13.71	14/12.80
CH_3CN [mL]	0.45	0.45
HMB [mg/µmol]	0.686/4.23	0.768/4.73
reaction time (temperature)	24 h (60 °C)	24 h (60 °C)
conversion (rel. to **1**$^{2+}$/(**1**+2H)$^{2+}$) [%]	< 5	< 10
ratio (substrate/GFA)	1/0.56	1/0.5

General protocol (NMR experiments)

In an NMR tube, the reactants **1**$(BF_4)_2$ / **1**$(PF_6)_2$) or (**1**+2H)$(BF_4)_4$, and in some experiments also NH_4PF_6 were dissolved (with HMB) in an Ar atmosphere in 0.45 mL CD_3CN. Then a benzylamine solution (10% in CH_3CN) was added dropwise via microliter injection and the reaction mixture heated to 60 °C. The conversion was followed by NMR measurements (at room temperature). The overall reaction time refers to the time at 60 °C.

^{1}H NMR spectra (200.13 MHz, 298 K, CD_3CN) for entry B: first measurement

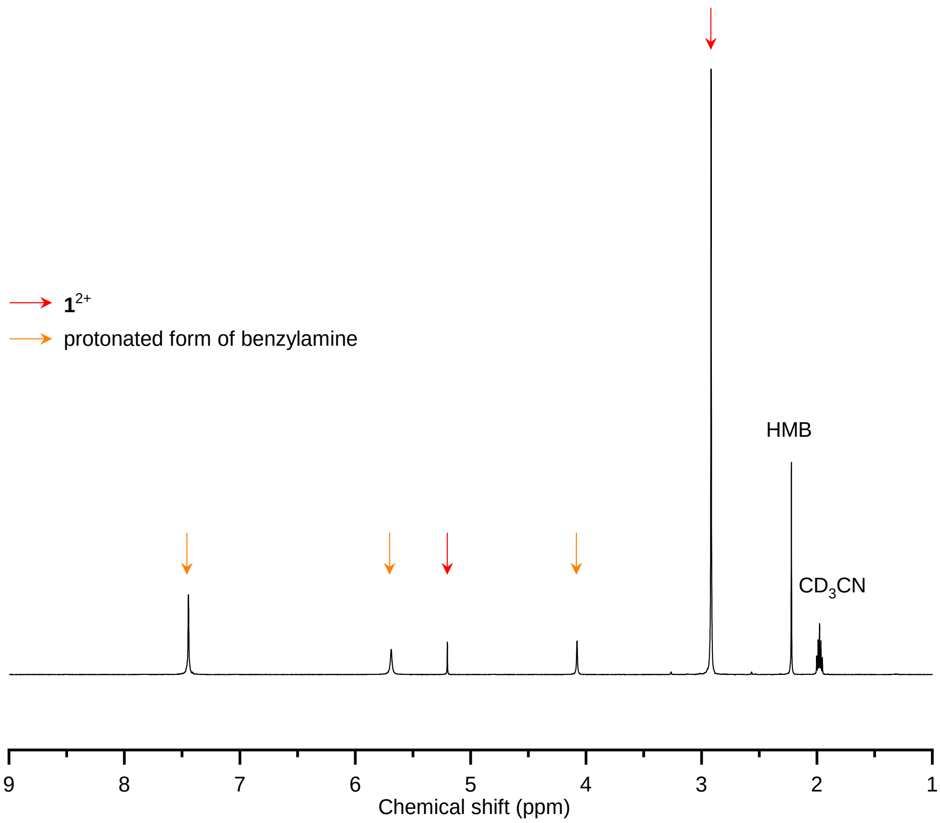

^{1}H NMR spectra (200.13 MHz, 298 K, CD_3CN) for entry B: last measurement

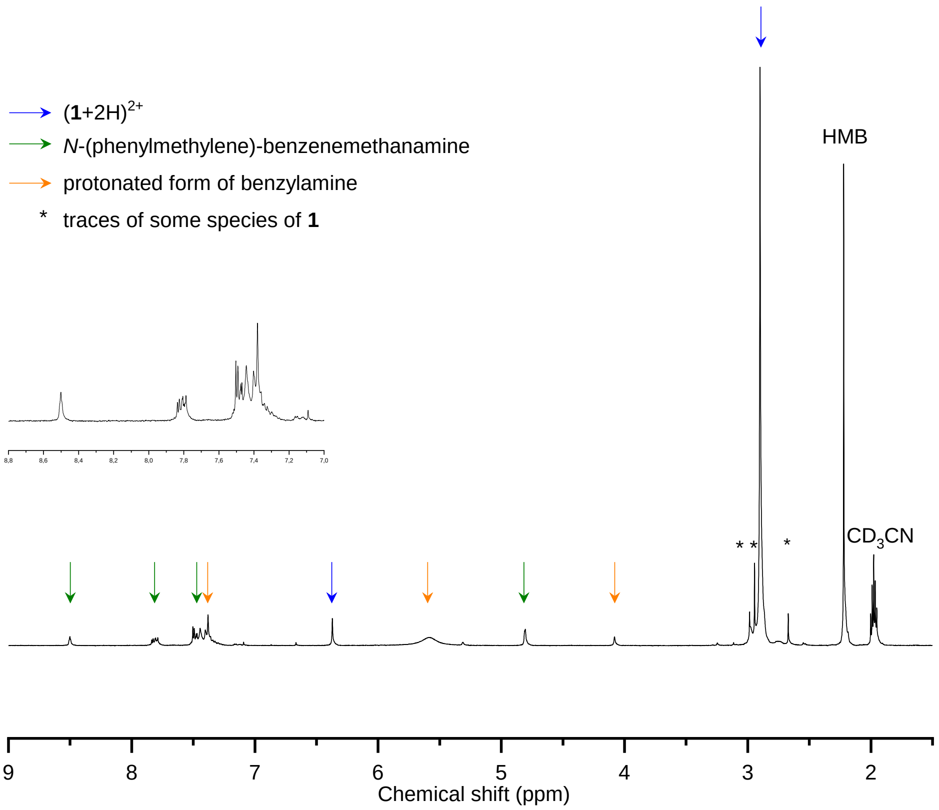

^{1}H NMR of *N*-(phenylmethylene)-benzenemethamine: (200.13 MHz, 298 K, CD_3CN): δ = 4.77 (*d*, *J* = 1.0 Hz, 2 H), 7.44 - 7.34 (m, 8 H), 7.78 (dd, *J* = 6.9; 2.9 Hz, 2 H), 8.47 (s, 1 H) ppm.

^{1}H NMR spectra (200.13 MHz, 298 K, CD_3CN) for entry C: first measurement

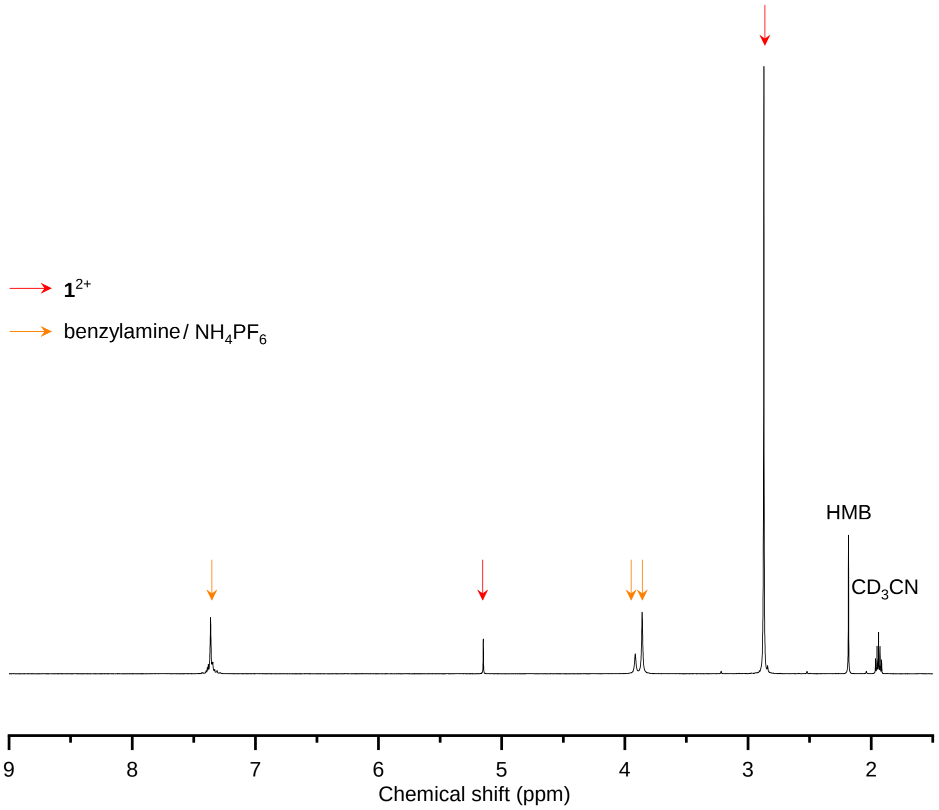

^{1}H NMR spectra (200.13 MHz, 298 K, CD_3CN) for entry C: last measurement

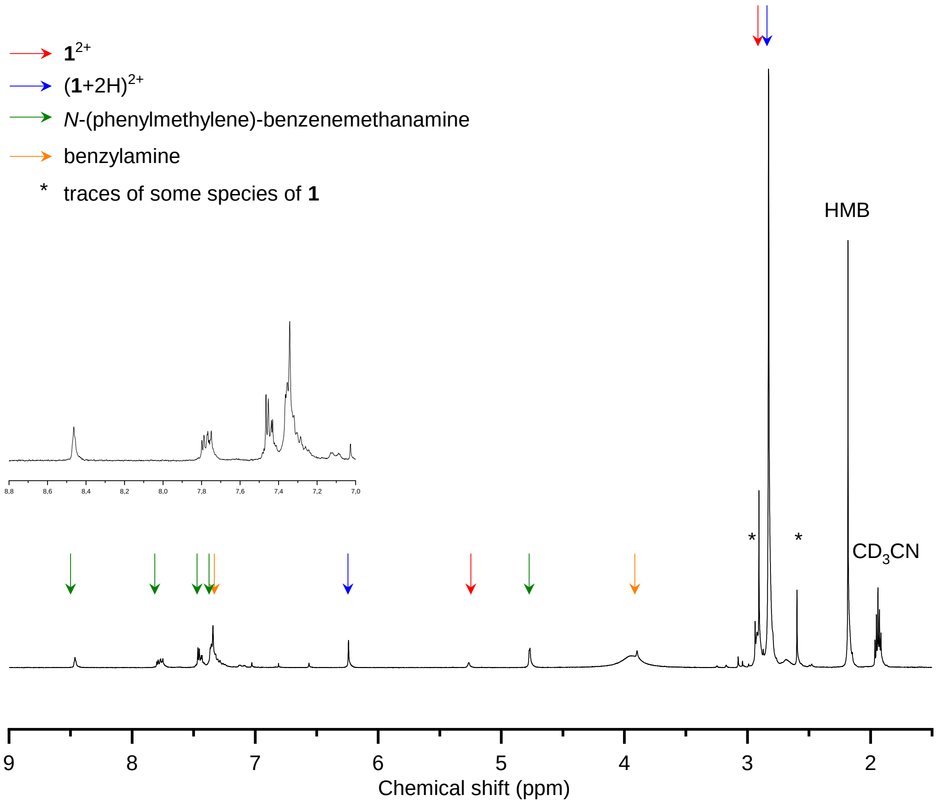

^{1}H NMR spectra (200.13 MHz, 298 K, CD_3CN) for entry F: first measurement

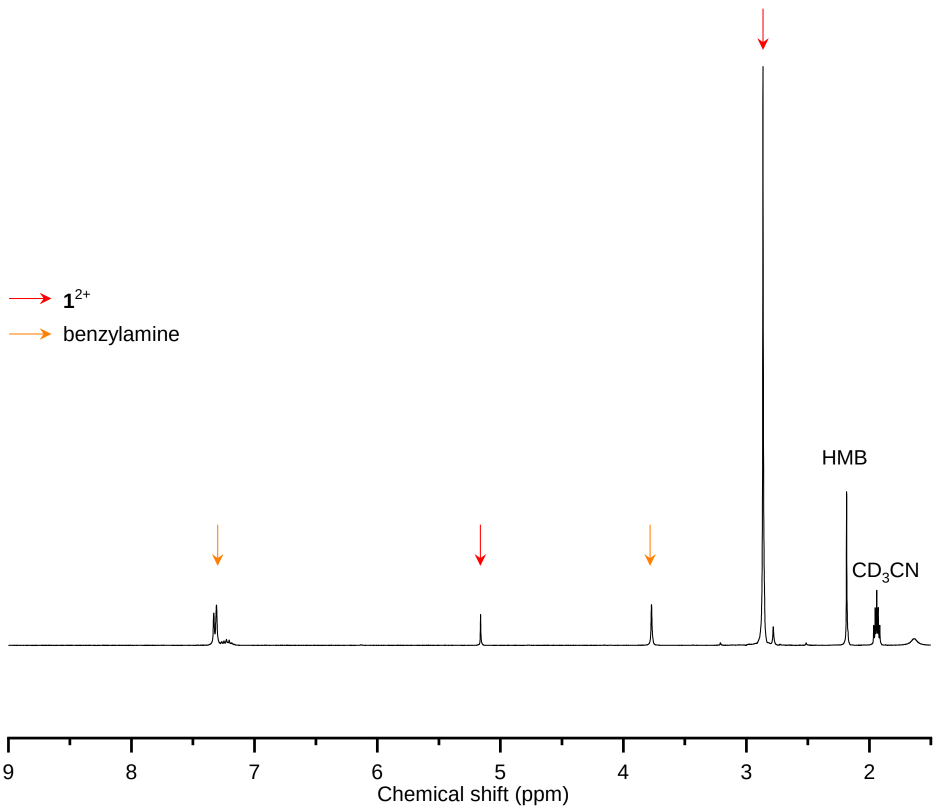

^{1}H NMR spectra (199.87 MHz, 298 K, CD_3CN) for entry F: last measurement

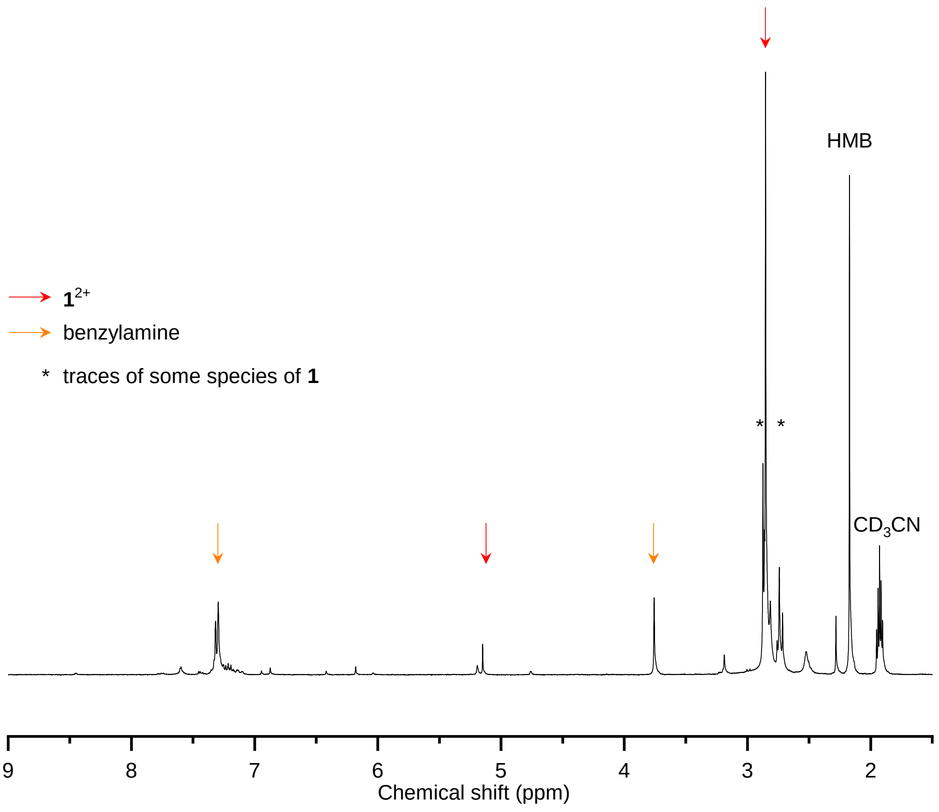

4) Experimental details for oxidative coupling of *o*-phenylene-diamine to 2,3-diaminophenazine

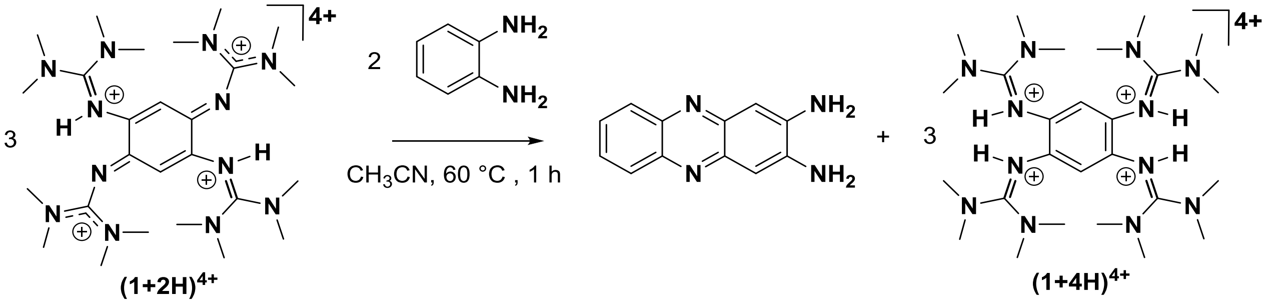

entry	A	B	C
**1**(BF_4)$_2$ [mg/µmol]	-	-	6.696 mg/9.51
(**1**+2H)(BF_4)$_4$ [mg/µmol]	6.059 /6.89	5.036/7.15	-
o-phenylene-diamine	0.506/4.68	0.414/3.83	0.666/6.16
HMB [mg/µmol]	0.736/4.54	1.182/7.28	0.764/4.71
CH_3CN [mL]	0.5	0.45	0.45
reaction time (temperature)	1 h (60 °C)	1 h (60 °C)	25 h (60 °C
conversion (rel. to $\mathbf{1}^{2+}$/($\mathbf{1}$+4H)$^{4+}$) [%]	91	96	<5
ratio substrate/GFA	1/1.5	1/1.9	1/1.5

General protocol (NMR experiments)

In an NMR tube, the reactants **1**(BF_4)$_2$ or (**1**+2H)(BF_4)$_4$ and *o*-phenylene-diamine were dissolved (with HMB) in an Ar atmosphere in 0.45 or 0.5 mL CD_3CN. Then the reaction mixture was heated to 60 °C. The conversion was followed by NMR measurements (at room temperature). The overall reaction time refers to the time at 60 °C.

^{1}H NMR spectra (199.87 MHz, 298 K, CD_3CN) for entry B: first measurement

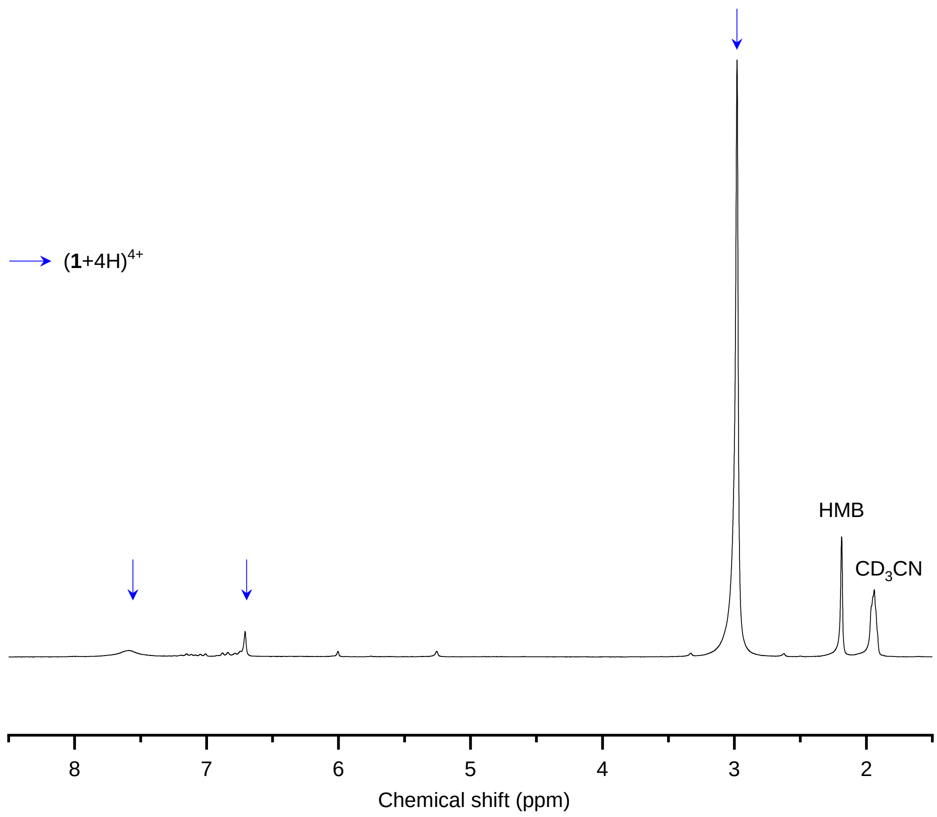

^{1}H NMR spectra (199.87 MHz, 298 K, CD_3CN) for entry A: last measurement

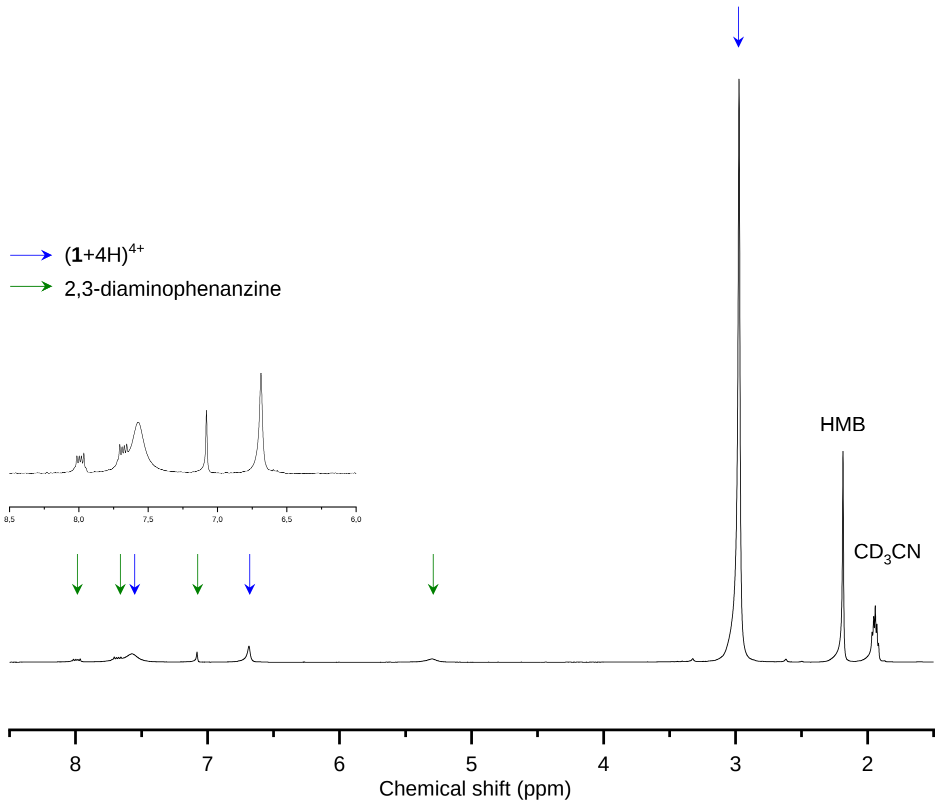

^{1}H NMR of 2,3-diaminophenanzine: (199.87 MHz, 298 K, CD_3CN): δ = 5.29 (bs, 4 H), 7.08 (s, 2 H), 7.68 (dd, J = 6.6; 3.4 Hz, 2 H), 7.99 (dd, J = 6.5; 3.4 Hz, 2 H) ppm.

Reaction was also monitored by UV-Vis spectroscopy in CH_3CN

Reaction with lower concentrations of the reactants (therefore slower) at 60 °C

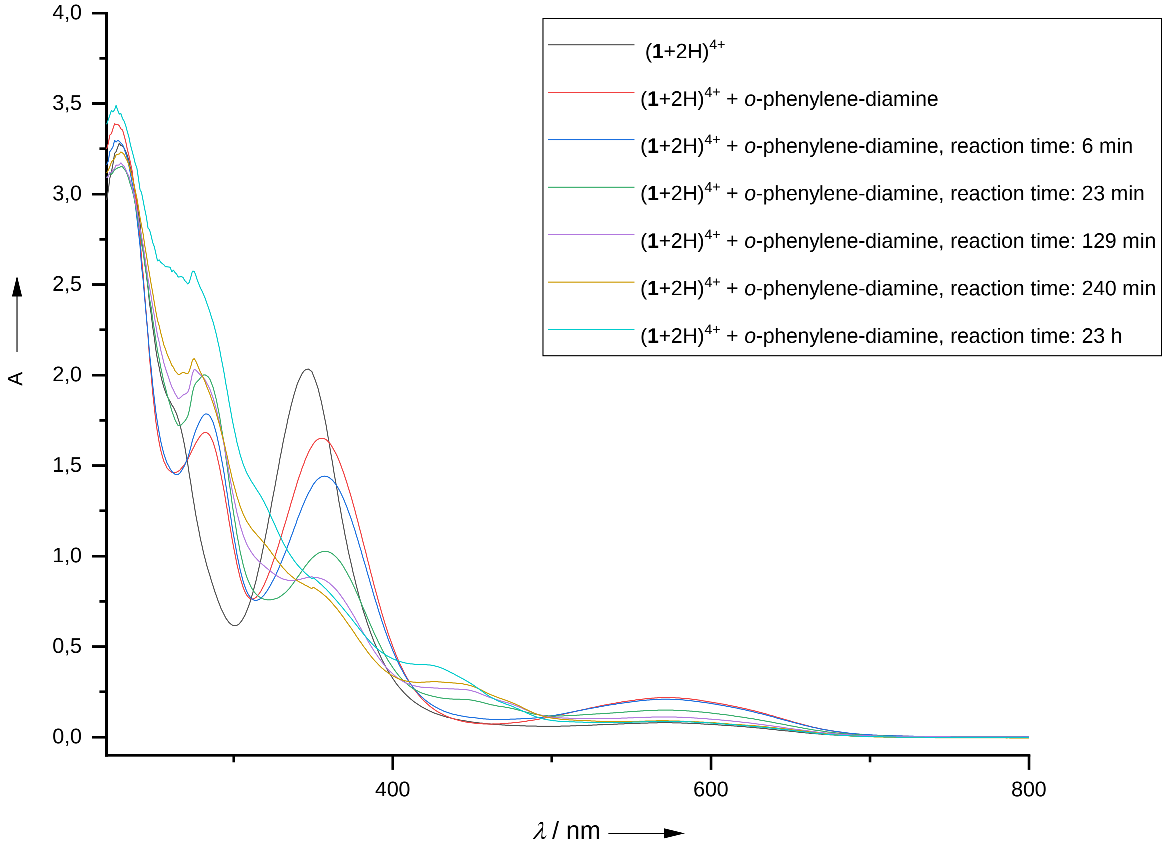

^{1}H NMR spectra (199.87 MHz, 298 K, CD_3CN) for entry C: first measurement

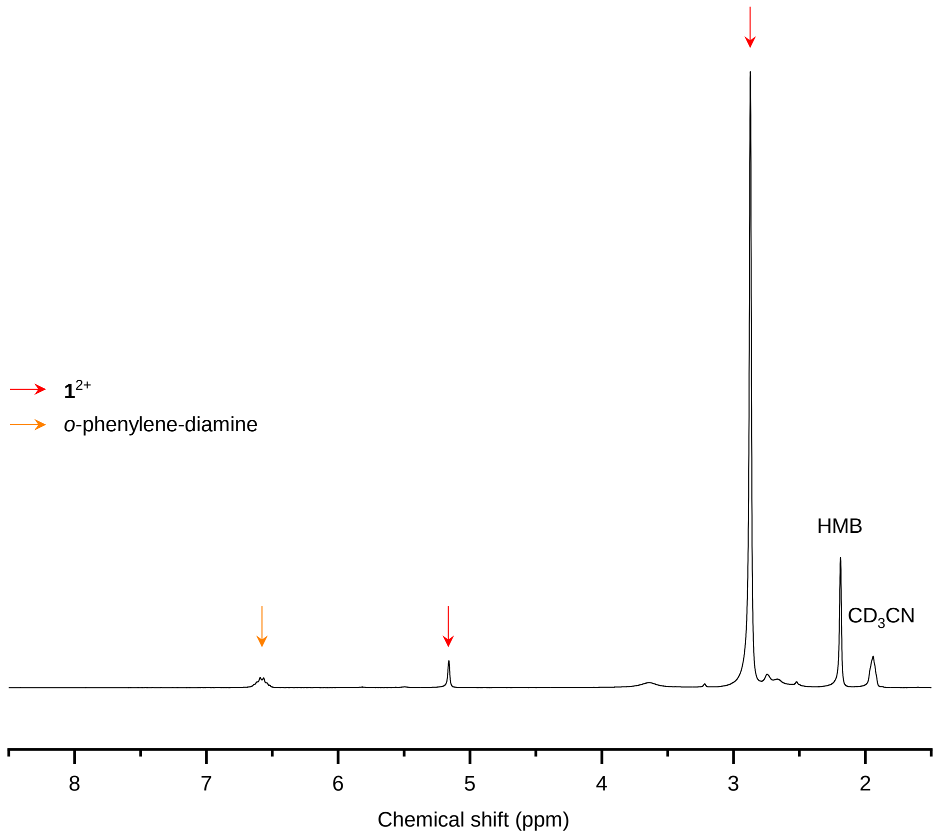

^{1}H NMR spectra (199.87 MHz, 298 K, CD_3CN) for entry C: last measurement

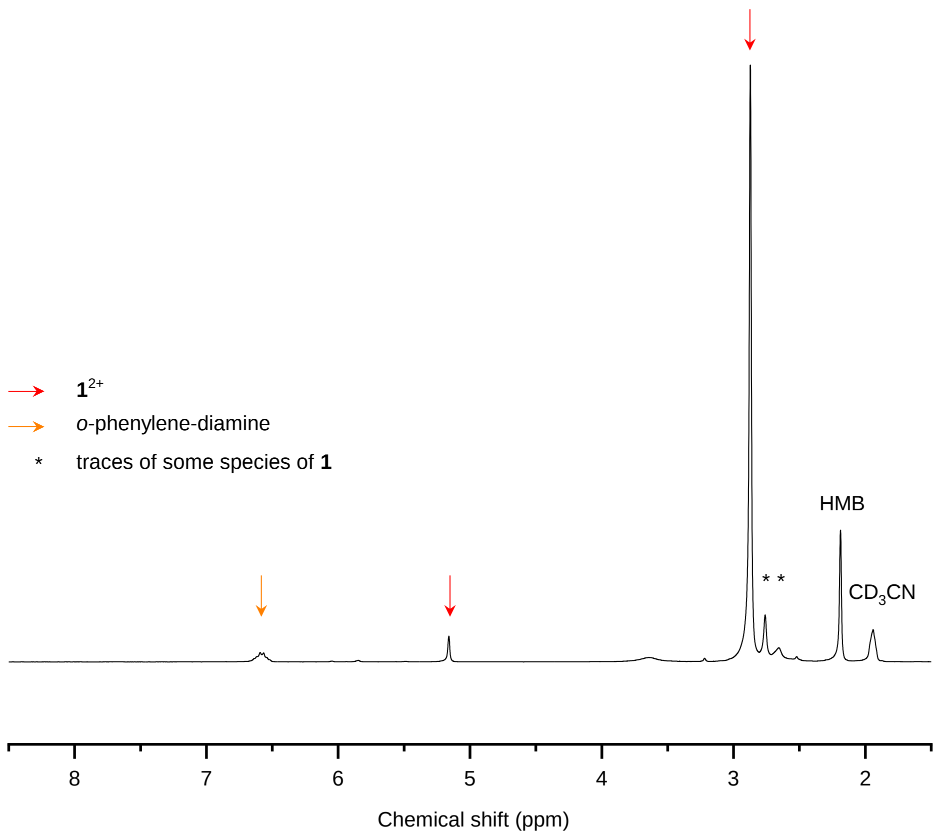

5) Experimental details for oxidative coupling of 4-methoxytriphenylamine to ***N,N'***-bis(4-methoxyphenyl)-***N,N'***-diphenylbenzidine

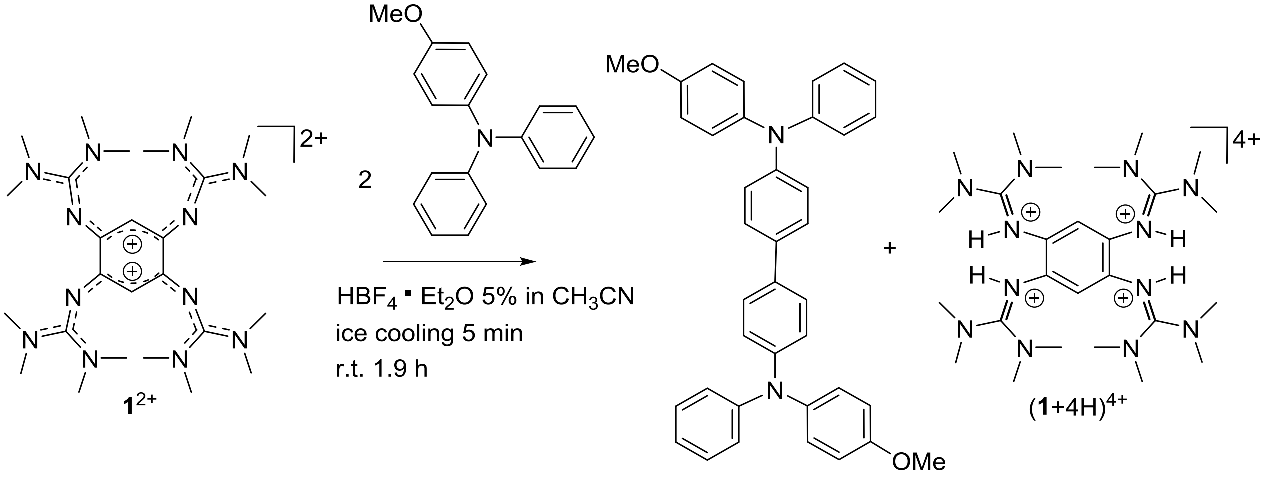

entry	A	B
**1**$(BF_4)_2$ [mg/µmol]	5.992/8.51	-
**1**$(PF_6)_2$ [mg/µmol]	-	6.492/7.91
4-methoxytriphenylamine [mg/mmol]	2.350/8.53	2.196/7.98
$HBF_4 \cdot Et_2O$ (5% in CH_3CN) [mL/µmol]	0.5/182.3	0.5/182.3
HMB [mg/µmol]	1.850/11.40	1.354/8.34
reaction time (temperature)	5 min (ice cooling)<br>1.9 h (r.t.)	5 min (ice cooling)<br>1.3 h (r.t.)
conversion (rel. to $\mathbf{1}^{2+}$/($\mathbf{1}$+2H)$^{2+}$) [%]	90	86
ratio (substrate/GFA)	1/1	1/0.99

General protocol

In a Schlenk flask, 0.5 mL of a $HBF_4 \cdot Et_2O$ solution (5% in CH_3CN) was added under ice cooling in an Ar atmosphere to the reactants **1**$(BF_4)_2$ / **1**$(PF_6)_2$) and 4-methoxytriphenylamine. The reaction was stirred for 5 min under ice cooling and then allowed to warm up to room temperature.
The reaction was stopped by the addition of an aqueous saturated $NaHCO_3$ solution. The small amount of CH_3CN was removed under high-vacuum. Then an aqueous diluted solution of NaOH (8%) was added (pH > 9), converting all formed (**1**+4H)$^{4+}$ to (**1**+2H)$^{2+}$ (for subsequent transfer into the organic phase). A clear solution was obtained that was extracted several times with CH_2Cl_2. The combined organic phases were dried over Na_2SO_4, filtrated and condensed.

^{1}H NMR spectra (399.87 MHz, 295.1 K, CD_2Cl_2) for entry A

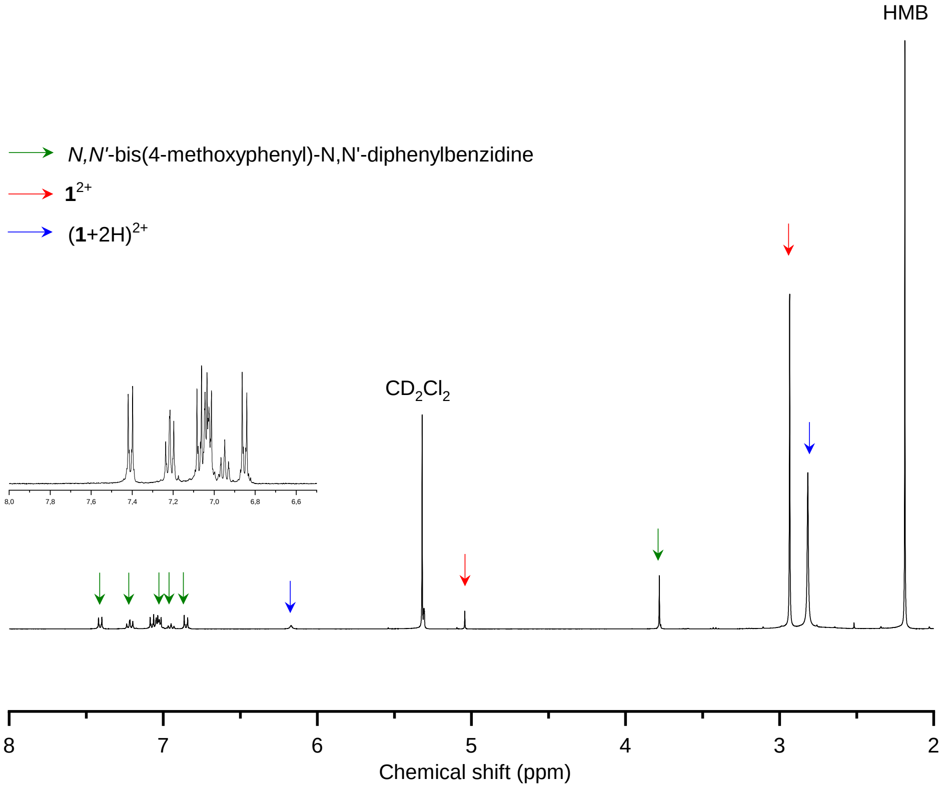

^{1}H NMR of *N,N′*-bis-(4-methoxyphenyl)-*N,N′*-diphenylbenzidine: (399.89 MHz, 295.1 K, CD_2Cl_2): δ = 3.78 (s, 6 H), 6.85 (d, *J* = 9.0 Hz, 4 H), 6.95 (t, *J* = 7.3 Hz, 2 H), 7.01 - 7.05 (m, 8 H), 7.07 (d, *J* = 9.0 Hz, 4 H), 7.22 (dd, *J* = 8.5; 7.4 Hz, 4 H), 7.41 (d, *J* = 8.7 Hz, 4 H) ppm.

The reaction was also monitored by UV-Vis spectroscopy in CH_2Cl_2 for a ratio substrate/GFA of 1:1

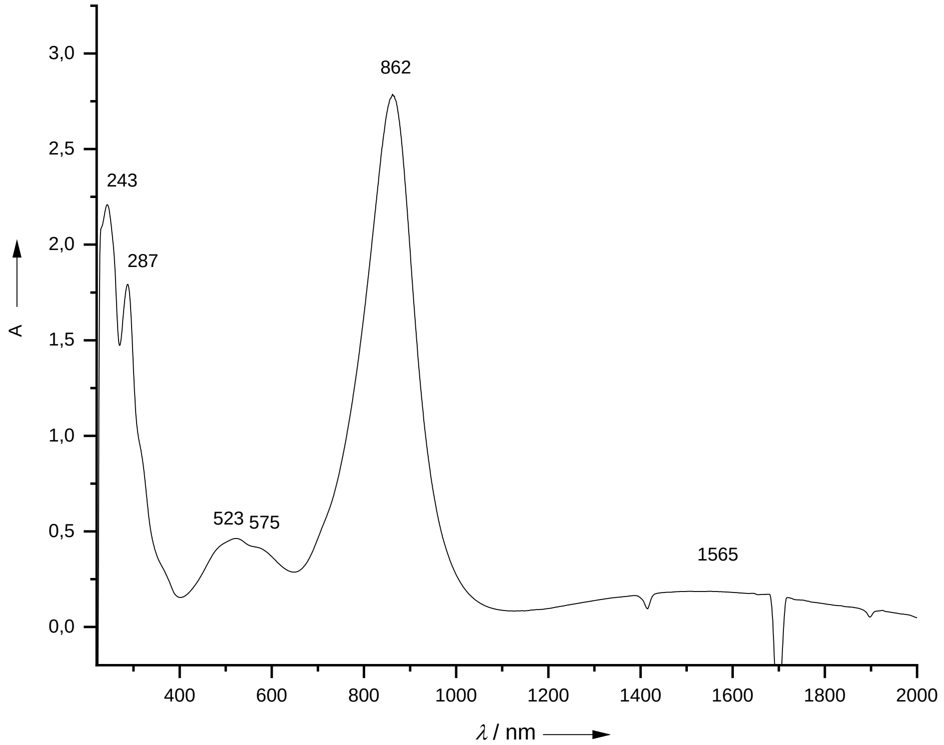

Reaction was also monitored by UV-Vis spectroscopy in CH_2Cl_2 for a ratio substrate/GFA of 1:0.65

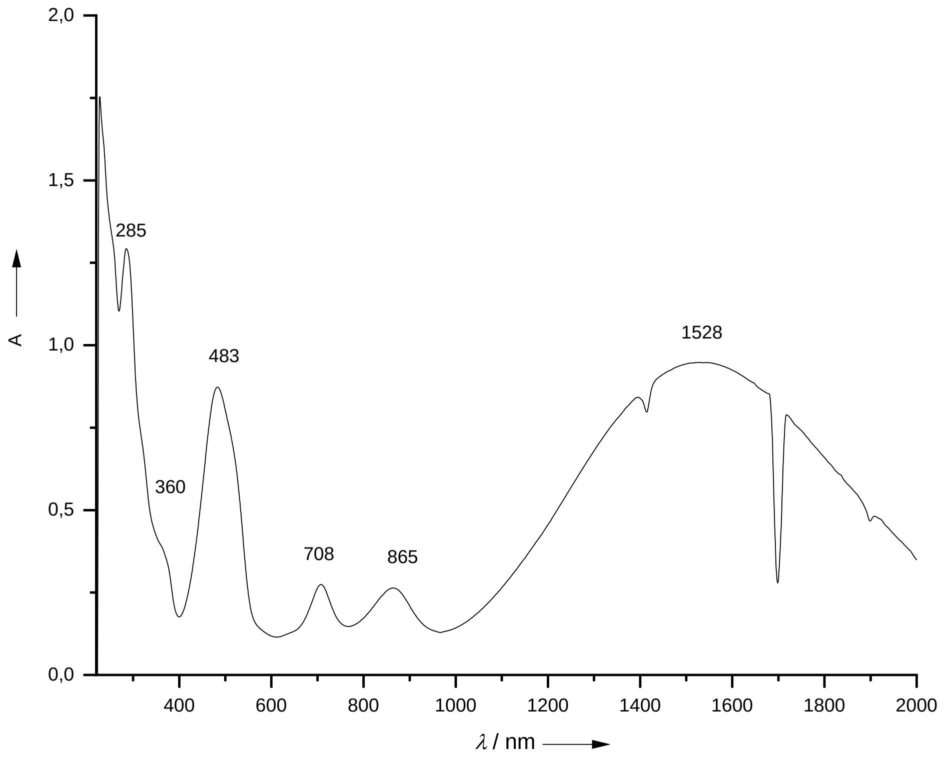

SCE potentials vs. Fc/Fc^+, measured with a scan rate of 100 $mV{\cdot}s^{-1}$ and $^nBu_4N(PF_6)$ as supporting electrolyte in CH_3CN solution

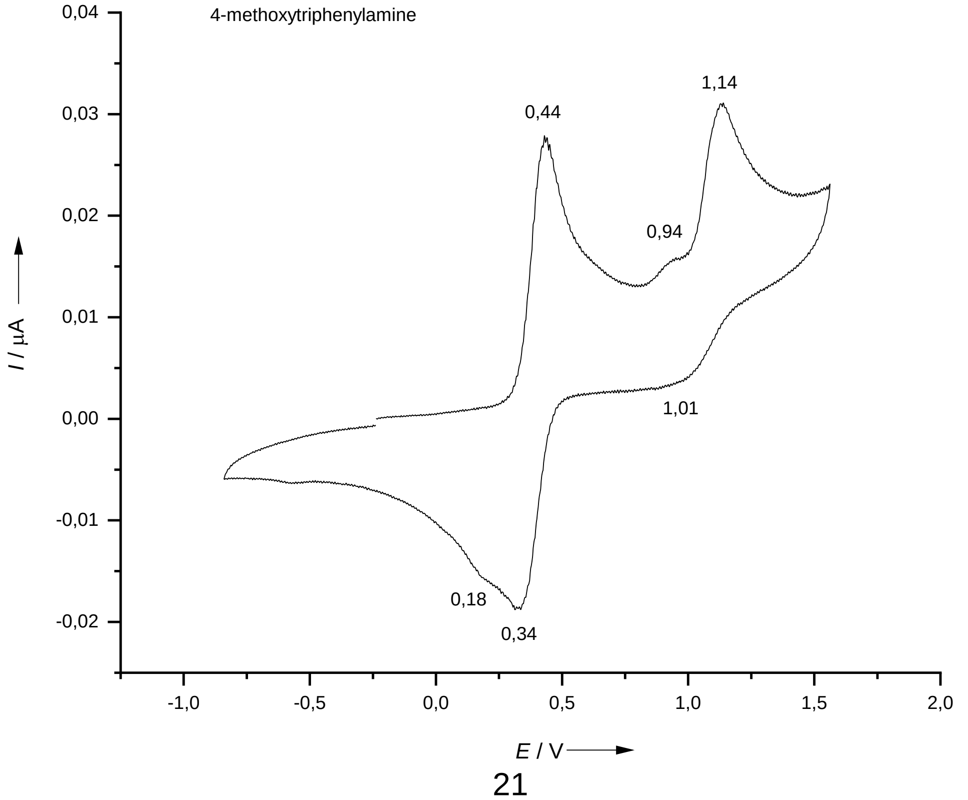

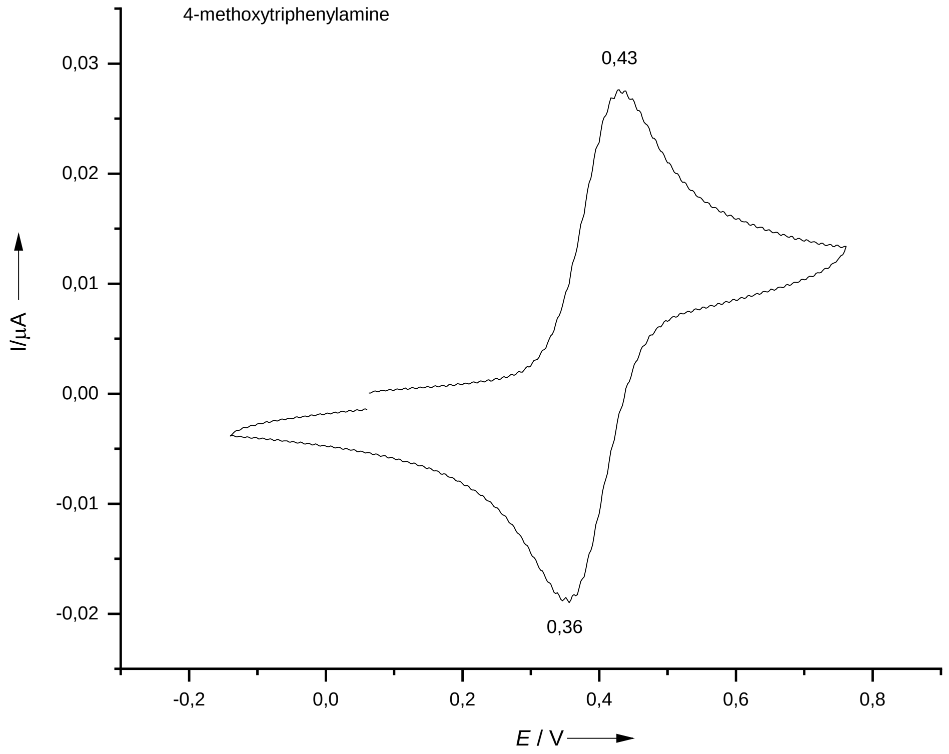

4-methoxytriphenylamine
0,43
0,36
0,03
0,02
0,01
0,00
-0,01
-0,02
I/µA
-0,2
0,0
0,2
0,4
0,6
0,8
E / V

6) Experimental details for oxidative coupling of triphenylamine to *N,N,N',N'*-tetraphenylbenzidine

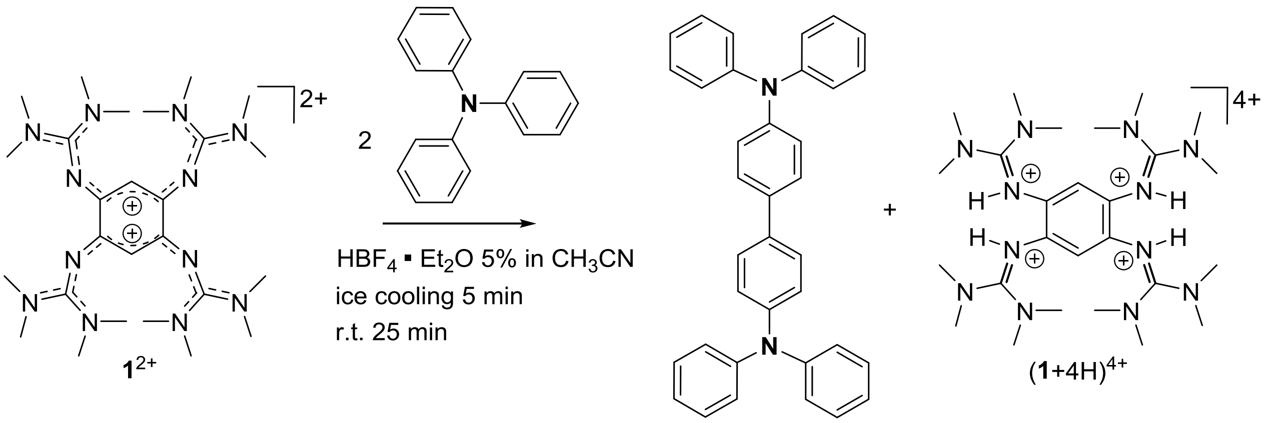

entry	A	B
**1**$(BF_4)_2$ [mg/µmol]	6.492/9.22	-
**1**$(PF_6)_2$ [mg/µmol]	-	8.496/10.35
triphenylamine [mg/mmol]	2.254/9.19	2.518/10.26
$HBF_4{\cdot}Et_2O$ (5% in CH_3CN) [mL/µmol]	0.5/182.3	0.5/182.3
HMB [mg/µmol]	1.150/7.09	2.072/12.77
reaction time (temperature)	5 min (ice cooling)<br>25 min (r.t.)	10 min (ice cooling)<br>15 min (r.t.)
conversion (rel. to $\mathbf{1}^{2+}$/($\mathbf{1}$+2H)$^{2+}$) [%]	>99	97
ratio (substrate/GFA)	1/1	1/1

entry	C	D	E
**1**$(BF_4)_2$ [mg/µmol]	-	-	5.720/8.12
**1**$(PF_6)_2$ [mg/µmol]	8.032/9.79	8.828/10.76	-
triphenylamine [mg/mmol]	1.386/5.65	2.954/12.04	3.088/12.59
$HBF_4{\cdot}Et_2O$ (5% in CH_3CN) [mL/µmol]	0.5/182.3	0.6/218.7	0.5/182.3
HMB [mg/µmol]	1.342/8.27	1.072/6.61	1.012/6.24
reaction time (temperature)	5 min<br>(ice cooling)<br>10 min (r.t.)	10 min<br>(ice cooling)<br>10 min (r.t.)	5 min<br>(ice cooling)<br>40 min (r.t.)
conversion (rel. to $\mathbf{1}^{2+}$/($\mathbf{1}$+2H)$^{2+}$) [%]	>99	88	62
ratio (substrate/GFA)	1/1.73	1/0.89	1/0.64

General protocol

In a Schlenk flask, 0.5 mL of a $HBF_4 \cdot Et_2O$ solution (5% in CH_3CN) was added under ice cooling in an Ar atmosphere to the reactants **1**$(BF_4)_2$ / **1**$(PF_6)_2$) and triphenylamine. The reaction was stirred for several minutes under ice cooling and then allowed to warm up to room temperature.
The reaction was stopped by the addition of an aqueous saturated $NaHCO_3$ solution. The small amount of CH_3CN was removed under high-vacuum. Then an aqueous diluted solution of NaOH (8%) was added (pH > 9), converting all formed (**1**+4H)$^{4+}$ to (**1**+2H)$^{2+}$ (for subsequent transfer into the organic phase). A clear solution was obtained that was extracted several times with CH_2Cl_2. The combined organic phases were dried over Na_2SO_4, filtrated and condensed.

Separation of 1(BF_4)$_2$ / [(1+2H)(BF_4)$_2$]

The residue was collected in diethylether and filtrated, allowing the isolation of the coupling product in the filtrate. The residue in the filter, consisting of **1**$(BF_4)_2$/[(**1**+2H)$(BF_4)_2$], was dissolved in CH_3CN for recycling of the guanidine PCET reagent.

^{1}H NMR spectra (399.89 MHz, 295.1 K, CD_2Cl_2) for entry A

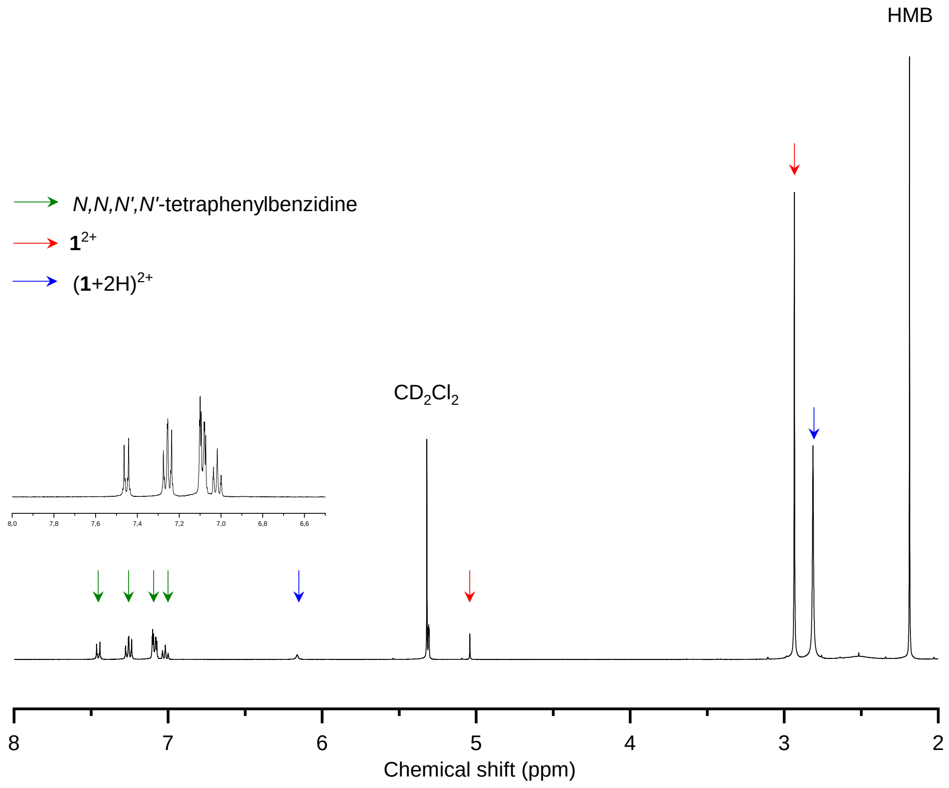

^{1}H NMR *of N,N,N',N'*-tetraphenylbenzidine: (399.89 MHz, 295.1 K, CD_2Cl_2): δ = 7.00 (t, *J* = 7.3 Hz, 4 H), 7.05 - 7.08 (m, 12 H), 7.23 (dd, *J* = 8.5; 7.4 Hz, 8 H), 7.43 (d, *J* = 8.7 Hz, 4 H) ppm.

Reaction was also monitored by UV-Vis spectroscopy in CH_2Cl_2 for a ratio substrate/GFA of 1:1

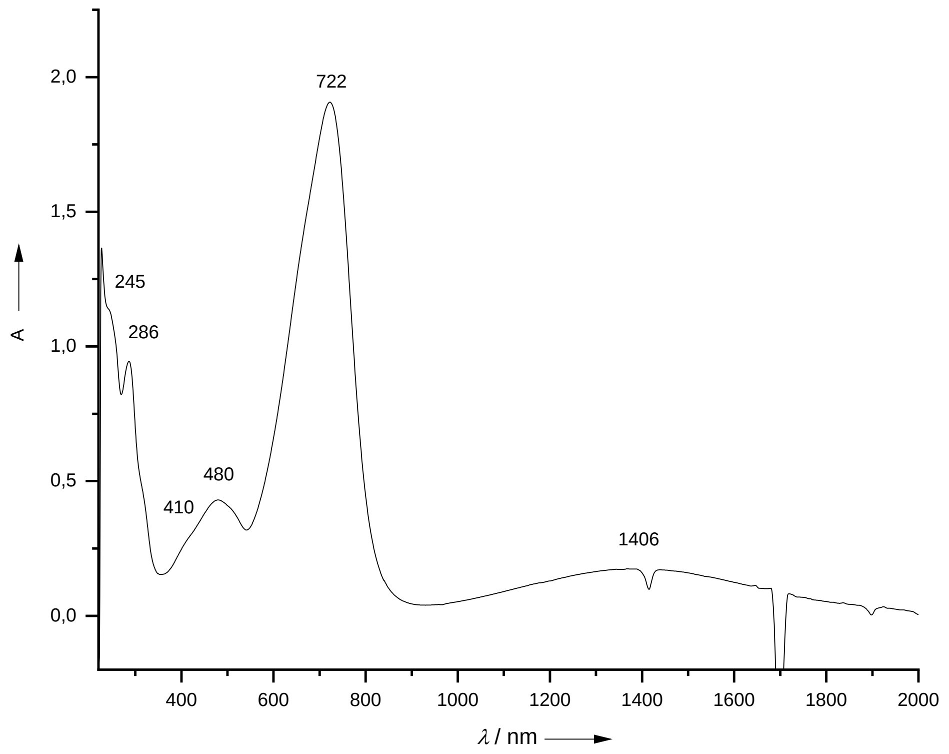

Reaction was also monitored by UV-Vis spectroscopy in CH_2Cl_2 for a ratio substrate/GFA of 1:0.64

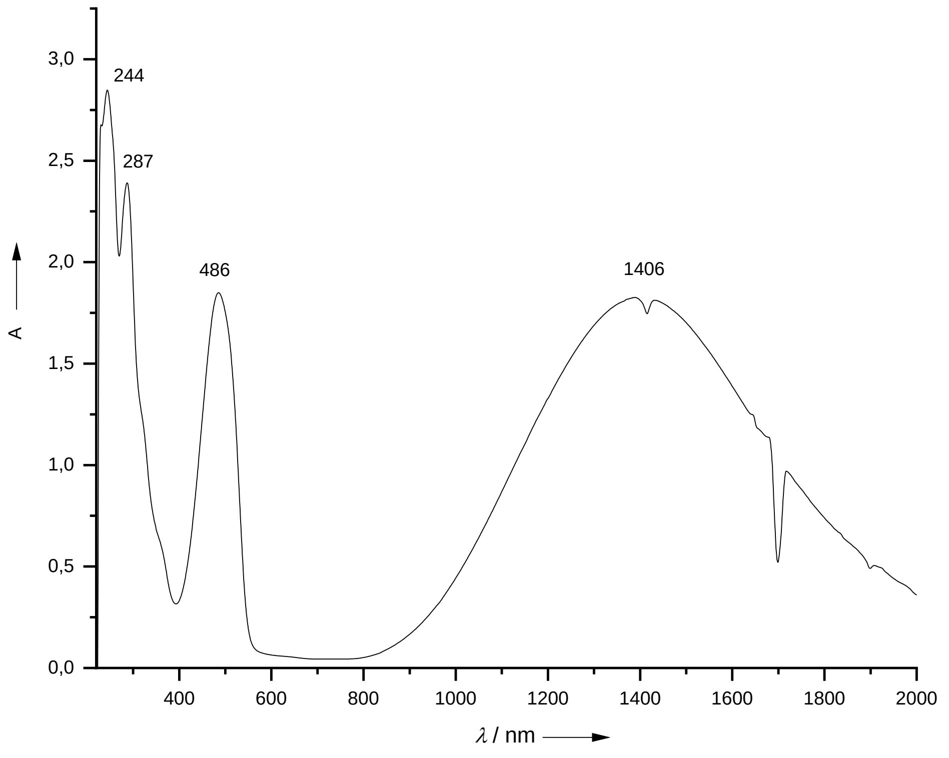

Triphenylamine reactions with small quantities of HBF_4

entry	**A** -NMR experiment	**B**	**C**	**D**
**1**$(BF_4)_2$ [mg/µmol]	7.098/10.08	8.520/12.10	-	9.584/13.61
**1**$(PF_6)_2$ [mg/µmol]			8.320/10.14	
triphenylamine [mg/mmol]	4.856/19.79	6.052/24.67	5.046/20.57	6.882/28.05
$HBF_4 \cdot Et_2O$ (5% in CH_3CN) [mL/µmol]	[a]0.05/18.3<br>[b]0.27/98.4	0.22/80.2	0.18/65.6	0.25/91.1
CH_3CN/CD_3CN (NMR experiment) [mL]	0.45	1	1	1
HMB [mg/µmol]	0.964/5.94	2.090/12.88	3.024/18.63	1.988/12.25
reaction time (temperature)	not determined (instantly)	1 h (ice cooling)	5 min (ice cooling)<br>2.5 h (r.t.)	24 h (r.t.)
ratio (substrate/GFA)	1/0.51	1/0.49	1/0.49	1/0.49

[a]first addition; [b]second addition

Reaction in an NMR tube (entry A)

In an NMR tube, the reactants **1**$(BF_4)_2$ and triphenylamine (and HMB) were dissolved in an Ar atmosphere in 0.45 mL CD_3CN. An NMR spectrum was recorded, showing that no reaction took place. Then 0.05 mL of a $HBF_4 \cdot Et_2O$ solution (5% in CD_3CN) were dropwise added with a syringe and an NMR spectrum taken from the reaction mixture. After a second (dropwise) addition of 0.27 mL of the $HBF_4 \cdot Et_2O$ solution (5% in CD_3CN) another NMR spectrum was recorded.

Reactions in Schlenk flasks (entries B - D)

In a Schlenk flask, a $HBF_4 \cdot Et_2O$ solution (5% in CH_3CN) was added with a syringe under ice cooling in an Ar atmosphere to a solution of the reactants **1**$(BF_4)_2$ / **1**$(PF_6)_2$) and triphenylamine in 1 mL CH_3CN. The reaction was stirred for several minutes under ice cooling and then allowed to warm up to room temperature for further reaction.

Work-up (entries B - D)

The reaction was stopped by the addition of an aqueous saturated $NaHCO_3$ solution. The small amount of CH_3CN was removed under high-vacuum. For entry B, an aqueous diluted solution of NaOH (8%) was added (pH > 9), converting all formed (**1**+4H)$^{4+}$ to (**1**+2H)$^{2+}$ (for subsequent transfer into the organic phase). In the case of entries **C** and **D**, a larger amount of $NaHCO_3$ solution was used in place for the NaOH solution. A colourless and clear solution was obtained that was extracted several times with CH_2Cl_2. The combined organic phases were dried over Na_2SO_4, filtrated and condensed.

^{1}H NMR experiment entry A (199.87 MHz, 298 K, CD_3CN): reaction of $1(BF_4)_2$ and triphenylamine without addition of the $HBF_4{\cdot}Et_2O$ solution (5% in CD_3CN)

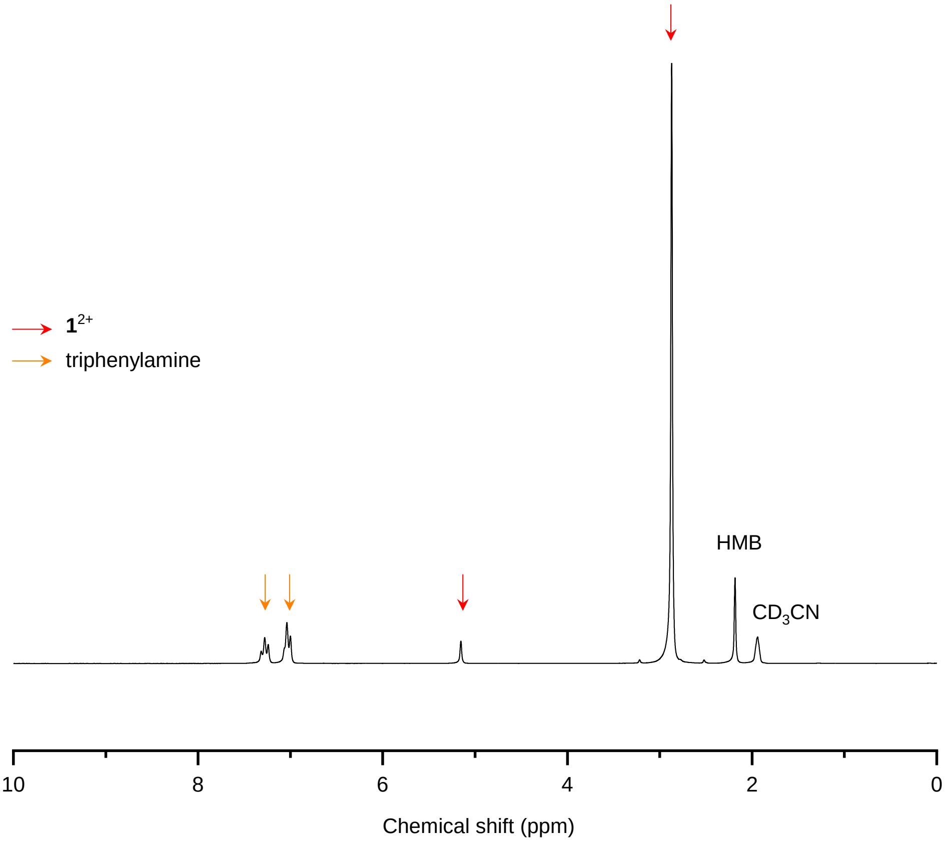

^{1}H NMR experiment entry A (199.87 MHz, 298 K, CD_3CN): reaction of $1(BF_4)_2$ and triphenylamine after first addition of the $HBF_4{\cdot}Et_2O$ solution (5% in CD_3CN)

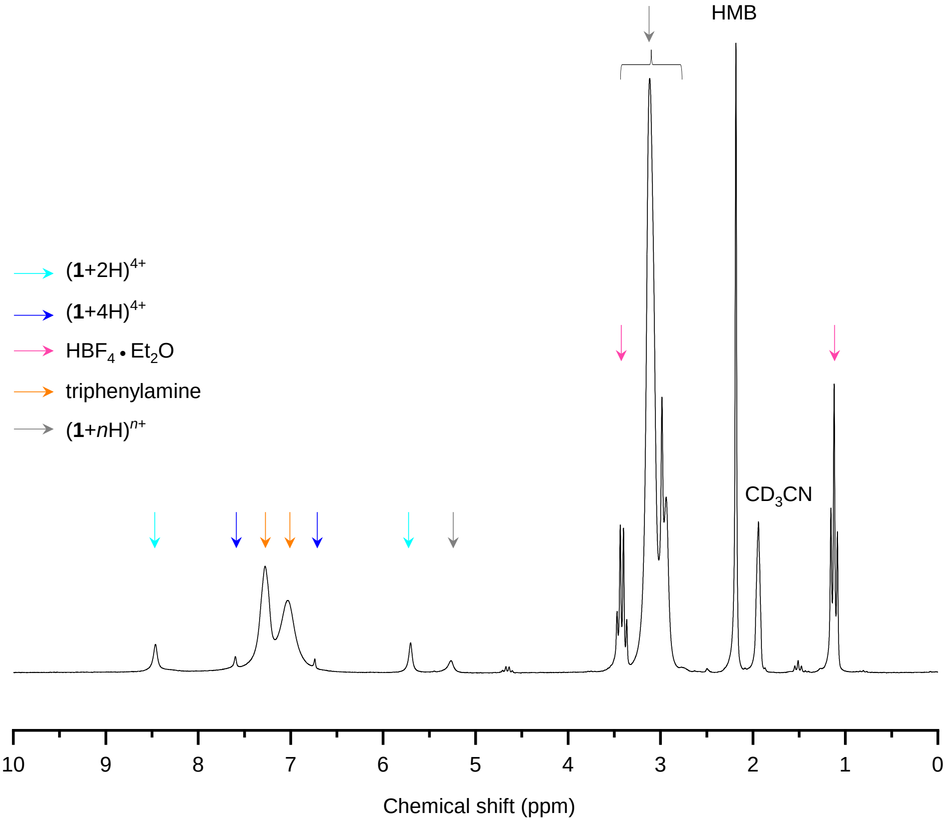

^1H NMR experiment entry A (199.87 MHz, 298 K, CD_3CN): reaction of 1(BF_4)$_2$ and triphenylamine after second addition of the $HBF_4 \cdot Et_2O$ solution (5% in CD_3CN)

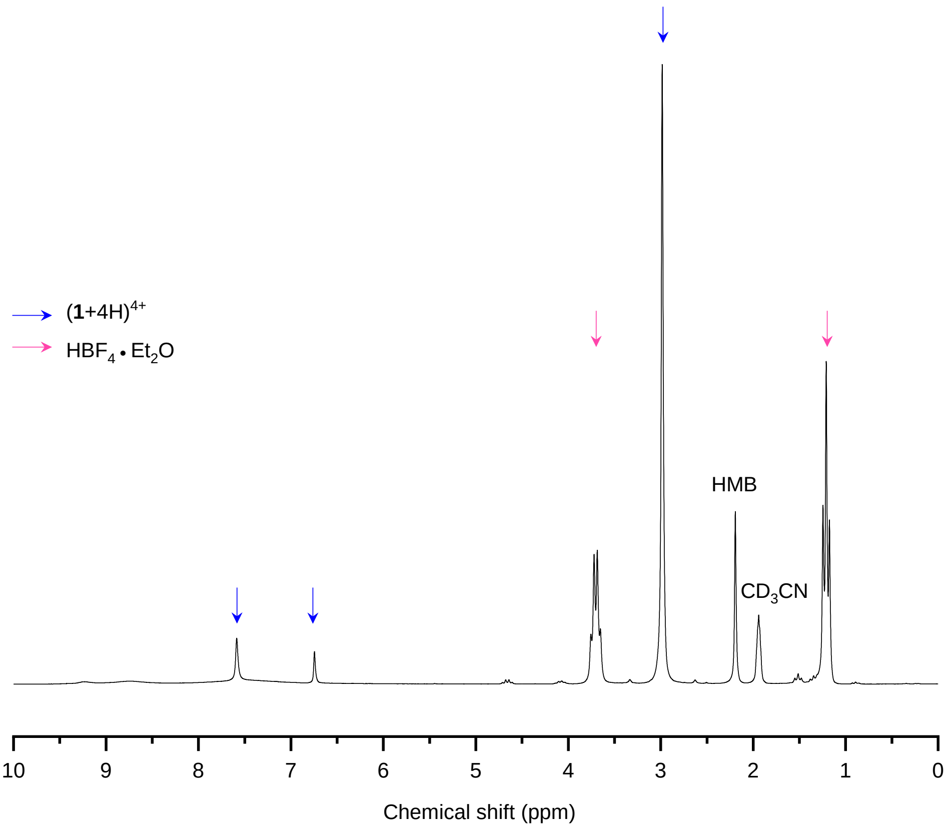

^1H NMR spectra (399.89 MHz, 295.6 K, CD_2Cl_2) for entry B

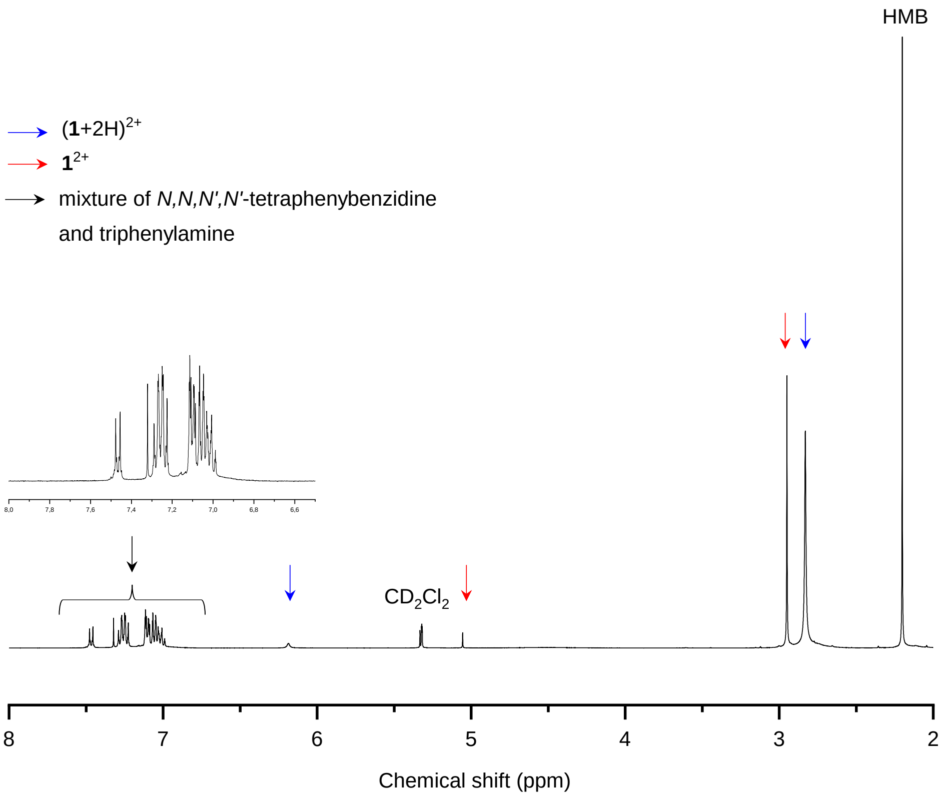

^{1}H NMR spectra (399.89 MHz, 294.9 K, CD_2Cl_2) for entry C

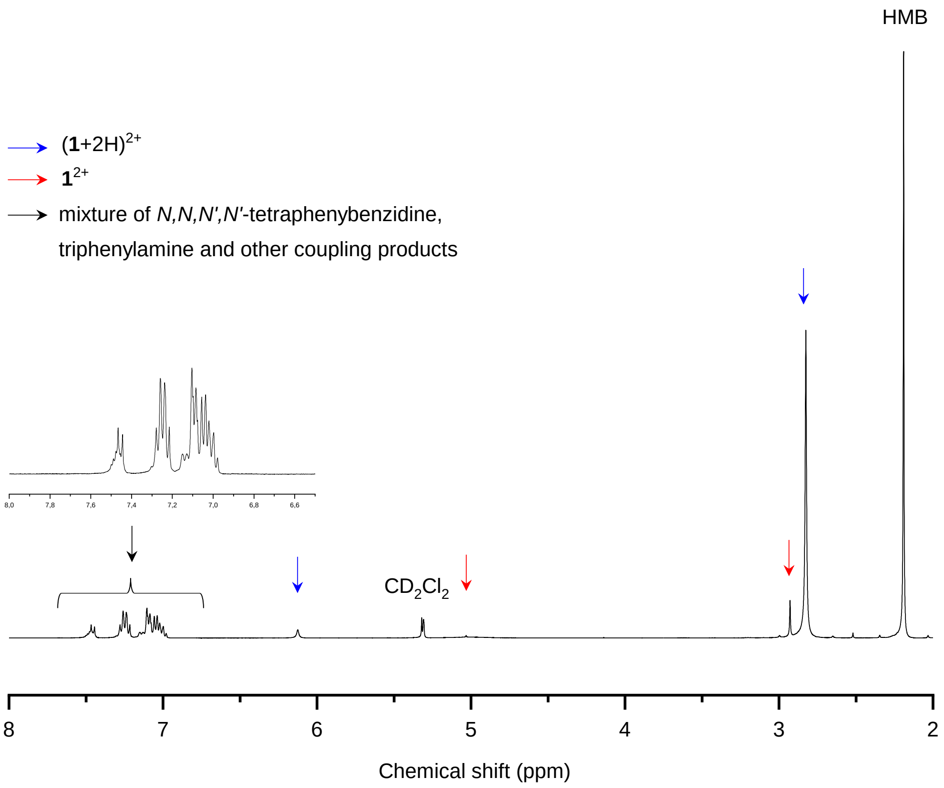

^{1}H NMR spectra (399.89 MHz, 295.6 K, CD_2Cl_2) for entry D

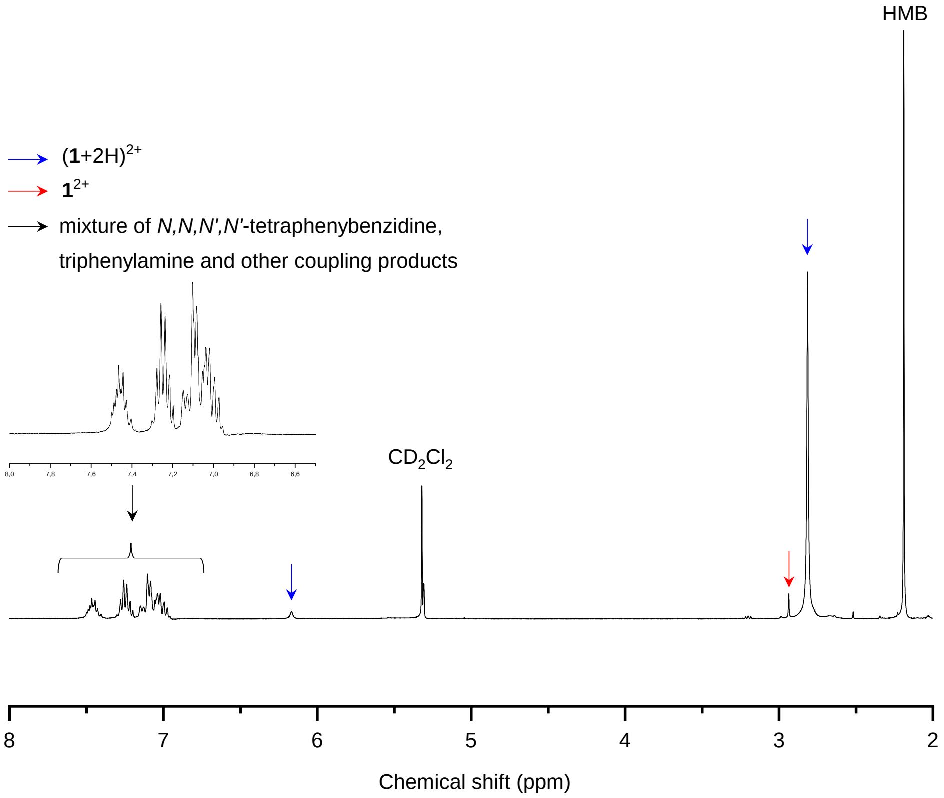

Reaction was monitored by UV-Vis spectroscopy in CH_2Cl_2 for entry B

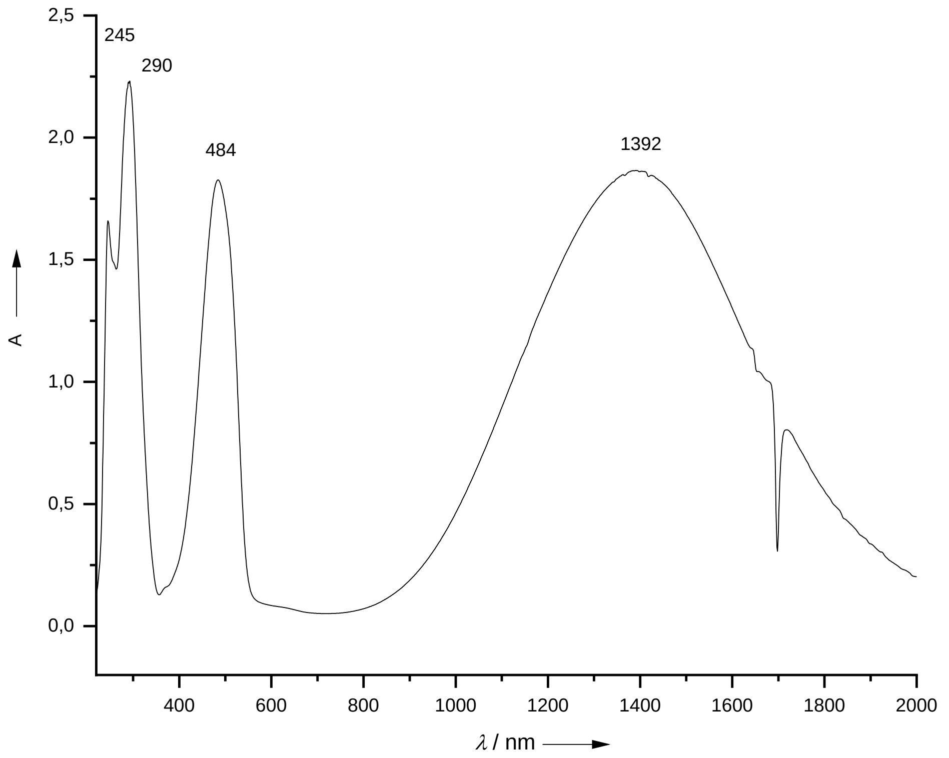

SCE potentials vs. Fc/Fc$^+$, measured with a scan rate of 100 mV·s^{-1} and ^{n}Bu$_4$N(PF$_6$) as supporting electrolyte in CH_3CN

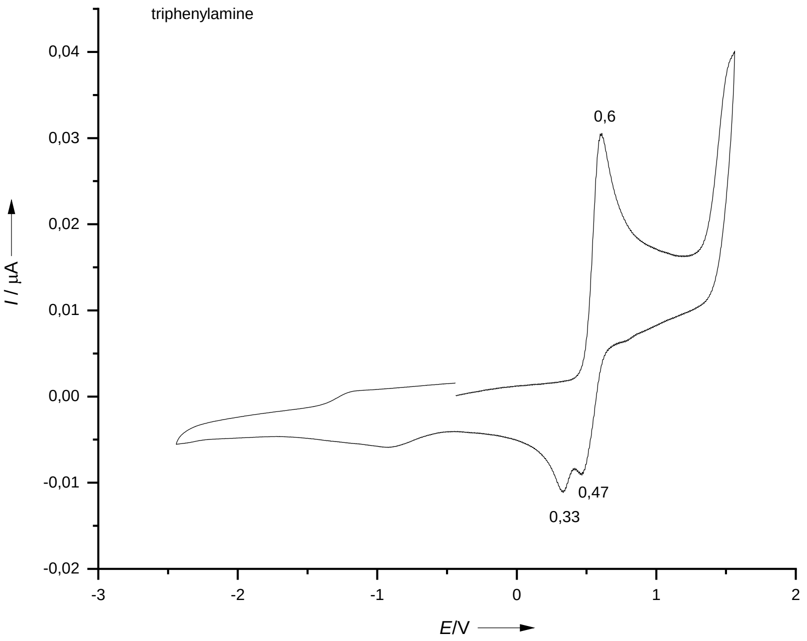

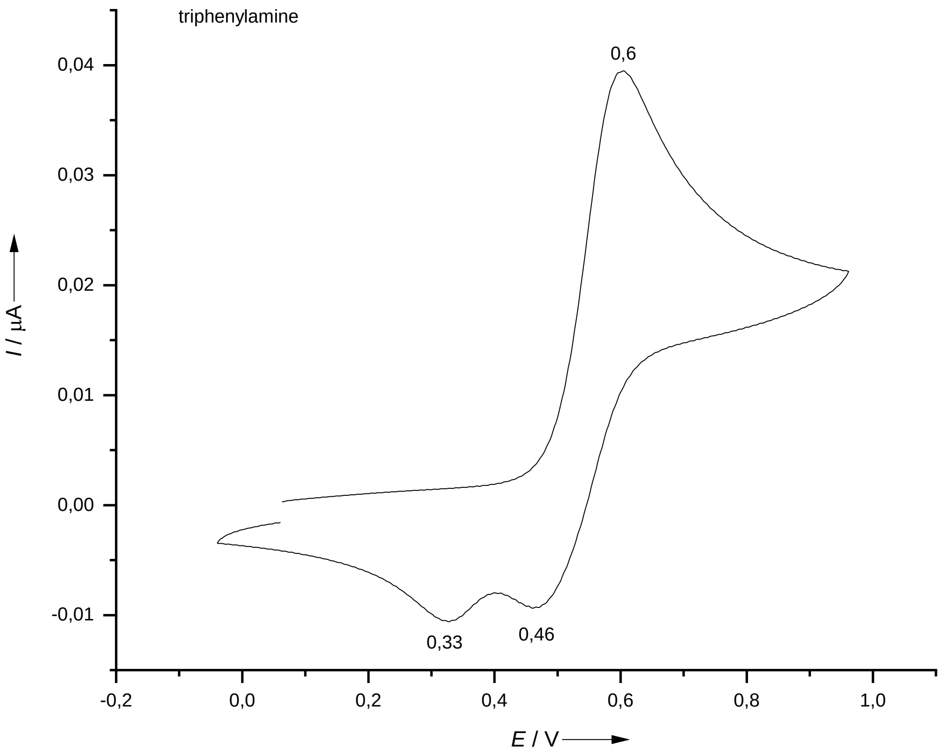
triphenylamine
0,6
0,33
0,46
0,04
0,03
0,02
0,01
0,00
-0,01
I / µA
-0,2
0,0
0,2
0,4
0,6
0,8
1,0
E / V

7) Experimental Details for oxidative coupling of 4,4'-dibromotriphenylamine to *N,N,N',N'*-tetrakis(4-bromophenyl)-benzidine

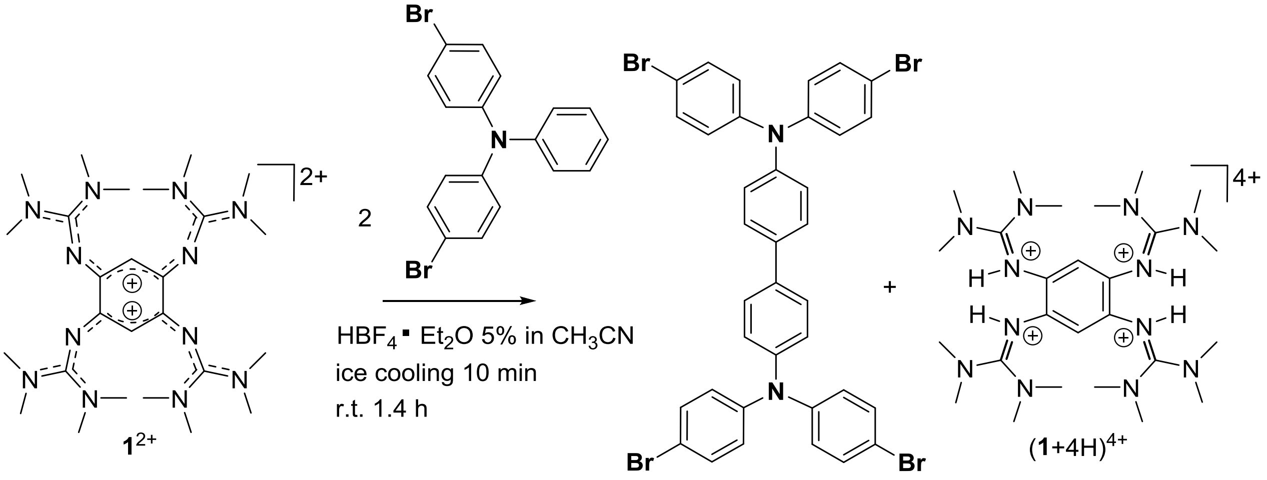

entry	A	B	C
**1**$(BF_4)_2$ [mg/µmol]	5.376/7.63	-	-
**1**$(PF_6)_2$ [mg/µmol]	-	6.374/7.77	5.800/7.07
4,4'-dibromotriphenylamine [mg/mmol]	3.078/7.64	3.132/7.77	4.47/11.09
$HBF_4{\cdot}Et_2O$ (5% in CH_3CN)[mL/µmol]	0.5/182.3	0.5/182.3	0.11/40.1
HMB [mg/µmol]	1.974/12.16	1.562/9.63	1.696/10.45
CH_3CN [mL]	-	-	0.6
reaction time (temperature)	10 min (ice cooling)<br>1.4 h (r.t.)	5 min (ice cooling)<br>1.2 h (r.t.)	23 h (r.t.)
conversion (rel. to **1**$^{2+}$/(**1**+2H)$^{2+}$)[%]	95	92	89
ratio (substrate/GFA)	1/1	1/1	1/0.64

General protocol

Entries **A** and **B**:

In a Schlenk flask, 0.5 mL of a $HBF_4{\cdot}Et_2O$ solution (5% in CH_3CN) was added with a syringe under ice cooling in an Ar atmosphere to a solution of **1**$(BF_4)_2$ / **1**$(PF_6)_2$) and 4,4'-dibromotriphenylamine in 1 mL CH_3CN. The reaction was stirred for several minutes under ice cooling and then allowed to warm up to room temperature for further reaction.

Entry **C**:

In a Schlenk flask, 0.11 mL (40.1 µmol) of a $HBF_4 \cdot Et_2O$ solution (5% in CH_3CN) was added with a syringe in an Ar atmosphere to a solution of **1**($(PF_6)_2$) and 4,4'-dibromotriphenylamine in 0.6 mL CH_3CN. The reaction was stirred for 23 h at room temperature.

Work-up (for all experiments):

The reaction was stopped by the addition of an aqueous saturated $NaHCO_3$ solution. The small amount of CH_3CN was removed under high-vacuum. An aqueous diluted solution of NaOH (8%) was added (pH > 9), converting all formed $(\mathbf{1}+4H)^{4+}$ to $(\mathbf{1}+2H)^{2+}$ (for subsequent transfer into the organic phase). A clear solution was obtained that was extracted several times with CH_2Cl_2. The combined organic phases were dried over Na_2SO_4, filtrated and condensed.

^{1}H NMR spectra (600.13 MHz, 295.2 K, CD_2Cl_2) for entry A

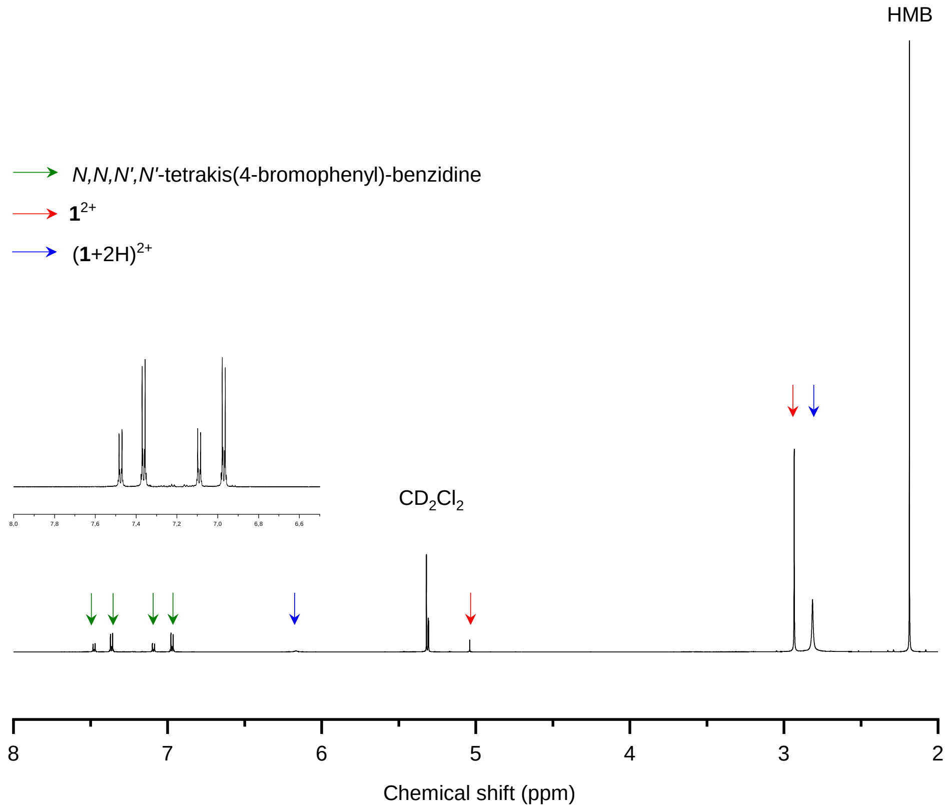

^{1}H NMR of *N,N,N',N'*-tetrakis(4-bromophenyl)-benzidine: (600.13 MHz, 295.2 K, CD_2Cl_2): δ = 6.97 (d, J = 8.8 Hz, 8 H), 7.09 (d, J = 8.6 Hz, 4 H), 7.36 (d, J = 8.8 Hz, 8 H), 7.48 (d, J = 8.6 Hz, 4 H ppm

Reaction was also monitored by UV-Vis spectroscopy in CH_2Cl_2: entry A

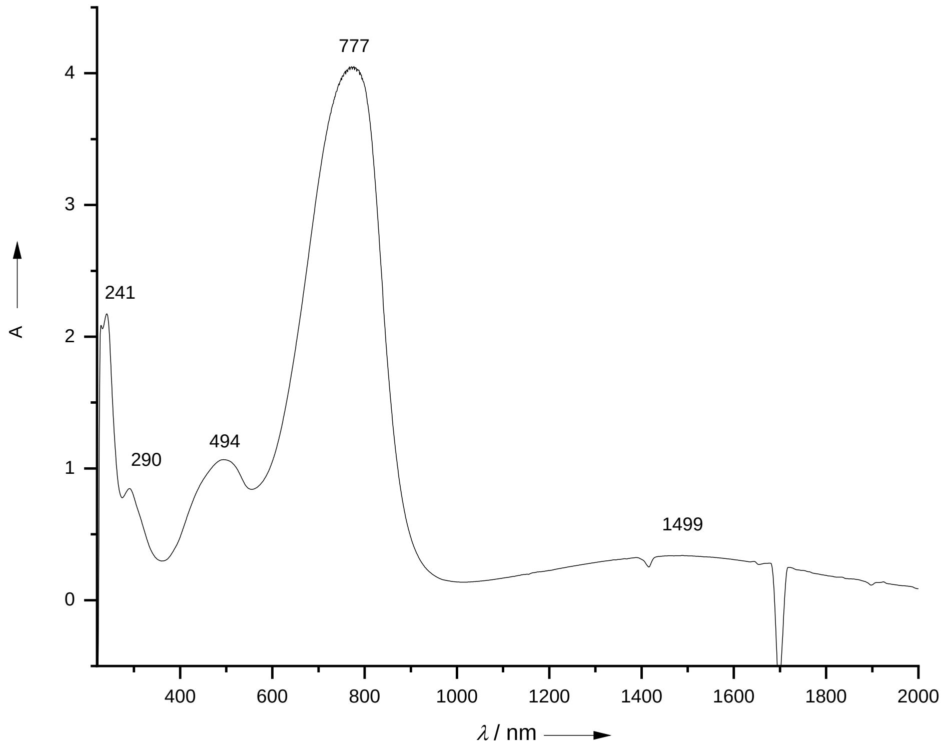

^{1}H NMR spectra (600.13 MHz, 295.2 K, CD_2Cl_2) for entry C

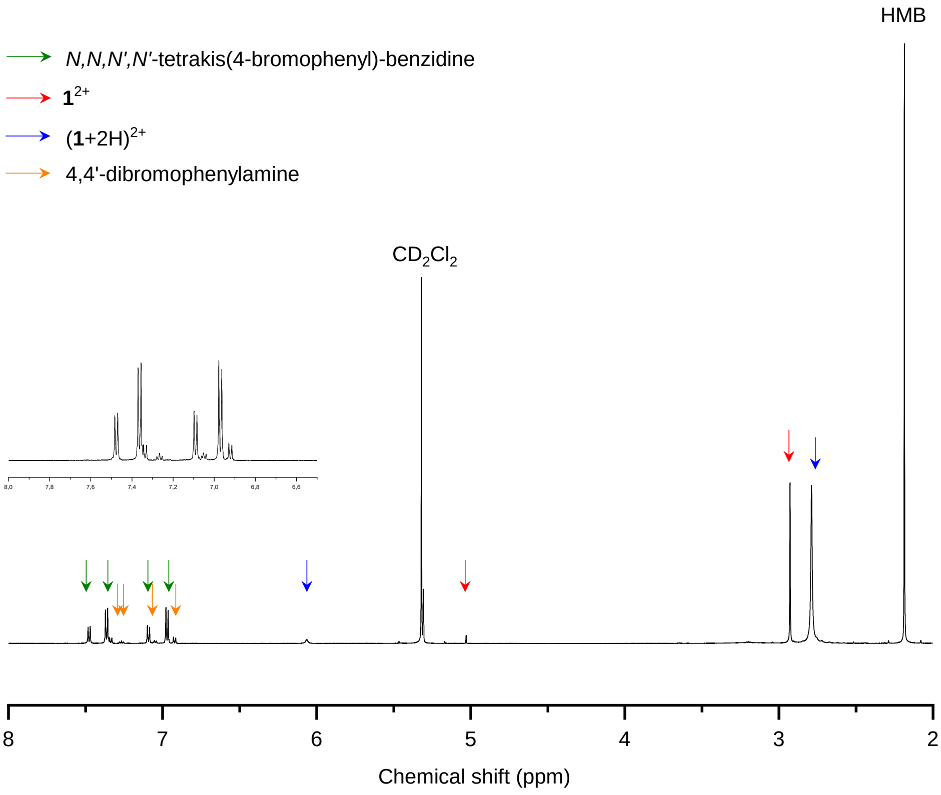

SCE potentials vs. Fc/Fc^{+}, measured with a scan rate of 100 mV·s^{-1} and $^{n}Bu_4N(PF_6)$ as supporting electrolyte in CH_3CN

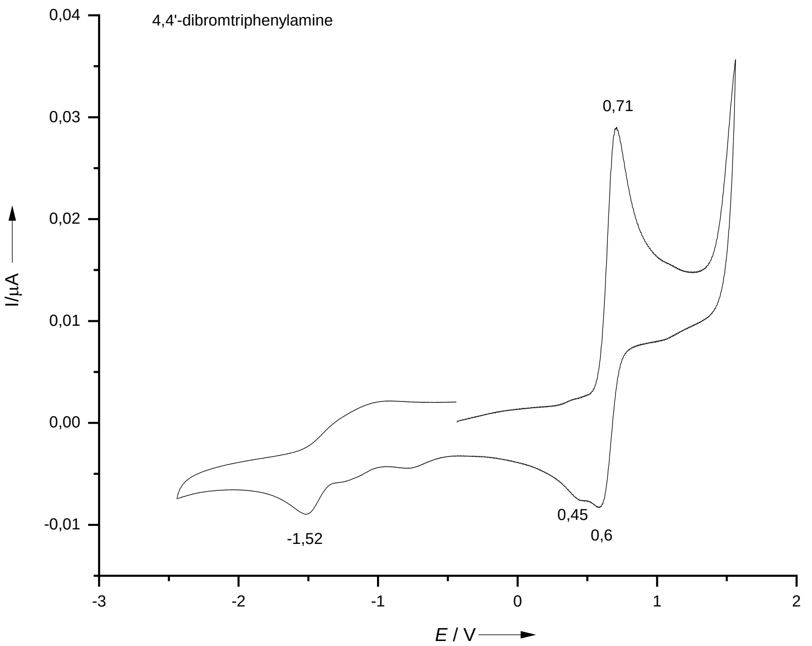

SCE potentials vs. Fc/Fc^{+}, measured with a scan rate of 50 mV·s^{-1} and $^{n}Bu_4N(PF_6)$ as supporting electrolyte in CH_3CN

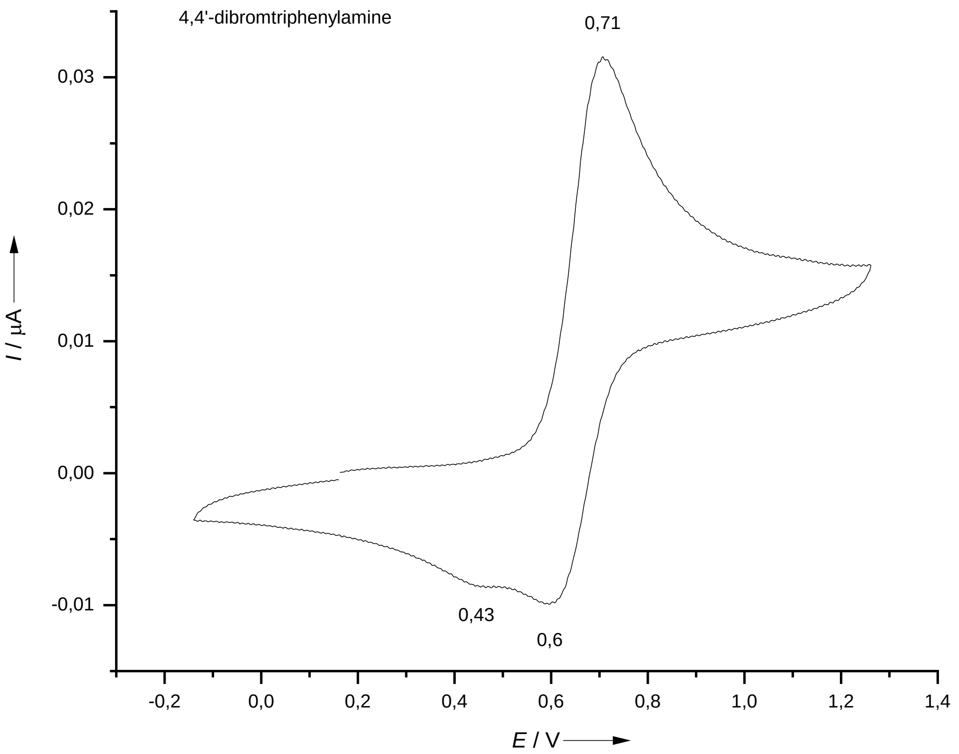

8) Experimental Details for oxidative coupling of 4-nitrotriphenylamine to *N,N'*-bis(4-nitrophenyl)-*N,N'*-diphenylbenzidine

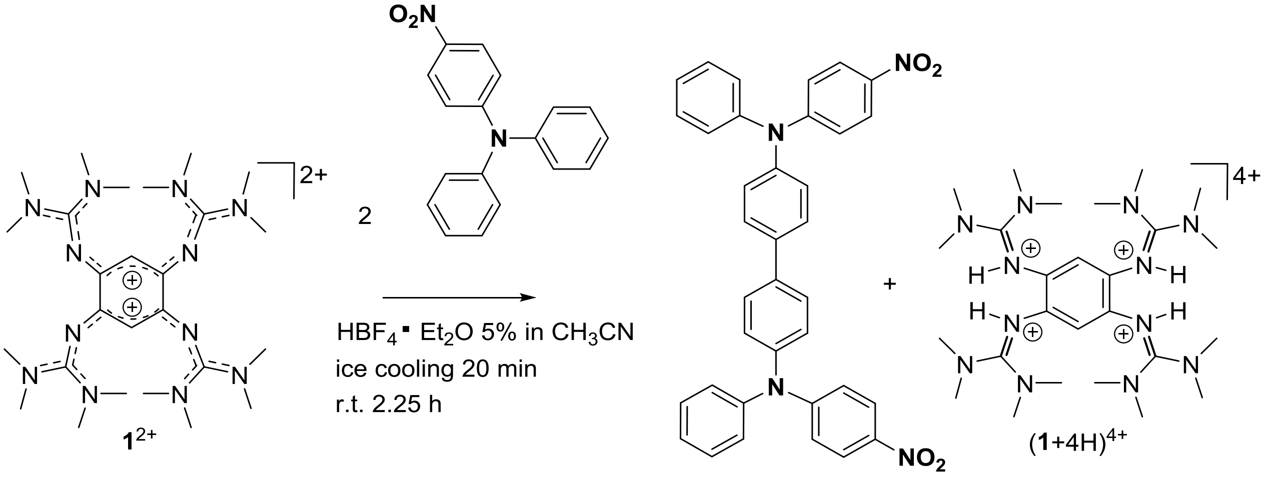

entry	A	B	C
**1**$(BF_4)_2$ [mg/µmol]	5.60/7.95	9.464/13.44	-
**1**$(PF_6)_2$ [mg/µmol]	-	-	10.68/13.01
4-nitrotriphenylamine [mg/mmol]	2.244/7.73	2.622/9.03	2.51/8.65
$HBF_4{\cdot}Et_2O$ (5% in CH_3CN)[mL/µmol]	0.5/182.3	0.5/182.3	0.5/182.3
HMB [mg/µmol]	1.974/12.16	1.136/7.00	1.696/10.45
reaction time (temperature)	20 min (ice cooling) 2.25 h (r.t.)	10 min (ice cooling) 1 h (r.t.)	10 min (ice cooling) 1 h (r.t.)
conversion (rel. to $\mathbf{1}^{2+}$/($\mathbf{1}$+2H)$^{2+}$)[%]	95	95	98
ratio (substrate/GFA)	1/1.03	1/1.49	1/1.5

General protocol

In a Schlenk flask, 0.5 mL of a $HBF_4{\cdot}Et_2O$ solution (5% in CH_3CN) was added under ice cooling in an Ar atmosphere to the reactants **1**$(BF_4)_2$ / **1**$(PF_6)_2$) and 4-nitrotriphenylamine. The reaction was stirred for several minutes under ice cooling and then allowed to warm up to room temperature for further reaction.
The reaction was stopped by the addition of an aqueous saturated $NaHCO_3$ solution. The small amount of CH_3CN was removed under high-vacuum. Then an aqueous diluted solution of NaOH (8%) was added (pH > 9), converting all formed (**1**+4H)$^{4+}$ to (**1**+2H)$^{2+}$ (for subsequent transfer into the organic phase). A clear solution was obtained that was extracted several times with CH_2Cl_2. The combined organic phases were dried over Na_2SO_4, filtrated and condensed.

^{1}H NMR spectra (399.89.13 MHz, 295.1 K, CD_2Cl_2) for entry A

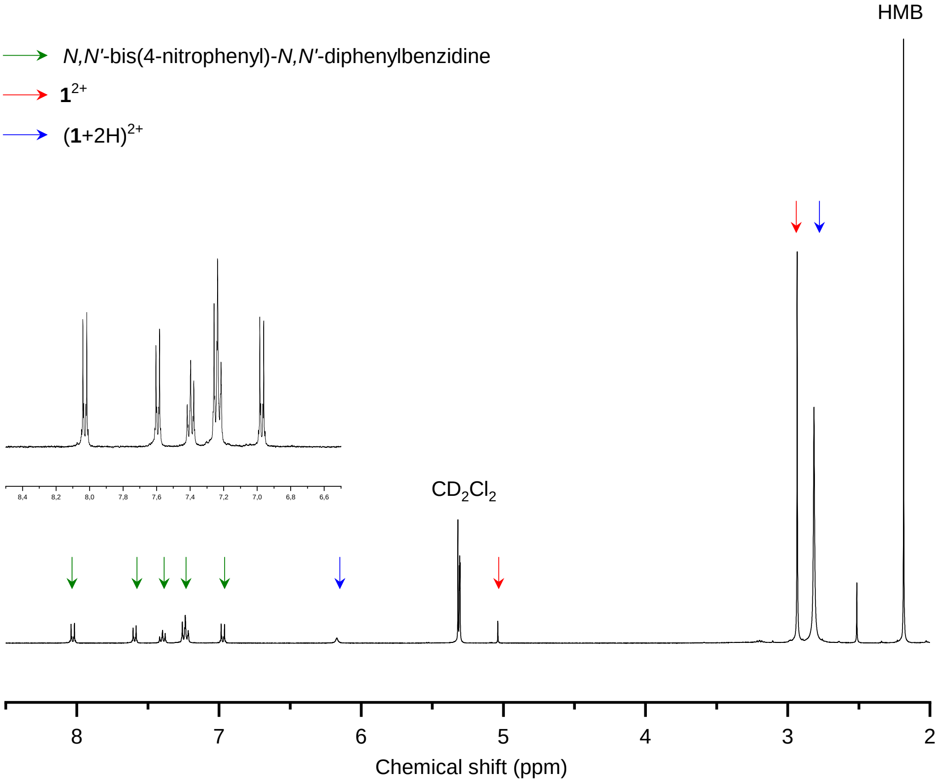

^{1}H NMR *N,N′*-bis(4-nitrophenyl)-*N,N′*-diphenylbenzidine: (399.89 MHz, 295.1 K, CD_2Cl_2): δ = 6.97 (d, *J* = 9.3 Hz, 4 H), 7.24 (t, *J* = 8.3 Hz, 10 H), 7.40 (t, *J* = 7.8 Hz, 4 H), 7.59 (d, *J* = 8.7 Hz, 4 H), 8.03 (d, *J* = 9.4 Hz, 4 H) ppm.

Reaction was also monitored by UV-Vis spectroscopy in CH_2Cl_2: entry A

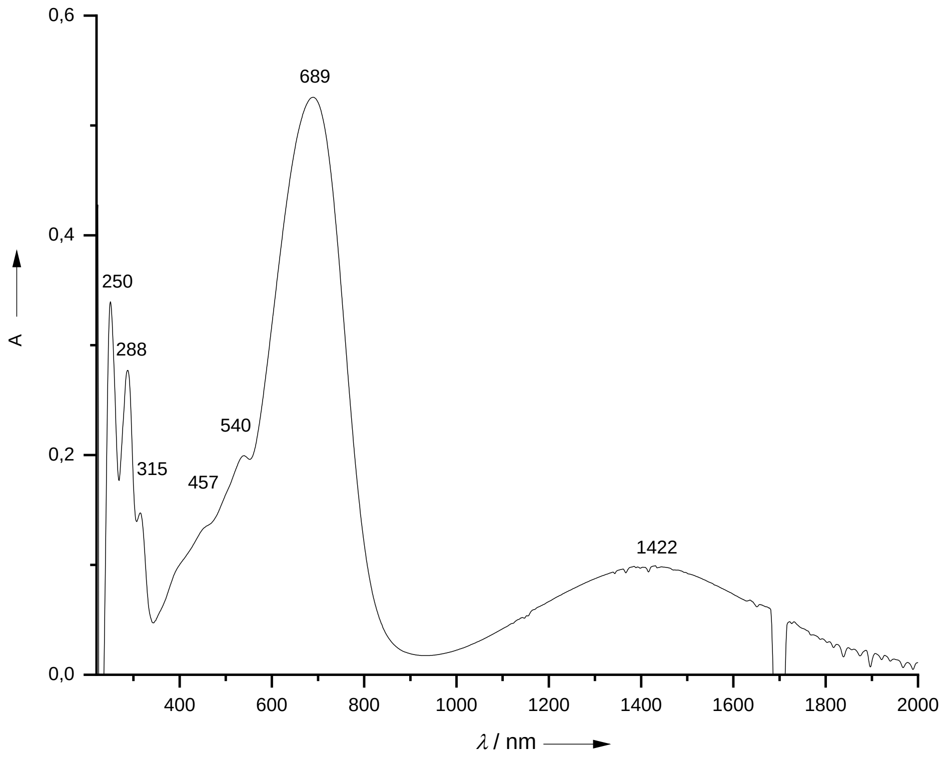

SCE potentials vs. Fc/Fc^+, measured with a scan rate of 100 $mV{\cdot}s^{-1}$ and $^nBu_4N(PF_6)$ as supporting electrolyte in CH_3CN

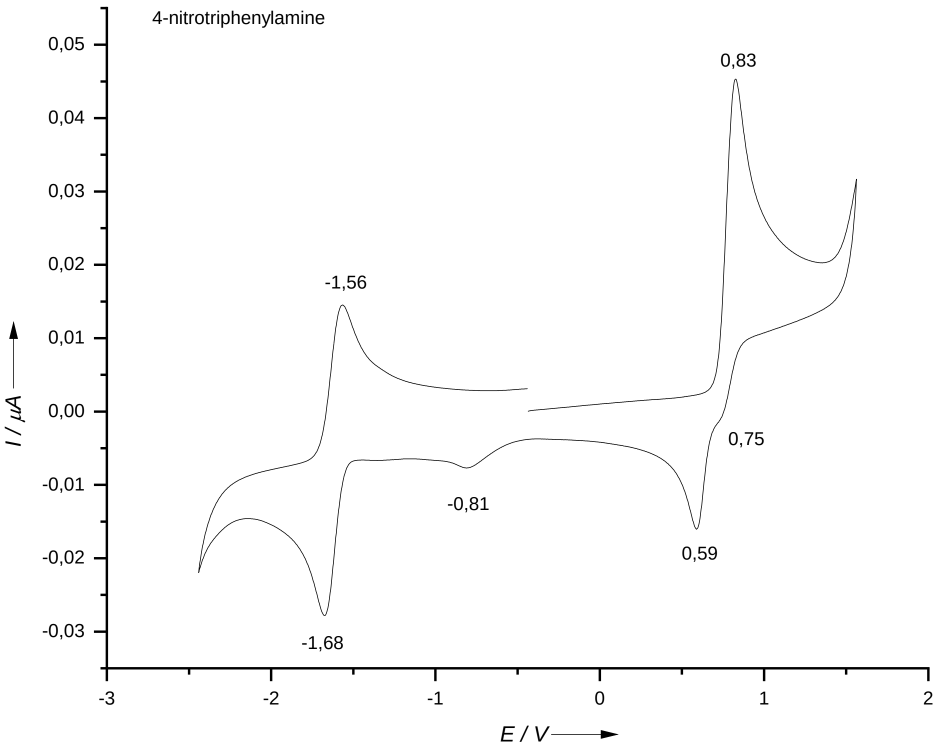

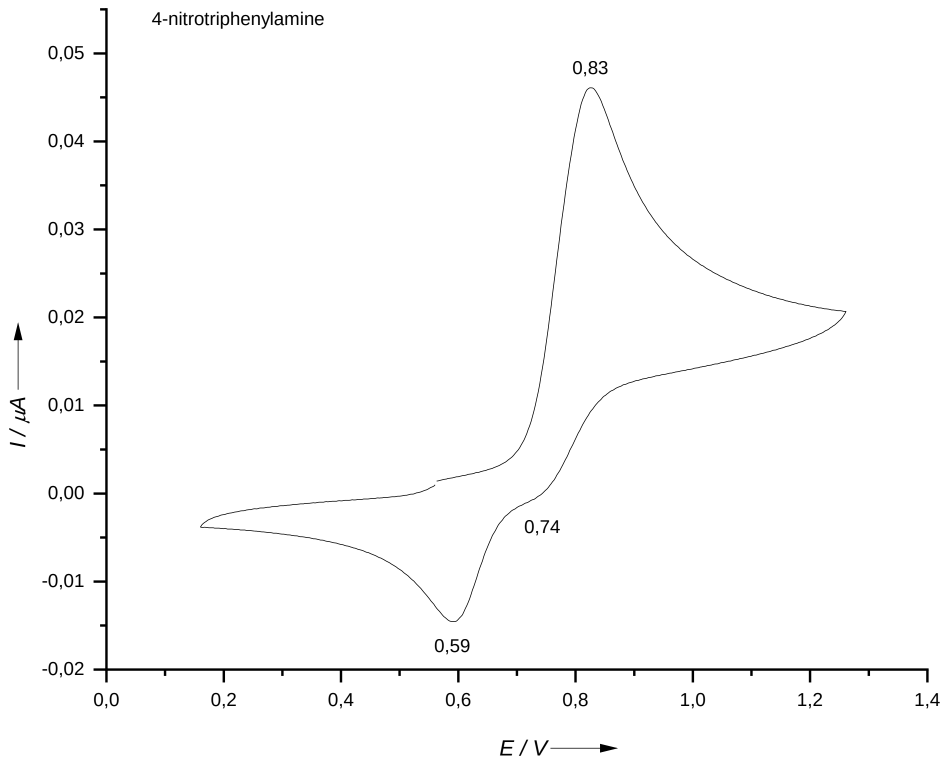

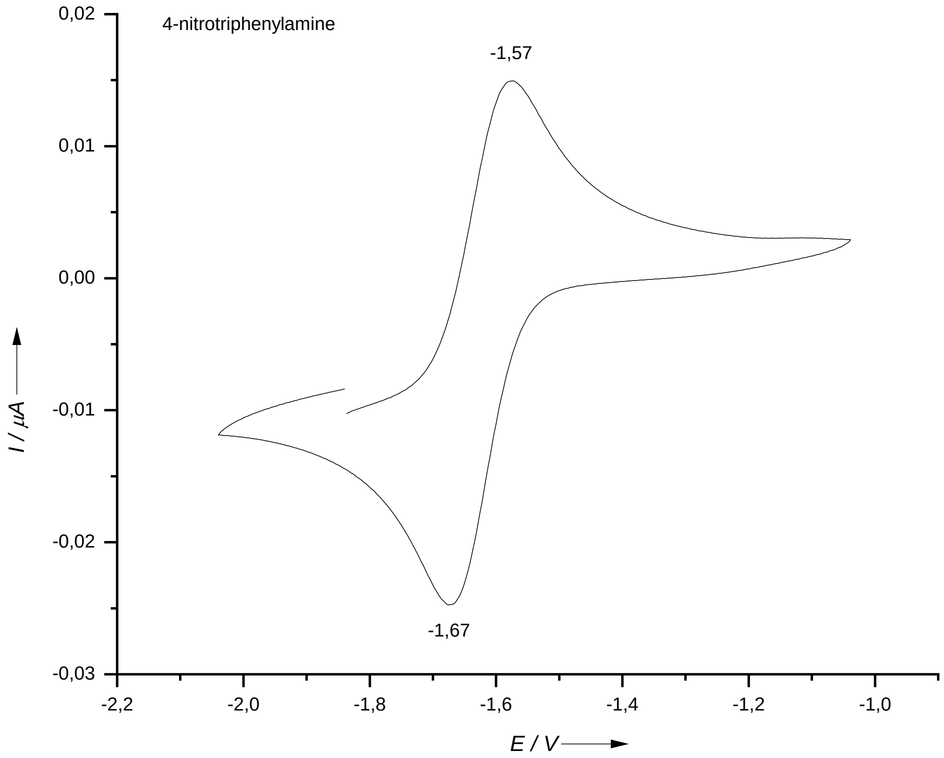
4-nitrotriphenylamine
-1,57
-1,67
0,02
0,01
0,00
-0,01
-0,02
-0,03
I / μA
-2,2
-2,0
-1,8
-1,6
-1,4
-1,2
-1,0
E / V

9) UV-Vis data [λ in nm (solvent)]

	-OMe	-H	2-Br	$-NO_2$
TPA	-	370[a] (DCM)	-	-
$TPA^{\cdot+}$	695[b] (ACN)	679[b] (ACN)	-	656[b] (ACN)
$TPB^{\cdot+}$	1574; 492[d] (DCM)	1385; 481[d] (DCM)<br>1200 - 1400; 480[a] (DCM)	1490; 489[c] (DCM)	-
TPB^{2+}	878[d] (DCM)	742[d] (DCM)	807[c] (DCM)	-

[a] S. Maddala, S. Mallik, P. Venkatakrishnan, *J. Org. Chem.* **2017**, *82*, 8958–8972.
[b] K. Sreenath, C. V. Suneesh, K. R. Gopidas, R. A. Flowers II, *J. Phys. Chem. A* **2009**, *113*, 6477–6483.
[c] M. R. Talipov, M. M. Hossain, A. Boddeda, K. Thakur, R. Rathore, *Org. Biomol. Chem.* **2016**, *14*, 2961–2968.
[d] Y. Su, X. Wang, X. Zheng, Z. Zhang, Y. Song, Y. Sui, Y. Li, X. Wang, *Angew. Chem. Int. Ed.* **2014**, *53*, 2857–2861.

Conclusions from the experiments

With a substrate/GFA ratio of 1:1 the reactions proceeded almost quantitatively, without side products under the applied conditions. In the case of a substrate: GFA ratio of 1:0.65 the reactions were slower and substrate was still present when the reaction time was doublet.

The NMR spectra taken for the experiments with only small amounts of HBF_4 either show still the presence of unreacted substrate (for small reaction times) or the products of additional coupling (for longer reaction times). The reaction with 4,4'-dibromtriphenylamine was an exception, since formation of additional coupling products is prohibited by the bromo substituents. In this case the reaction time can be increased without the observation of further products. However, the reaction was much slower and not quantitative.

10) Experimental Details for oxidative coupling of 3,3'',4,4''-tetramethoxy-*o*-terphenyl to 2,3,10,11-tetramethoxy-triphenylene

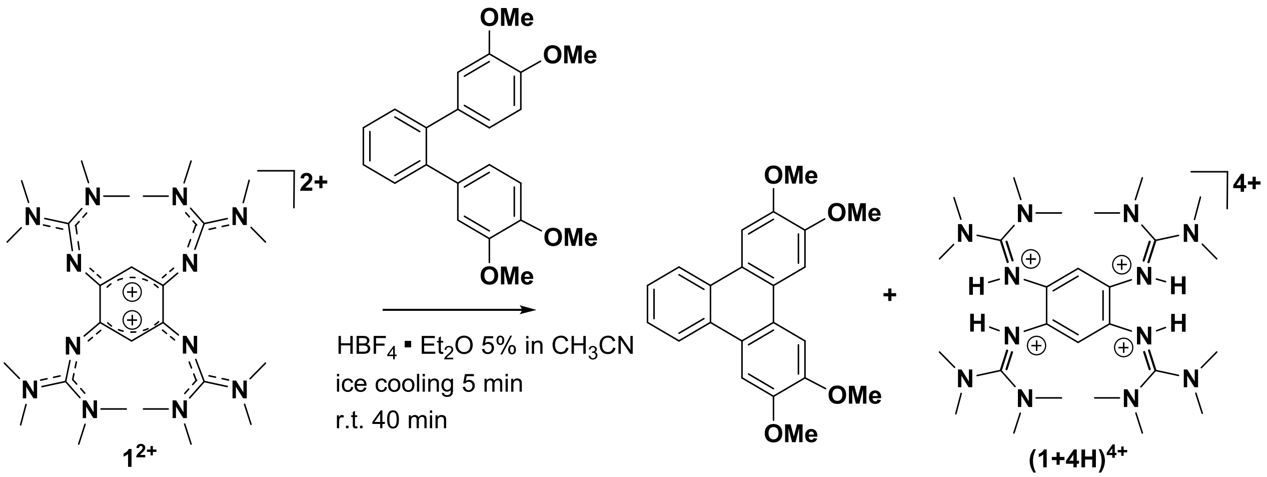

entry	A	B	C
**1**$(BF_4)_2$ [mg/µmol]	6.224/8.84	-	6.256/8.88
**1**$(PF_6)_2$ [mg/µmol]	-	5.424/6.61	-
3,3'',4,4''-tetramethoxy-*o*-terphenyl [mg/µmol]	3.054/8.72	2.286/6.52	3.026/8.64
$HBF_4 \cdot Et_2O$ (5% in CH_3CN) [mL/µmol]	0.5/182.3	0.5/182.3	0.15/54.7
HMB [mg/µmol]	2.160/13.31	1.758/10.83	1.380/8.50
CH_3CN [mL]	-	-	1
reaction time (temperature)	5 min (ice cooling) 40 min (r.t.)	10 min (ice cooling) 40 min (r.t.)	5 h (60 °C)
conversion (rel. to $\mathbf{1}^{2+}$/$(\mathbf{1}+2H)^{2+}$) [%]	99	98	97
ratio (substrate/GFA)	1/1	1/1	1/1

Protocol for entries A & B

In a Schlenk flask, 0.5 mL of a $HBF_4 \cdot Et_2O$ solution (5% in CH_3CN) was added with a syringe under ice cooling in an Ar atmosphere to the reactants **1**$(BF_4)_2$ / **1**$(PF_6)_2$) and 3,3'',4,4''-tetramethoxy-*o*-terphenyl. The reaction was stirred for several minutes under ice cooling and then allowed to warm up to room temperature for further reaction.

Protocol for entry C

In a Schlenk flask, 0.5 mL of a $HBF_4 \cdot Et_2O$ solution (5% in CH_3CN) was added with a syringe in an Ar atmosphere to a solution of the reactants **1**$(BF_4)_2$ and 3,3'',4,4''-tetramethoxy-*o*-terphenyl in 1 mL CH_3CN. The reaction was stirred for 5 h at a temperature of 60 °C.

Work-up entries A - C

The reaction was stopped by the addition of an aqueous saturated $NaHCO_3$ solution. The small amount of CH_3CN was removed under high-vacuum. An aqueous diluted solution of NaOH (8%) was added (pH > 9), converting all formed $(\mathbf{1}+4H)^{4+}$ to $(\mathbf{1}+2H)^{2+}$ (for subsequent transfer into the organic phase). A clear solution was obtained that was extracted several times with CH_2Cl_2. The combined organic phases were dried over Na_2SO_4, filtrated and condensed.

^{1}H NMR spectra (399.89 MHz, 295.5 K, CD_2Cl_2) for entry A

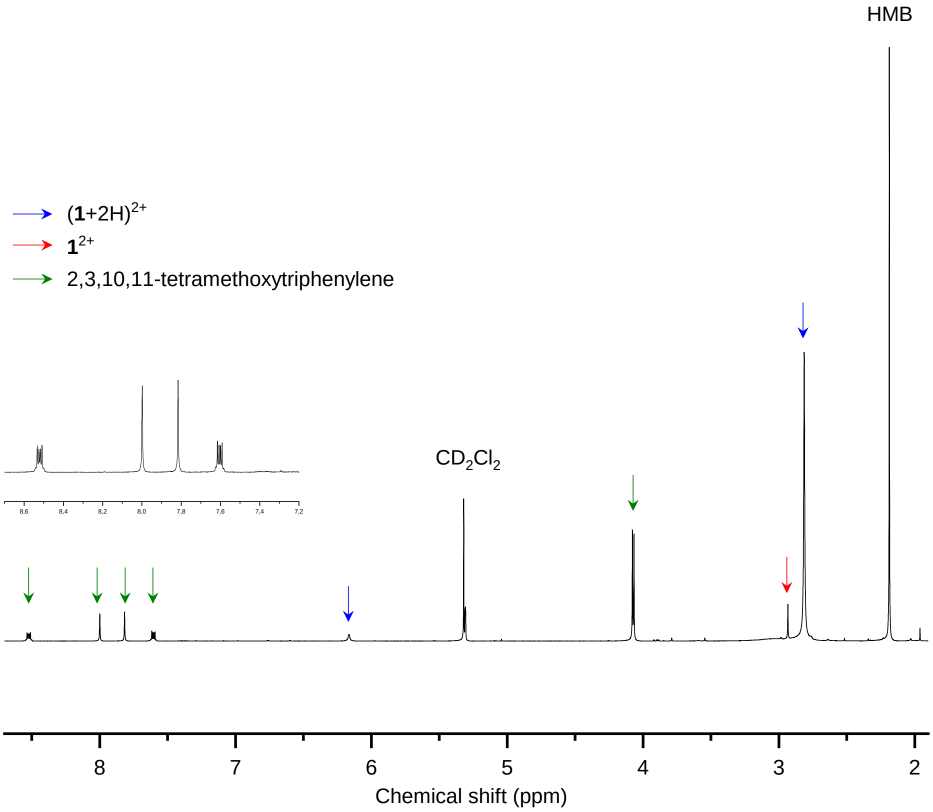

^{1}H NMR of 2,3,10,11 - tetramethoxytriphenylen: (399.89 MHz, 295.5 K, CD_2Cl_2): δ = 4.07 (*d*, *J* = 4.4 Hz, 12 H), 7.60 (dd, *J* = 6.3; 3.3 Hz, 2 H), 7.82 (s, 2 H), 8.00 (s, 2 H), 8.52 (dd, *J* = 6.3; 3.4 Hz, 2 H) ppm.

Protocol for additional NMR experiments

5.256 mg (7.46 µmol) of **1**$(BF_4)_2$, 0.810 mg (4.99 µmol) HMB and 2.474 mg (7.06 µmol) of 3,3'',4,4''-tetramethoxy-*o*-terphenyl were dissolved in an NMR tube under an Ar atmosphere in 0.45 mL CD_3CN. Then 0.05 mL (18.23 µmol) of a $HBF_4{\cdot}Et_2O$ solution (5% in CD_3CN) were added with a syringe. The reaction mixture was heated to a temperature of 60 °C.

^{1}H NMR experiment (199.87 MHz, 298 K, CD_3CN): reaction of 1$(BF_4)_2$ and 3,3'',4,4''-tetramethoxy-*o*-terphenyl without addition of $HBF_4{\cdot}Et_2O$ solution (5% in CD_3CN)

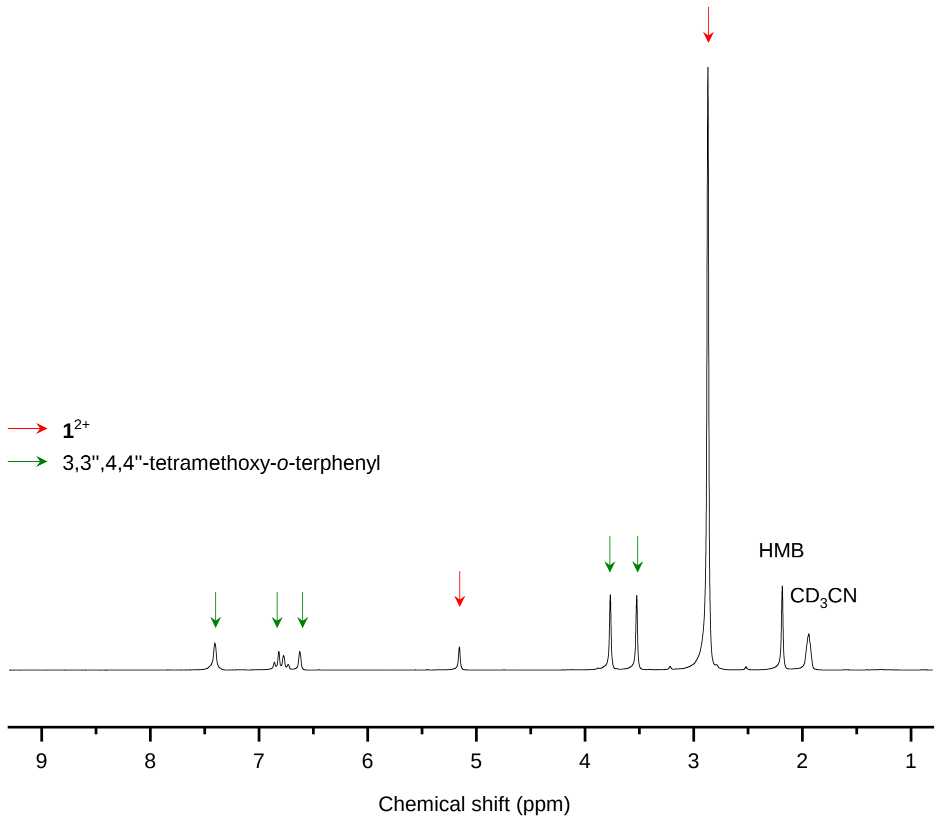

^{1}H NMR experiment (199.87 MHz, 298 K, CD_3CN): reaction of $1(BF_4)_2$ and 3,3'',4,4''-tetramethoxy-*o*-terphenyl after the addition of 0.05 mL $HBF_4{\cdot}Et_2O$ solution (5% in CD_3CN)

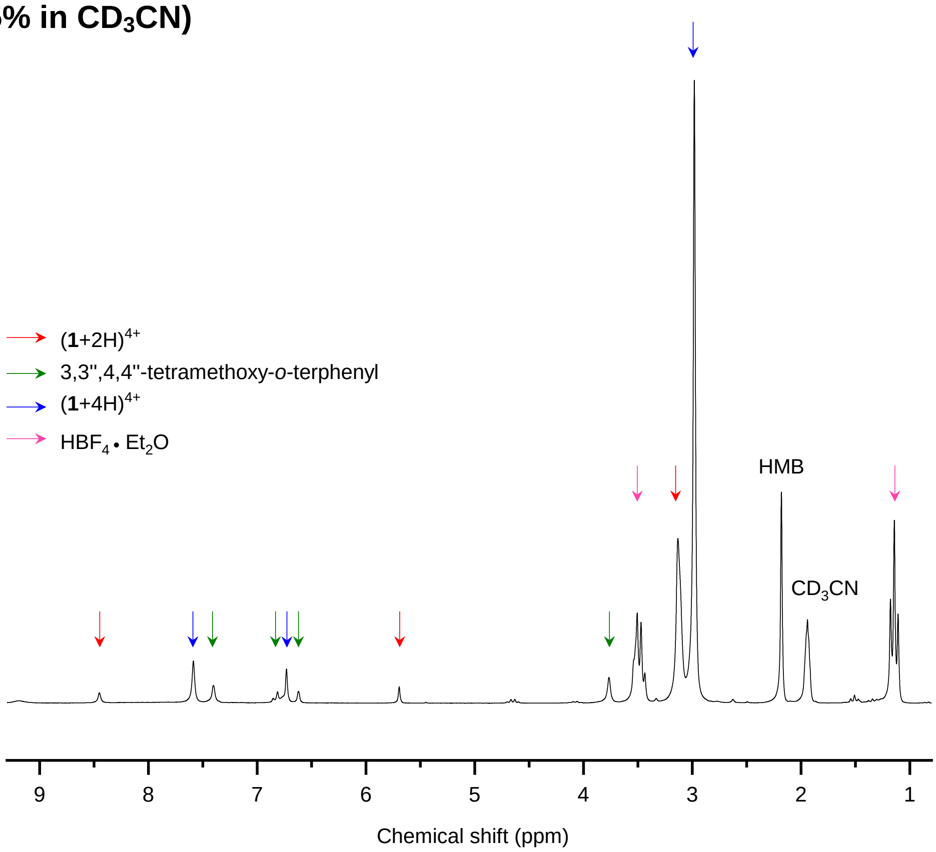

^{1}H NMR experiment (199.87 MHz, 298 K, CD_3CN): reaction of $1(BF_4)_2$ and 3,3'',4,4''-tetramethoxy-*o*-terphenyl 1 h after the addition of 0.05 mL $HBF_4{\cdot}Et_2O$ solution (5% in CD_3CN)

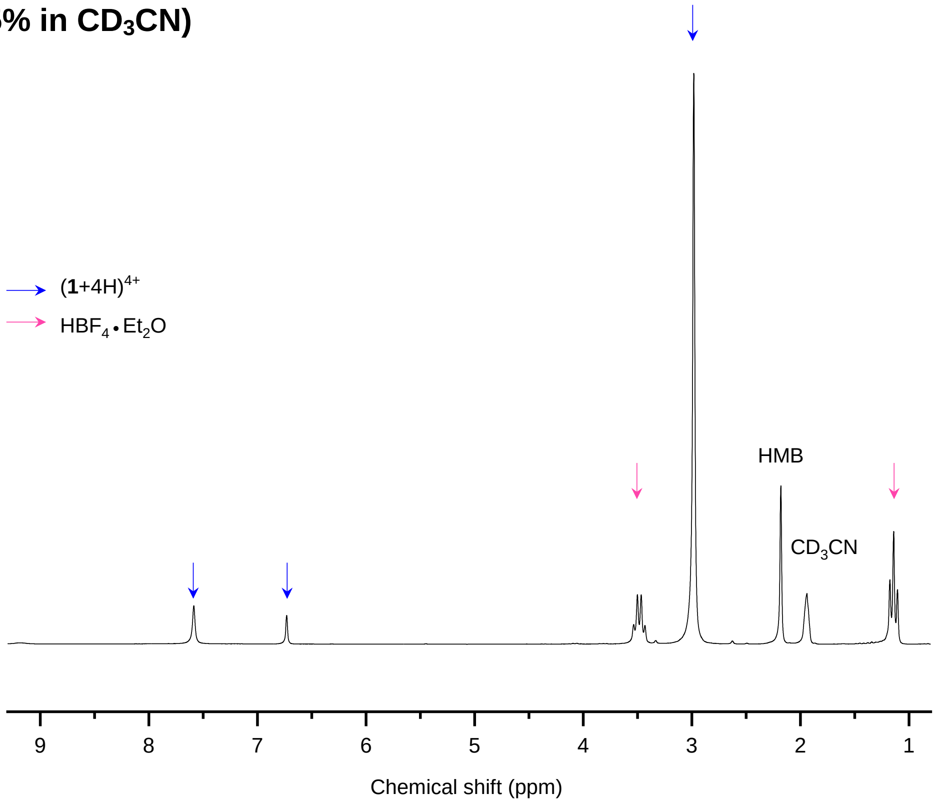

11) Recycling experiments

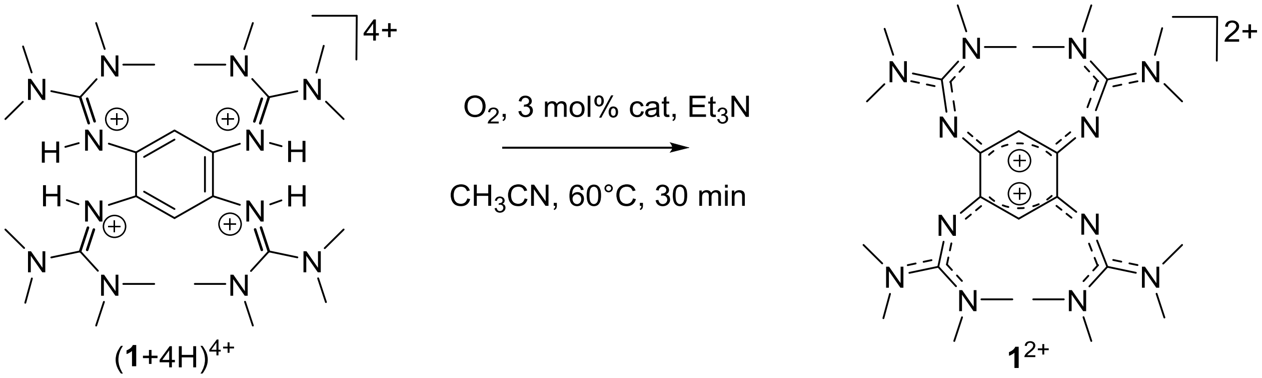

entry	A	B	C
(**1**+4H)$(BF_4)_4$ [mg/µmol]	33.03/37.45	8.81/9.99	4.278/4.85
$CuCl_2$ [µmol/mol%]	1.13/3.0	0.30/3.0	0.15/3.1
$[Cu(H_2O)_6(BF_4)_2]$ [µmol/mol%]	1.11/3.0	0.29/3.0	0.14/2.9
NEt_3 [µL/µmol]	15/107.6	4/28.7	2/14.4
Na_2SO_4 [mg]	2.446	1.566	1.900
CD_3CN [mL]	0.6	0.6	0.6
reaction time (temperature)	0.5 h (60 °C)	0.5 h (60 °C)	0.5 h (60 °C)
c (mmol·l^{-1})	62	17	8
conversion [%]	>99	>99	>99

First stock solutions of the copper compounds ($CuCl_2$ and $[Cu(H_2O)_6(BF_4)_2]$) in CH_3CN were prepared. Then the desired amount of catalyst was introduced with a syringe into a Schlenk flask. The solvent was removed under vacuum, and the solid catalyst (cat = equimolar mixture of $CuCl_2$ and $[Cu(H_2O)_6(BF_4)_2]$) re-dissolved in 0.6 mL CD_3CN.

In a vial, the catalyst solution and NEt_3 was added under an atmosphere of dioxygen to (**1**+4H)$(BF_4)_4$ and Na_2SO_4. The vial was locked with a septum and the reaction mixture stirred under 0.2 bar of dioxygen for a period of 30 min at 60 °C. The conversion was followed by NMR spectroscopy. The reaction was stopped by the addition of a saturated $NaHCO_3$ solution. After solvent removal the residue was collected with H_2O and CH_2Cl_2. The organic phases were separated and the aqueous phase extracted several times with CH_2Cl_2. The combined organic phases were dried over Na_2SO_4.

^1H NMR spectra (199.87 MHz, 298 K, CD_3CN) for entry B

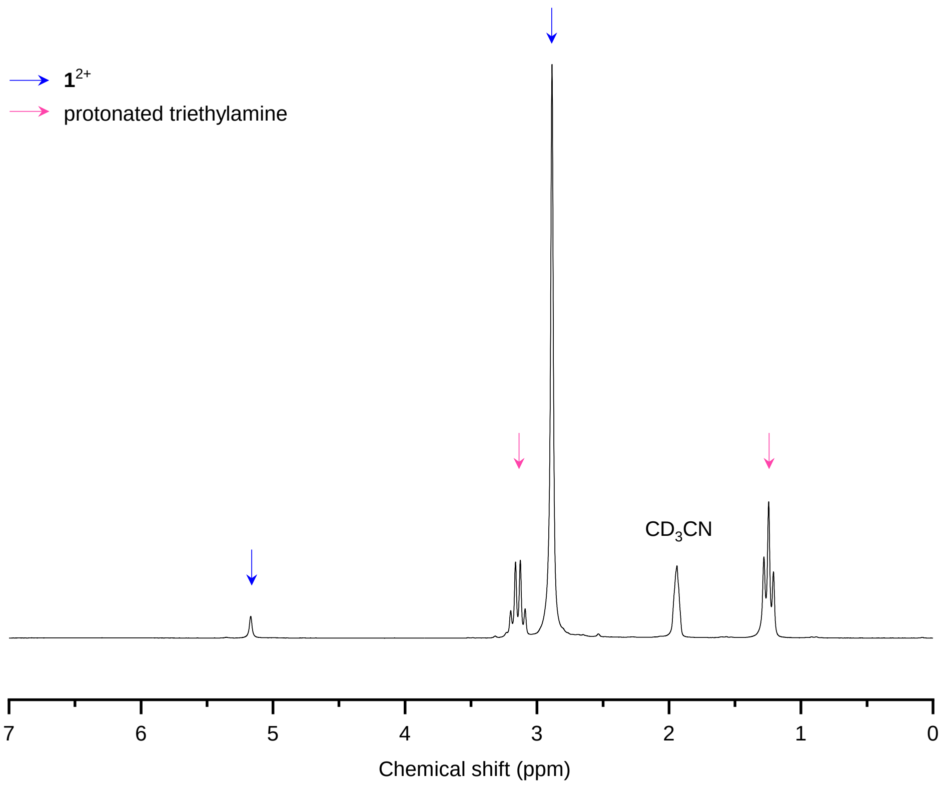

^1H NMR spectra (199.87 MHz, 298 K, CD_3CN) of combined entries A - C after work - up

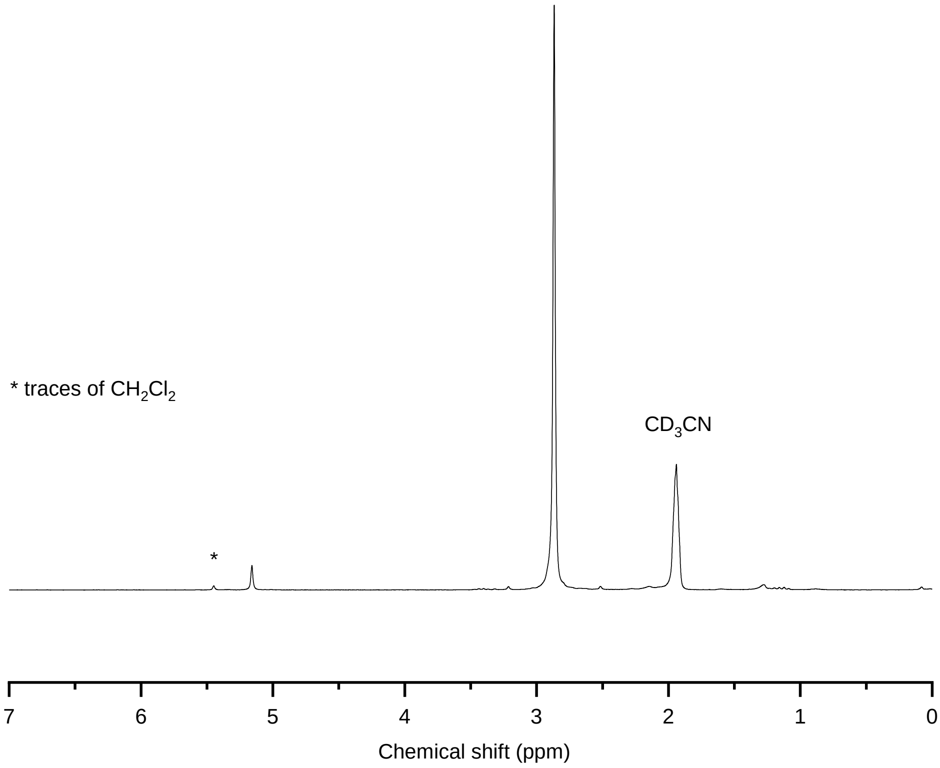

^1H NMR of **1**$(BF_4)_2$: (199.87 MHz, 298 K, CD_3CN): δ = 2.87 (s, 48 H), 5.16 (s, 2 H) ppm.

Synthesis of (1+4H)(BF_4)$_4$

In a Schenk flask, 1.15 mL (419.18 µmol) of a $HBF_4 \cdot Et_2O$ solution (5% in CH_3CN) were dropwise added with a syringe to a suspension of 55 mg (103.62 µmol) of **1** in 4 mL CH_3CN. The reaction mixture was stirred for 1 h at room temperature. The solvent was removed under vacuum and the colourless residue washed several times with diethylether. Yield: 72 mg (81.63 µmol, 79 %). $C_{26}H_{54}N_{12}B_4F_{16}$ (882.01 g mol^{-1}): calcd. C 35.41, H 6.17, N 19.06; found C 35.05, H 5.91, N 19.03. ^{1}H NMR (399.87 MHz, CD_3CN, 295.7 K): δ = 2.98 (s, 48 H, C*H$_3$*), 6.72 (s, 2 H, C*H*), 7.59 (s, 4 H, N*H*) ppm. ^{11}B-NMR (128.30 MHz, CD_3CN, 295.7 K): δ = -1.17 ppm. ^{13}C NMR (100.56 MHz, CD_3CN, 296.7 K): δ = 41.09 (*C*H$_3$), 118.81 ($C_{arom.}$), 129.96, 159.44 (C_g) ppm. MS (ESI, CH_3CN): m/z (%) = 619 (100) [(**1**+2H)(BF_4)]$^+$, 531 (70) [**1**]$^+$, 707 (52) [(**1**+3H)(BF_4)$_2$]$^+$, 795 (10) [(**1**+4H)(BF_4)$_3$]$^+$, 341 (36). IR (CsI): ṽ = 3319 (m), 2961 (m), 1734 (sh), 1636 (s), 1549 (s), 1497 (sh), 1457 (m), 1447 (m), 1422 (s), 1403 (s), 1305 (m), 1262 (w), 1240 (w), 1193 (w), 1169 (m), 1145 (sh), 1076 (sh), 1058 (vs), 1040 (sh), 897 (w), 881 (w), 800 (w), 720 (w), 676 (w), 648 (w),599 (w), 522 (m), 473 (w) cm^{-1}. UV-Vis (CH_3CN, c = 5.60·10^{-5} M, d = 1 cm): λ (ε in L mol^{-1} cm^{-1}) = 316 (sh, 7850), 284 (23630), 256 (sh, 33030), 242 (40430) nm.

^{1}H NMR spectra (399.89 MHz, 295.7 K, CD_3CN) of (1+4H)(BF_4)$_4$

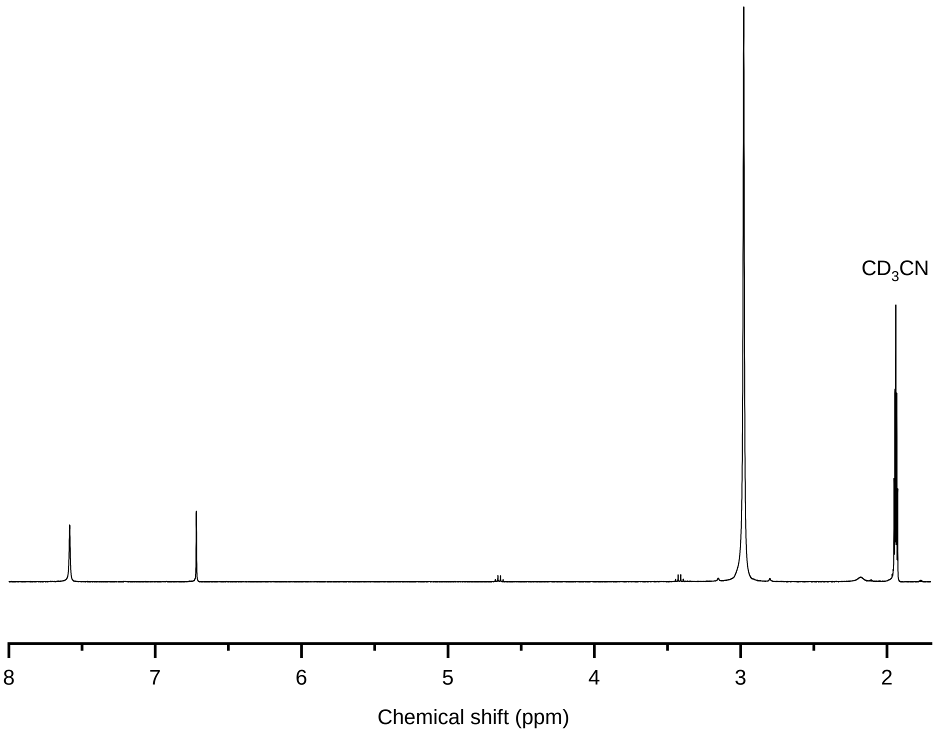

12) Quantum chemical calculations

Details of the quantum-chemical calculations

Quantum-chemical calculations were carried out on the compounds **1**$^{2+}$, (**1**+2H)$^{2+}$, **1**(BF_4)$_2$, (**1**+2H)(BF_4)$_2$, $C_6Cl_4(OH)_2$, $C_6Cl_4O_2$ (**CA**), $C_6H_4(OH)_2$, $C_6H_4O_2$ (**BQ**), $C_6Cl_2(CN)_2(OH)_2$, $C_6Cl_2(CN)_2O_2$ (**DDQ**), (**1**+2H)$^{4+}$, (**1**+4H)$^{4+}$, (**1**+2H)(BF_4)$_4$, (**1**+4H)(BF_4)$_4$, $(C_6Cl_4O_2+2H)^{2+}$, $C_6Cl_4O_2(HF)_2(BF_3)_2$ (result of the attempt to calculate the twofold protonated **CA** with two BF_4^- counter-ions), $(C_6H_4O_2+2H)^{2+}$, $C_6H_4O_2(HF)_2(BF_3)_2$, (**1**+H)$^{3+}$, $(C_6Cl_4O_2+H)^+$, and BF_4^-. These calculations relied on the TURBOMOLE program.[1,2,3] The B3LYP functional [4,5] was used in combination with the def2-SV(P) or def2-TZVP basis set.[6] The influence of a polarizable environment was considered by the conductor-like screening model (COSMO).[7]

**Compound**	***E* (TZVP) / hartree**	***E* (SV(P) / hartree**	**ZPVE (SV(P) / kJ mol^{-1}**	**μ_{298} / kJ mol^{-1}**
**1**$^{2+}$	-1676.926440	-1674.983327	0.7725901	1807.58
(**1**+2H)$^{2+}$	-1678.160534	-1676.213037	0.7973611	1882.61
**1**(BF_4)$_2$	-2526.372388	-2523.389345	0.8071689	1867.12
(**1**+2H)(BF_4)$_2$	-2527.606309	-2524.616407	0.8314375	1933.59
$C_6Cl_4(OH)_2$	-2220.868853	-2219.781158	0.0703544	85.67
$C_6Cl_4O_2$ (**CA**)	-2219.631177	-2218.563340	0.0471586	24.58
$C_6H_4(OH)_2$	-382.630902	-382.168603	0.1082916	205.94
$C_6H_4O_2$ (**BQ**)	-381.397659	-380.955550	0.0852803	147.24
$C_6Cl_2(CN)_2(OH)_2$	-1486.204688	-1485.227188	0.0866643	125.64
$C_6Cl_2(CN)_2O_2$ (**DDQ**)	-1484.953539	-1483.996510	0.0631770	62.13
(**1**+2H)$^{4+}$	-1677.301599	-1675.348289	0.7996077	1886.48
(**1**+4H)$^{4+}$	-1678.549891	-1676.591423	0.8241836	1955.26
(**1**+2H)(BF_4)$_4$	-3376.623575	-3372.606495	0.8666310	1984.75
(**1**+4H)(BF_4)$_4$	-3377.888141	-3373.865924	0.8919710	2054.67
$(C_6Cl_4O_2+2H)^{2+}$	-2220.102422	-2219.017931	0.0699483	82.21
$C_6Cl_4O_2(HF)_2(BF_3)_2$	-3069.758291	-3067.620892	0.0984588	92.34
$(C_6H_4O_2+2H)^{2+}$	-381.863637	-381.404077	0.1086933	206.16
$C_6H_4O_2(HF)_2(BF_3)_2$	-1231.535578	-1230.028537	0.1371169	221.67
(**1**+H)$^{3+}$	-1677.166120	-1675.218750	0.7867587	1851.15
$(C_6Cl_4O_2+H)^+$	-2219.945117	-2218.871082	0.0589688	52.32
BF_4^-	-424.575347	-424.036813	0.0146443	-27.92

Reaction energies, enthalpies (at 0 K) and Gibbs free energies (at 298 K)

$C_6Cl_4(OH)_2$ + $\mathbf{1}^{2+}$ → $C_6Cl_4O_2$ + $(\mathbf{1}+2H)^{2+}$

ΔE_{el} (B3LYP/TZVP) = 9.4045 kJ/mol = 0.0975 eV
ΔE_{el} (B3LYP/SV(P)) = -31.2224 kJ/mol = -0.3236 eV
ΔH (0 K) (B3LYP/TZVP/SV(P)) = 13.5402 kJ/mol = 0.1404 eV
ΔG_{298}(B3LYP/TZVP/SV(P)) = 23.34 kJ/mol = 0.2420 eV

ΔE_{vib}(0 K) (B3LYP/SV(P)) = 4.1357 kJ/mol = 0.0429 eV
$\Delta\Delta G_{298}$(B3LYP/SV(P)) = 13.94 kJ/mol = 0.1445 eV

$C_6Cl_4(OH)_2$ + **1**$(BF_4)_2$ → $C_6Cl_4O_2$ + (**1**+2H)$(BF_4)_2$

ΔE_{el} (B3LYP/TZVP) = 9.8588 kJ/mol = 0.1022 eV
ΔE_{el} (B3LYP/SV(P)) = -24.2701 kJ/mol = -0.2515 eV
ΔH (0 K) (B3LYP/TZVP/SV(P)) = 12.6754 kJ/mol = 0.1314 eV
ΔG_{298}(B3LYP/TZVP/SV(P)) = 5.24 kJ/mol = 0.1580 eV

ΔE_{vib}(0 K) (B3LYP/SV(P)) = 2.8166 kJ/mol = 0.0292 eV
$\Delta\Delta G_{298}$(B3LYP/SV(P)) = 5.38 kJ/mol = 0.0558 eV

$C_6H_4(OH)_2$ + **1**$(BF_4)_2$ → $C_6H_4O_2$ + (**1**+2H)$(BF_4)_2$

ΔE_{el} (B3LYP/TZVP) = -1.7801 kJ/mol = -0.0184 eV
ΔE_{el} (B3LYP/SV(P)) = -36.7806 kJ/mol = -0.3812 eV
ΔH (0 K) (B3LYP/TZVP/SV(P)) = 1.5209 kJ/mol = 0.0158 eV
ΔG_{298}(B3L/TZVP/SV(P)) = 5.99 kJ/mol = 0.0621 eV

ΔE_{vib}(0 K) (B3LYP/SV(P)) = 3.3010 kJ/mol = 0.0342 eV
$\Delta\Delta G_{298}$(B3LYP/SV(P)) = 7.77 kJ/mol = 0.0805 eV

$C_6Cl_2(CN)_2(OH)_2$ + **1**$(BF_4)_2$ → $C_6Cl_2(CN)_2O_2$ + (**1**+2H)$(BF_4)_2$

ΔE_{el} (B3LYP/TZVP) = 45.2321 kJ/mol = 0.4688 eV
ΔE_{el} (B3LYP/SV(P)) = 9.4938 kJ/mol = 0.0984 eV
ΔH (0 K) (B3LYP/TZVP/SV(P)) = 47.2834 kJ/mol = 0.4901 eV
ΔG_{298}(B3LYP/TZVP/SV(P)) = 48.19 kJ/mol = 0.4995 eV

ΔE_{vib}(0 K) (B3LYP/SV(P)) = 2.0513 kJ/mol = 0.0213 eV
$\Delta\Delta G_{298}$(B3LYP/SV(P)) = 2.96 kJ/mol = 0.0307 eV

$C_6Cl_4(OH)_2$ + (**1**+2H)$^{4+}$ → $C_6Cl_4O_2$ + (**1**+4H)$^{4+}$

ΔE_{el} (B3LYP/TZVP) = -27.8723 kJ/mol = -0.2889 eV
ΔE_{el} (B3LYP/SV(P)) = -66.4671 kJ/mol = -0.6889 eV
ΔH (0 K) (B3LYP/TZVP/SV(P)) = -24.2488 kJ/mol = -0.2513 eV
ΔG_{298}(B3LYP/TZVP/SV(P)) = -20.18 kJ/mol = -0.2092 eV

ΔE_{vib}(0 K) (B3LYP/SV(P)) = 3.6235 kJ/mol = 0.0376 eV
$\Delta\Delta G_{298}$(B3LYP/SV(P)) = 7.69 kJ/mol = 0.0797 eV

$C_6Cl_4(OH)_2$ + (**1**+2H)$(BF_4)_4$ → $C_6Cl_4O_2$ + (**1**+4H)$(BF_4)_4$

ΔE_{el} (B3LYP/TZVP) = -70.5997 kJ/mol = -0.7317 eV
ΔE_{el} (B3LYP/SV(P)) = -109.2497 kJ/mol = -1.1323 eV
ΔH (0 K) (B3LYP/TZVP/SV(P)) = -64.9701 kJ/mol = -0.6734 eV
ΔG_{298}(B3L/TZVP/SV(P)) = -61.77 kJ/mol = -0.6402 eV

ΔE_{vib}(0 K) (B3LYP/SV(P)) = 5.6296 kJ/mol = 0.0583 eV
$\Delta\Delta G_{298}$(B3LYP/SV(P)) = 8.83 kJ/mol = 0.0915 eV

$C_6Cl_4O_2$ + (**1**+H)$^{3+}$ → $(C_6Cl_4O_2+H)^+$ + **1**$^{2+}$

ΔE_{el} (B3LYP/TZVP) = -194.9696 kJ/mol = -2.0207 eV
ΔE_{el} (B3LYP/SV(P)) = -189.8735 kJ/mol = -1.9679 eV
ΔH (0 K) (B3LYP/TZVP/SV(P)) = -201.1616 kJ/mol = -2.0849 eV
ΔG_{298}(B3LYP/TZVP/SV(P)) = -210.80 kJ/mol = -2.1848 eV

ΔE_{vib}(0 K) (B3LYP/SV(P)) = -6.1920 kJ/mol = -0.0642 eV
$\Delta\Delta G_{298}$(B3LYP/SV(P)) = -15.83 kJ/mol = -0.1641 eV

$C_6Cl_4O_2$ + (**1**+2H)$^{4+}$ → $(C_6Cl_4O_2+2H)^{2+}$ + **1**$^{2+}$

ΔE_{el} (B3LYP/TZVP) = -252.2738 kJ/mol = -2.6146 eV
ΔE_{el} (B3LYP/SV(P)) = -235.3209 kJ/mol = -2.4389 eV
ΔH (0 K) (B3LYP/TZVP/SV(P)) = -263.3741 kJ/mol = -2.7296 eV
ΔG_{298}(B3LYP/TZVP/SV(P)) = -273.54 kJ/mol = -2.8350 eV

ΔE_{vib}(0 K) (B3LYP/SV(P)) = -11.1003 kJ/mol = -0.1150 eV
$\Delta\Delta G_{298}$(B3LYP/SV(P)) = -21.27 kJ/mol = -0.2204 eV

$C_6Cl_4O_2$ + (**1**+2H)$(BF_4)_4$ → $C_6Cl_4O_2(HF)_2(BF_3)_2$ + **1**$(BF_4)_2$

ΔE_{el} (B3LYP/TZVP) = 325.7536 kJ/mol = 3.3762 eV
ΔE_{el} (B3LYP/SV(P)) = 419.0245 kJ/mol = 4.3429 eV
ΔH (0 K) (B3LYP/TZVP/SV(P)) = 304.3245 kJ/mol = 3.1541 eV
ΔG_{298}(B3LYP/TZVP/SV(P)) = 275.88 kJ/mol = 2.8593 eV

ΔE_{vib}(0 K) (B3LYP/SV(P)) = -21.4291 kJ/mol = -0.2221 eV
$\Delta\Delta G_{298}$(B3LYP/SV(P)) = -49.87 kJ/mol = -0.5169 eV

$C_6H_4O_2$ + $(\mathbf{1}+2H)^{4+}$ → $(C_6H_4O_2+2H)^{2+}$ + $\mathbf{1}^{2+}$

ΔE_{el} (B3LYP/TZVP) = -238.4453 kJ/mol = -2.4713 eV
ΔE_{el} (B3LYP/SV(P)) = -219.3999 kJ/mol = -2.2739 eV
ΔH (0 K) (B3LYP/TZVP/SV(P)) = -247.9092 kJ/mol = -2.5694 eV
ΔG_{298}(B3L/TZVP/SV(P)) = -258.43 kJ/mol = -2.6784 eV

ΔE_{vib}(0 K) (B3LYP/SV(P)) = -9.4639 kJ/mol = -0.0981 eV
$\Delta\Delta G_{298}$(B3LYP/SV(P)) = -19.98 kJ/mol = -0.2071 eV

$C_6H_4O_2$ + (**1**+2H)$(BF_4)_4$ → $C_6H_4O_2(HF)_2(BF_3)_2$ + **1**$(BF_4)_2$

ΔE_{el} (B3LYP/TZVP) = 297.3851 kJ/mol = 3.0822 eV
ΔE_{el} (B3LYP/SV(P)) = 378.4999 kJ/mol = 3.9229 eV
ΔH (0 K) (B3LYP/TZVP/SV(P)) = 277.4789 kJ/mol = 2.8747 eV
ΔG_{298}(B3L/TZVP/SV(P)) = 254.30 kJ/mol = 2.6345 eV

ΔE_{vib}(0 K) (B3LYP/SV(P)) = -20.0207 kJ/mol = -0.2075 eV
$\Delta\Delta G_{298}$(B3LYP/SV(P)) = -43.20 kJ/mol = -0.4477 eV

$C_6H_4(OH)_2$ + (**1**+2H)$(BF_4)_4$ → $C_6H_4O_2$ + ttmgb$H_4(BF_4)_4$

ΔE_{el}(B3LYP/TZVP) = -82.2385 kJ/mol = -0.8523 eV
ΔE_{el}(B3LYP/SV(P)) = -121.7602 kJ/mol = -1.2620 eV
ΔH (0 K) (B3LYP/TZVP/SV(P)) = -76.1245 kJ/mol = -0.7889 eV
ΔG_{298}(B3LYP/TZVP/SV(P)) = -71.02 kJ/mol = -0.7360 eV

ΔE_{vib}(0 K) (B3LYP/SV(P)) = 6.1140 kJ/mol = 0.0634 eV
$\Delta\Delta G_{298}$(B3LYP/SV(P)) = 11.22 kJ/mol = 0.1163 eV

$C_6Cl_2(CN)_2(OH)_2$ + (**1**+2H)$(BF_4)_4$ → $C_6Cl_2(CN)_2O_2$ + ttmgb$H_4(BF_4)_4$

ΔE_{el}(B3LYP/TZVP) = -35.2263 kJ/mol = -0.3651 eV

ΔE_{el}(B3LYP/SV(P)) = -75.4857 kJ/mol = -0.7824 eV
ΔH (0 K) (B3LYP/TZVP/SV(P)) = -30.3620 kJ/mol = -0.3147 eV
ΔG_{298}(B3LYP/TZVP/SV(P)) = -28.82 kJ/mol = -0.2987 eV

ΔE_{vib} (0 K) (B3LYP/SV(P)) = 4.8643 kJ/mol = 0.0504 eV
$\Delta\Delta G_{298}$(B3LYP/SV(P)) = 6.41 kJ/mol = 0.0664 eV

Calculations with COSMO for varying relative permittivities ε_r

Calculations for the reaction $C_6H_4(OH)_2$ + $\mathbf{1}^{2+}$ → $C_6H_4O_2$ + $(\mathbf{1}+2H)^{2+}$

E(B3LYP/TZVP) / hartree for the structures optimized at ε_r =1

ε_r	$\mathbf{1}^{2+}$	$(\mathbf{1}+2H)^{2+}$	$C_6H_4(OH)_2$	$C_6H_4O_2$
1	-1676.926440	-1678.160534	-382.630902	-381.397659
40	-1677.107337	-1678.342689	-382.646936	-381.410799

ΔE_{el} (B3LYP/TZVP, ε_r = 1) = -2.2343 kJ/mol = -0.0232 eV
ΔE_{el} (B3LYP/TZVP, ε_r = 40) = 2.0610 kJ/mol = 0.0214 eV

Calculations for the reaction $C_6H_4(OH)_2$ + $\mathbf{1}(BF_4)_2$ → $C_6H_4O_2$ + $(\mathbf{1}+2H)(BF_4)_2$

E(B3LYP/TZVP) / hartree for the structures optimized at ε_r = 1

ε_r	$\mathbf{1}(BF_4)_2$	$(\mathbf{1}+2H)(BF_4)_2$	$C_6H_4(OH)_2$	$C_6H_4O_2$
1	-2526.372388	-2527.606309	-382.630902	-381.397659
40	-2526.439210	-2527.670528	-382.646936	-381.410799

ΔE_{el} (B3LYP/TZVP, ε_r = 1) = -1.7801 kJ/mol = -0.0184 eV
ΔE_{el} (B3LYP/TZVP, ε_r = 40) = 12.6523 kJ/mol = 0.1311 eV

E(B3LYP/TZVP) / hartree for the structures optimized at each value of ε_r

ε_r	$\mathbf{1}(BF_4)_2$	$(\mathbf{1}+2H)(BF_4)_2$	$C_6H_4(OH)_2$	$C_6H_4O_2$
1	-2526.372388	-2527.606309	-382.630902	-381.397659
40	-2526.451653	-2527.683936	-382.647011	-381.410950

ΔE_{el} (B3LYP/TZVP, ε_r = 1) = -1.7801 kJ/mol = -0.0184 eV
ΔE_{el} (B3LYP/TZVP, ε_r = 40) = 9.9191 kJ/mol = 0.1028 eV

Calculations for the reaction $C_6Cl_4(OH)_2$ + $\mathbf{1}^{2+}$ → $C_6Cl_4O_2$ + $(\mathbf{1}+2H)^{2+}$

E(B3LYP/TZVP) / hartree for the structures optimized at ε_r =1

ε_r	$\mathbf{1}^{2+}$	$(\mathbf{1}+2H)^{2+}$	$C_6Cl_4(OH)_2$	$C_6Cl_4O_2$
1	-1676.926440	-1678.160534	-2220.868853	-2219.631177
2	-1677.001172	-1678.235832	-2220.872857	-2219.635256
5	-1677.062719	-1678.297811	-2220.876410	-2219.639026
10	-1677.087270	-1678.322513	-2220.877900	-2219.640651
20	-1677.100478	-1678.335795	-2220.878720	-2219.641557
40	-1677.107337	-1678.342689	-2220.879151	-2219.642037
60	-1677.109663	-1678.345027	-2220.879298	-2219.642201
80	-1677.110834	-1678.346203	-2220.879372	-2219.642284

ΔE_{el} (B3LYP/TZVP, ε_r = 1) = 9.4045 kJ/mol = 0.0975 eV
ΔE_{el} (B3LYP/TZVP, ε_r = 2) = 7.7216 kJ/mol = 0.0800 eV
ΔE_{el} (B3LYP/TZVP, ε_r = 5) = 6.0176 kJ/mol = 0.0624 eV

ΔE_{el} (B3LYP/TZVP, ε_r = 10) = 5.2668 kJ/mol = 0.0546 eV
ΔE_{el} (B3LYP/TZVP, ε_r = 20) = 4.8467 kJ/mol = 0.0502 eV
ΔE_{el} (B3LYP/TZVP, ε_r = 40) = 4.6261 kJ/mol = 0.0479 eV
ΔE_{el} (B3LYP/TZVP, ε_r = 60) = 4.5500 kJ/mol = 0.0472 eV
ΔE_{el} (B3LYP/TZVP, ε_r = 80) = 4.5132 kJ/mol = 0.0468 eV

Calculations for the reaction $C_6Cl_4(OH)_2$ + **1**$(BF_4)_2$ → $C_6Cl_4O_2$ + (**1**+2H)$(BF_4)_2$

E(B3LYP/TZVP) / hartree for the structures optimized at ε_r = 1

ε_r	**1**$(BF_4)_2$	(**1**+2H)$(BF_4)_2$	$C_6Cl_4(OH)_2$	$C_6Cl_4O_2$
1	-2526.372388	-2527.606309	-2220.868853	-2219.631177
2	-2526.398034	-2527.630933	-2220.872857	-2219.635256
5	-2526.421144	-2527.653144	-2220.876410	-2219.639026
10	-2526.430937	-2527.662565	-2220.877900	-2219.640651
20	-2526.436354	-2527.667778	-2220.878720	-2219.641557
40	-2526.439210	-2527.670528	-2220.879151	-2219.642037
80	-2526.440677	-2527.671941	-2220.879372	-2219.642284

ΔE_{el} (B3LYP/TZVP, ε_r = 1) = 9.8587 kJ/mol = 0.1022 eV
ΔE_{el} (B3LYP/TZVP, ε_r = 2) = 12.3451 kJ/mol = 0.1279 eV
ΔE_{el} (B3LYP/TZVP, ε_r = 5) = 14.1357 kJ/mol = 0.1465 eV
ΔE_{el} (B3LYP/TZVP, ε_r = 10) = 14.7579 kJ/mol = 0.1530 eV
ΔE_{el} (B3LYP/TZVP, ε_r = 20) = 15.0677 kJ/mol = 0.1562 eV
ΔE_{el} (B3LYP/TZVP, ε_r = 40) = 15.2174 kJ/mol = 0.1577 eV
ΔE_{el} (B3LYP/TZVP, ε_r = 80) = 15.2909 kJ/mol = 0.1585 eV

E(B3LYP/TZVP) / hartree for the structures optimized at each value of ε_r

ε_r	**1**$(BF_4)_2$	(**1**+2H)$(BF_4)_2$	$C_6Cl_4(OH)_2$	$C_6Cl_4O_2$
1	-2526.372388	-2527.606309	-2220.868853	-2219.631177
2	-2526.399565	-2527.632368	-2220.872880	-2219.635264
5	-2526.427065	-2527.658505	-2220.876498	-2219.639056
10	-2526.440028	-2527.671653	-2220.878020	-2219.640696
20	-2526.447588	-2527.679669	-2220.878864	-2219.641611
40	-2526.451653	-2527.683936	-2220.879308	-2219.642096
80	-2526.453748	-2527.686202	-2220.879540	-2219.642346

ΔE_{el} (B3LYP/TZVP, ε_r = 1) = 9.8587 kJ/mol = 0.1022 eV
ΔE_{el} (B3LYP/TZVP, ε_r = 2) = 12.6365 kJ/mol = 0.1310 eV
ΔE_{el} (B3LYP/TZVP, ε_r = 5) = 15.7582 kJ/mol = 0.1633 eV
ΔE_{el} (B3LYP/TZVP, ε_r = 10) = 14.9627 kJ/mol = 0.1551 eV
ΔE_{el} (B3LYP/TZVP, ε_r = 20) = 13.5791 kJ/mol = 0.1407 eV
ΔE_{el} (B3LYP/TZVP, ε_r = 40) = 12.9411 kJ/mol = 0.1341 eV
ΔE_{el} (B3LYP/TZVP, ε_r = 80) = 12.4449 kJ/mol = 0.1290 eV

Calculations for the reaction $C_6Cl_2(CN)_2(OH)_2$ + **1**$^{2+}$ → $C_6Cl_2(CN)_2O_2$ + (**1**+2H)$^{2+}$

E(B3LYP/TZVP) / hartree for the structures optimized at ε_r =1

ε_r	**1**$^{2+}$	(**1**+2H)$^{2+}$	$C_6Cl_2(CN)_2(OH)_2$	$C_6Cl_2(CN)_2O_2$
1	-1676.926440	-1678.160534	-1486.204688	-1484.953539
40	-1677.107337	-1678.342689	-1486.228322	-1484.973404

ΔE_{el} (B3LYP/TZVP, ε_r = 1) = 44.7779 kJ/mol = 0.4641 eV
ΔE_{el} (B3LYP/TZVP, ε_r = 40) = 51.3705 kJ/mol = 0.5324 eV

Calculations for the reaction $C_6Cl_2(CN)_2(OH)_2$ + **1**$(BF_4)_2$ → $C_6Cl_2(CN)_2O_2$ + (**1**+2H)$(BF_4)_2$

E(B3LYP/TZVP) / hartree for the structures optimized at ε_r = 1

ε_r	**1**$(BF_4)_2$	(**1**+2H)$(BF_4)_2$	$C_6Cl_2(CN)_2(OH)_2$	$C_6Cl_2(CN)_2O_2$
1	-2526.372388	-2527.606309	-1486.204688	-1484.953539
40	-2526.439210	-2527.670528	-1486.228322	-1484.973404

ΔE_{el} (B3LYP/TZVP, ε_r = 1) = 45.2321 kJ/mol = 0.4688 eV
ΔE_{el} (B3LYP/TZVP, ε_r = 40) = 61.9618 kJ/mol = 0.6422 eV

E(B3LYP/TZVP) / hartree for the structures optimized at each value of ε_r

ε_r	**1**$(BF_4)_2$	(**1**+2H)$(BF_4)_2$	$C_6Cl_2(CN)_2(OH)_2$	$C_6Cl_2(CN)_2O_2$
1	-2526.372388	-2527.606309	-1486.204688	-1484.953539
40	-2526.451653	-2527.683936	-1486.228543	-1484.973475

ΔE_{el} (B3LYP/TZVP, ε_r = 1) = 45.2321 kJ/mol = 0.4688 eV
ΔE_{el} (B3LYP/TZVP, ε_r = 40) = 59.8220 kJ/mol = 0.6200 eV

Calculations for the reaction $C_6H_4(OH)_2$ + (**1**+2H)$^{4+}$ → $C_6H_4O_2$ + (**1**+4H)$^{4+}$

E(B3LYP/TZVP) / hartree for the structures optimized at ε_r = 1

ε_r	(**1**+2H)$^{4+}$	(**1**+4H)$^{4+}$	$C_6H_4(OH)_2$	$C_6H_4O_2$
1	-1677.301599	-1678.549891	-382.630902	-381.397659
40	-1677.992607	-1679.245223	-382.646936	-381.410799

ΔE_{el} (B3LYP/TZVP, ε_r = 1) = -39.5111 kJ/mol = -0.4095 eV
ΔE_{el} (B3LYP/TZVP, ε_r = 40) = -43.2656 kJ/mol = -0.4484 eV

Calculations for the reaction $C_6H_4(OH)_2$ + (**1**+2H)$(BF_4)_4$ → $C_6H_4O_2$ + (**1**+4H)$(BF_4)_4$

E(B3LYP/TZVP) / hartree for the structures optimized at ε_r = 1

ε_r	(**1**+2H)$(BF_4)_4$	(**1**+4H)$(BF_4)_4$	$C_6H_4(OH)_2$	$C_6H_4O_2$
1	-3376.623575	-3377.888141	-382.630902	-381.397659
40	-3376.698096	-3377.962071	-382.646936	-381.410799

ΔE_{el} (B3LYP/TZVP, ε_r = 1) = -82.2385 kJ/mol = -0.8523 eV
ΔE_{el} (B3LYP/TZVP, ε_r = 40) = -73.0887 kJ/mol = -0.7575 eV

E(B3LYP/TZVP) / hartree for the structures optimized at each value of ε_r

ε_r	(**1**+2H)$(BF_4)_4$	(**1**+4H)$(BF_4)_4$	$C_6H_4(OH)_2$	$C_6H_4O_2$
1	-3376.623575	-3377.888141	-382.630902	-381.397659
40	-3376.710258	-3377.971979	-382.647011	-381.410950

ΔE_{el} (B3LYP/TZVP, ε_r = 1) = -82.2385 kJ/mol = -0.8523 eV
ΔE_{el} (B3LYP/TZVP, ε_r = 40) = -67.3703 kJ/mol = -0.6982 eV

Calculations for the reaction $C_6Cl_4(OH)_2$ + (**1**+2H)$^{4+}$ → $C_6Cl_4O_2$ + (**1**+4H)$^{4+}$

E(B3LYP/TZVP) / hartree for the structures optimized at ε_r = 1

ε_r	(**1**+2H)$^{4+}$	(**1**+4H)$^{4+}$	$C_6Cl_4(OH)_2$	$C_6Cl_4O_2$
1	-1677.301599	-1678.549891	-2220.868853	-2219.631177
40	-1677.992607	-1679.245223	-2220.879151	-2219.642037

ΔE_{el} (B3LYP/TZVP, ε_r = 1) = -27.8723 kJ/mol = -0.2889 eV
ΔE_{el} (B3LYP/TZVP, ε_r = 40) = -40.7005 kJ/mol = -0.4218 eV

Calculations for the reaction $C_6Cl_4(OH)_2$ + (**1**+2H)$(BF_4)_4$ → $C_6Cl_4O_2$ + (**1**+4H)$(BF_4)_4$

E(B3LYP/TZVP) / hartree for the structures optimized at ε_r = 1

ε_r	(**1**+2H)$(BF_4)_4$	(**1**+4H)$(BF_4)_4$	$C_6Cl_4(OH)_2$	$C_6Cl_4O_2$
1	-3376.623575	-3377.888141	-2220.868853	-2219.631177
40	-3376.698096	-3377.962071	-2220.879151	-2219.642037

ΔE_{el} (B3LYP/TZVP, ε_r = 1) = -70.5997 kJ/mol = -0.7317 eV
ΔE_{el} (B3LYP/TZVP, ε_r = 40) = -70.5235 kJ/mol = -0.7309 eV

E(B3LYP/TZVP) / hartree for the structures optimized at each value of ε_r

ε_r	(**1**+2H)$(BF_4)_4$	(**1**+4H)$(BF_4)_4$	$C_6Cl_4(OH)_2$	$C_6Cl_4O_2$
1	-3376.623575	-3377.888141	-2220.868853	-2219.631177
40	-3376.710258	-3377.971979	-2220.879308	-2219.642096

ΔE_{el} (B3LYP/TZVP, ε_r = 1) = -70.5997 kJ/mol = -0.7317 eV
ΔE_{el} (B3LYP/TZVP, ε_r = 40) = -64.3484 kJ/mol = -0.6669 eV

Calculations for the reaction $C_6Cl_4O_2$ + (**1**+2H)$(BF_4)_4$ → $C_6Cl_4O_2(HF)_2(BF_3)_2$ + **1**$(BF_4)_2$

E(B3LYP/TZVP) / hartree for the structures optimized at $\varepsilon_r = 1$

ε_r	(**1**+2H)$(BF_4)_4$	**1**$(BF_4)_2$	$C_6Cl_4O_2$	$C_6Cl_4O_2(HF)_2(BF_3)_2$
1	-3376.623575	-2526.372388	-2219.631177	-3069.758291
40	-3376.698096	-2526.439210	-2219.642037	-3069.776298

ΔE_{el} (B3LYP/TZVP, ε_r = 1) = 325.7536 kJ/mol = 3.3762 eV
ΔE_{el} (B3LYP/TZVP, ε_r = 40) = 327.2029 kJ/mol = 3.3912 eV

E(B3LYP/TZVP) / hartree for the structures optimized at each value of ε_r

ε_r	(**1**+2H)$(BF_4)_4$	**1**$(BF_4)_2$	$C_6Cl_4O_2$	$C_6Cl_4O_2(HF)_2(BF_3)_2$
1	-3376.623575	-2526.372388	-2219.631177	-3069.758291
40	-3376.710258	-2526.451653	-2219.642096	-3069.779806

ΔE_{el} (B3LYP/TZVP, ε_r = 1) = 325.7536 kJ/mol = 3.3762 eV
ΔE_{el} (B3LYP/TZVP, ε_r = 40) = 317.4098 kJ/mol = 3.2897 eV

Calculations for the reaction $C_6Cl_2(CN)_2(OH)_2$ + (**1**+2H)$^{4+}$ → $C_6Cl_2(CN)_2O_2$ + (**1**+4H)$^{4+}$

E(B3LYP/TZVP) / hartree for the structures optimized at $\varepsilon_r = 1$

ε_r	(**1**+2H)$^{4+}$	(**1**+4H)$^{4+}$	$C_6Cl_2(CN)_2(OH)_2$	$C_6Cl_2(CN)_2O_2$
1	-1677.301599	-1678.549891	-1486.204688	-1484.953539
40	-1677.992607	-1679.245223	-1486.228322	-1484.973404

ΔE_{el} (B3LYP/TZVP, ε_r = 1) = 7.5011 kJ/mol = 0.0777 eV
ΔE_{el} (B3LYP/TZVP, ε_r = 40) = 6.0439 kJ/mol = 0.0626 eV

Calculations for the reaction $C_6Cl_2(CN)_2(OH)_2$ + (**1**+2H)$(BF_4)_4$ → $C_6Cl_2(CN)_2O_2$ + (**1**+4H)$(BF_4)_4$

E(B3LYP/TZVP) / hartree for the structures optimized at $\varepsilon_r = 1$

ε_r	(**1**+2H)$(BF_4)_4$	(**1**+4H)$(BF_4)_4$	$C_6Cl_2(CN)_2(OH)_2$	$C_6Cl_2(CN)_2O_2$
1	-3376.623575	-3377.888141	-1486.204688	-1484.953539
40	-3376.698096	-3377.962071	-1486.228322	-1484.973404

ΔE_{el} (B3LYP/TZVP, ε_r = 1) = -35.2263 kJ/mol = -0.3651 eV
ΔE_{el} (B3LYP/TZVP, ε_r = 40) = -23.7792 kJ/mol = -0.2465 eV

E(B3LYP/TZVP) / hartree for the structures optimized at each value of ε_r

ε_r	(**1**+2H)$(BF_4)_4$	(**1**+4H)$(BF_4)_4$	$C_6Cl_2(CN)_2(OH)_2$	$C_6Cl_2(CN)_2O_2$
1	-3376.623575	-3377.888141	-1486.204688	-1484.953539
40	-3376.710258	-3377.971979	-1486.228543	-1484.973475

ΔE_{el} (B3LYP/TZVP, ε_r = 1) = -35.2263 kJ/mol = -0.3651 eV
ΔE_{el} (B3LYP/TZVP, ε_r = 40) = -17.4674 kJ/mol = -0.1810 eV

Calculations for the reaction $(C_6Cl_4O_2{+}2H)^{2+}$ + 2 BF_4^- → $C_6Cl_4O_2(HF)_2(BF_3)_2$

E(B3LYP/TZVP) / hartree for the structures optimized at ε_r = 1

ε_r	$(C_6Cl_4O_2{+}2H)^{2+}$	BF_4^-	$C_6Cl_4O_2(HF)_2(BF_3)_2$
1	-2220.102422	-424.575347	-3069.758291
40	-2220.394034	-424.669803	-3069.776298

ΔE_{el} (B3LYP/TZVP, ε_r = 1) = -1326.3368 kJ/mol = -13.7465 eV
ΔE_{el} (B3LYP/TZVP, ε_r = 40) = -111.9986 kJ/mol = -1.1608 eV

Calculations for the reaction $C_6H_4O_2$ + (**1**+2H)$(BF_4)_4$ → $C_6H_4O_2(HF)_2(BF_3)_2$ + **1**$(BF_4)_2$

E(B3LYP/TZVP) / hartree for the structures optimized at ε_r = 1

ε_r	(**1**+2H)$(BF_4)_4$	**1**$(BF_4)_2$	$C_6H_4O_2$	$C_6H_4O_2(HF)_2(BF_3)_2$
1	-3376.623575	-2526.372388	-381.397659	-1231.535578
40	-3376.698096	-2526.439210	-381.410799	-1231.555635

ΔE_{el} (B3LYP/TZVP, ε_r = 1) = 297.3851 kJ/mol = 3.0822 eV
ΔE_{el} (B3LYP/TZVP, ε_r = 40) = 299.4382 kJ/mol = 3.1035 eV

E(B3LYP/TZVP) / hartree for the structures optimized at each value of ε_r

ε_r	(**1**+2H)$(BF_4)_4$	**1**$(BF_4)_2$	$C_6H_4O_2$	$C_6H_4O_2(HF)_2(BF_3)_2$
1	-3376.623575	-2526.372388	-381.397659	-1231.535578
40	-3376.710258	-2526.451653	-381.410950	-1231.573823 (structure changes to $(C_6H_4O_2{+}2H)(BF_4)_2$)

ΔE_{el} (B3LYP/TZVP, ε_r = 1) = 297.3851 kJ/mol = 3.0822 eV
ΔE_{el} (B3LYP/TZVP, ε_r = 40) = 251.3443 kJ/mol = 2.6050 eV

Calculations for the reaction $(C_6H_4O_2{+}2H)^{2+}$ + 2 BF_4^- → $C_6H_4O_2(HF)_2(BF_3)_2$

E(B3LYP/TZVP) / hartree for the structures optimized at ε_r = 1

ε_r	$(C_6H_4O_2{+}2H)^{2+}$	BF_4^-	$C_6H_4O_2(HF)_2(BF_3)_2$
1	-381.863637	-424.575347	-1231.535578
40	-382.191174	-424.669803	-1231.555635

ΔE_{el} (B3LYP/TZVP, ε_r = 1) = -1368.5338 kJ/mol = -14.1839 eV
ΔE_{el} (B3LYP/TZVP, ε_r = 40) = -65.2568 kJ/mol = -0.6763 eV

Analysis of the influence of the relative solvent permittivity (dielectric constant) ε_r on the reaction energy

The reaction between dihydro-benzoquinones and $\mathbf{1}^{2+}$ / $\mathbf{1}(BF_4)_2$ was chosen to study the solvent effect in more detail.

A) Calculations without anions ("single-point" calculations at the optimized structure at $\varepsilon_r = 1$)

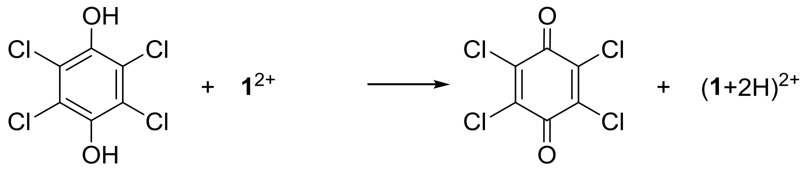

ε_r	ΔE / kJ mol^{-1}
1	9.4045
2	7.7216
5	6.0176
10	5.2668
20	4.8467
40	4.6261
60	4.5500
80	4.5132

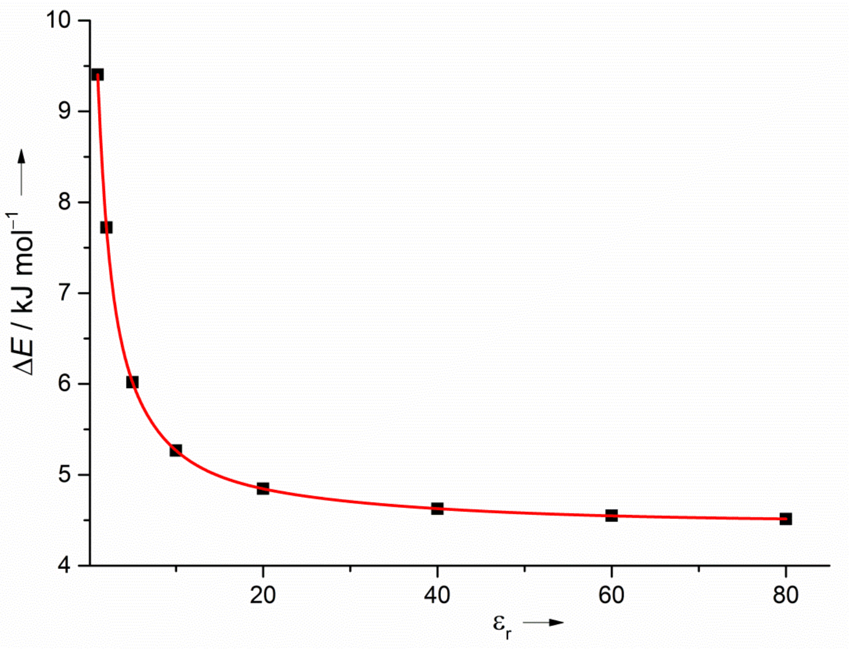

As expected, the effect of solvation on the electronic energy of all compounds (especially the charged ones) is substantial. Nevertheless, the effect on the reaction energy ΔE is only small (decrease of ΔE from 9.4 kJ mol^{-1} at $\varepsilon_r = 1$ to 4.5 kJ mol^{-1} at $\varepsilon_r = 80$).

B) Calculations with anions ("single-point" calculations for structures at ε_r = 1)

ε_r	**ΔE / kJ mol^{-1}**
1	9.8587
2	12.3451
5	14.1357
10	14.7579
20	15.0677
40	15.2174
80	15.2909

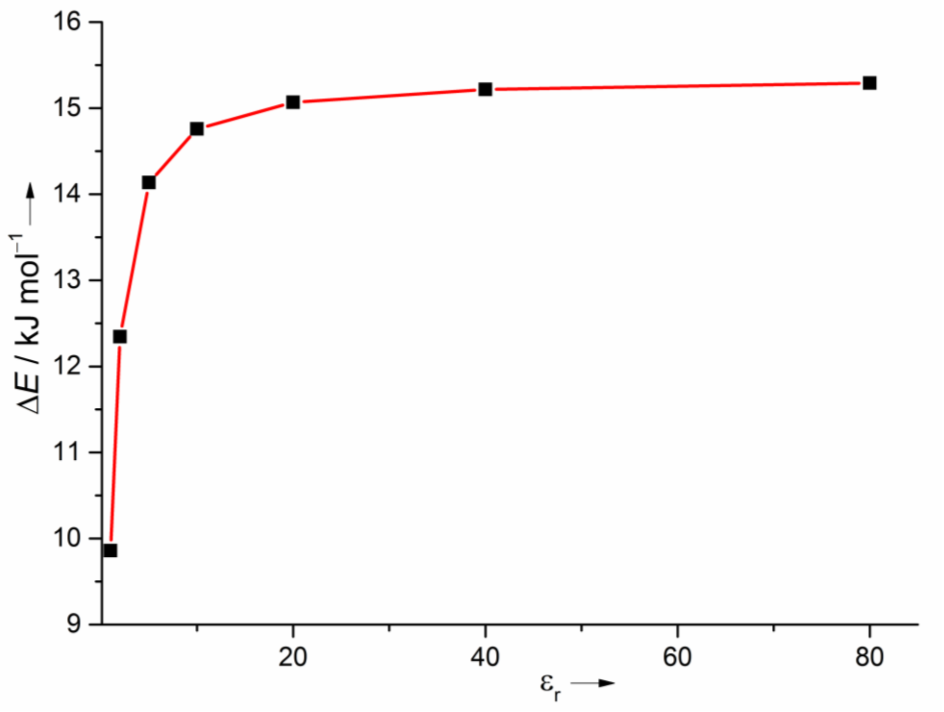

With inclusion of the anions in of **1**$(BF_4)_2$ and (**1**+2H)$(BF_4)_2$, the reactions energy ΔE slightly increases from 9.9 kJ mol^{-1} at ε_r = 1 to 15.3 kJ mol^{-1} at ε_r = 80.

C) Calculations with anions (optimized structures at each ε_r value)

ε_r	**ΔE / kJ mol^{-1}**
1	9.8587
2	12.6365
5	15.7582
10	14.9627
20	13.5791
40	12.9411
80	12.4449

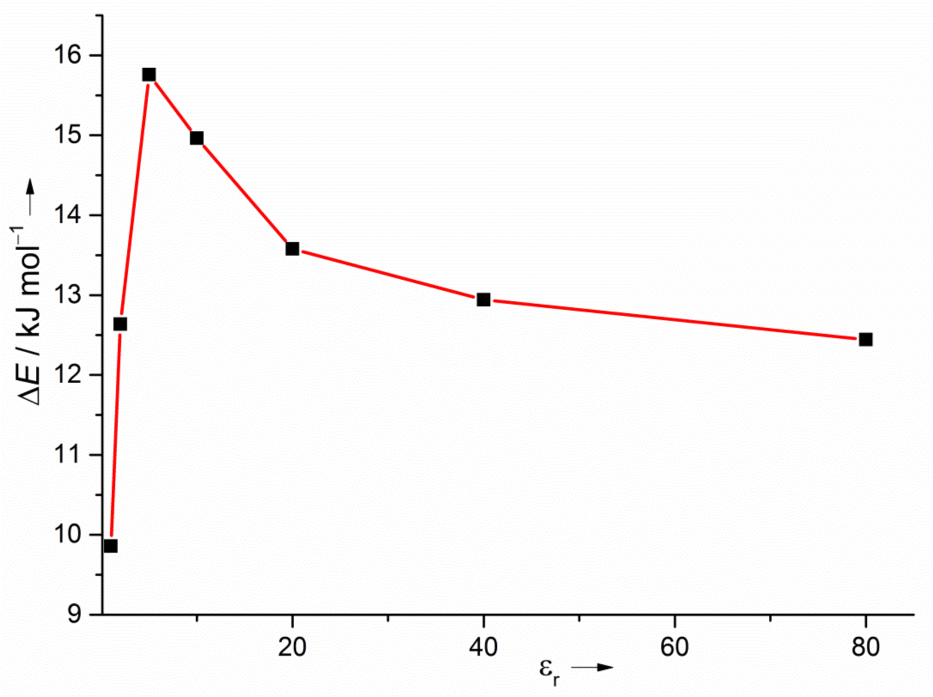

If the structurs are optimized at each ε_r value (with inclusion of the anions), the ΔE vs. ε_r curve (starting with ΔE = 9.9 kJ mol^{-1} at ε_r = 1) passes through a maximum (ΔE = 15.8 kJ mol^{-1} at ε_r = 5), and then slightly decreases to ΔE = 12.5 kJ mol^{-1} at ε_r = 80. The maximum results from the change of the position/orientation of the anions.

Overall conclusion: The dependence of the reaction energy ΔE on the ε_r value is relatively small. The inclusion of the anions also has only a small effect. Finally, the difference between the ΔE values from single-point calculations at the optimized structure at ε_r = 1 and the ΔE values for optimized structures at each ε_r value is also quite small.

Coordinates for calculated structures at ε_r = 1
B3LYP/def2-TZVP

1$(BF_4)_2$, ε_r = 1

Illustration of the structure (H atoms omitted)

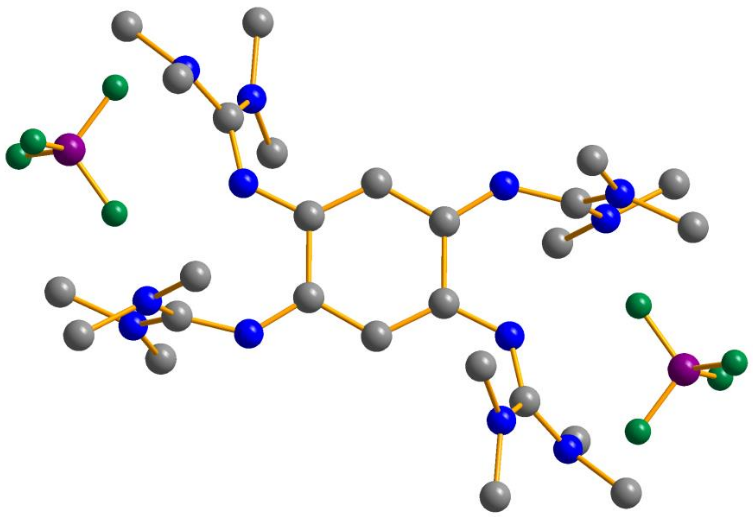

Energy = -2526.372388050 hartree

<u>Coordinates in Å</u>

F	-5.7429903	0.8957755	-1.0474906
B	-6.3372872	1.9632514	-1.7837718
F	-7.6900456	1.7386594	-1.9470610
F	-5.6843582	2.0509463	-3.0407220
F	-6.1023684	3.1629867	-1.0710086
N	-1.8046572	2.0809172	-0.4258268
C	-0.9250080	1.0841323	-0.3061112
C	-2.2465425	2.5706110	-1.5950280
C	-0.8907717	0.4127862	1.0474429
C	-0.0268260	0.6461200	-1.2574432
N	-2.6107547	1.7845934	-2.6305843
N	-2.3820875	3.9138351	-1.7117376
N	-1.6610053	0.7708534	2.0292996
C	0.0267363	-0.6463650	1.2579784
C	0.8903734	-0.4133336	-1.0470589
H	-0.0137954	1.1066148	-2.2354018
C	-2.6620878	2.2478379	-4.0138699
C	-3.0023178	0.3915528	-2.4576191
C	-3.5262600	4.5480266	-2.3758285
C	-1.6405044	4.7896017	-0.8185858
C	-2.6293982	1.6871194	2.1837086
C	0.9249391	-1.0843410	0.3066461
H	0.0137866	-1.1068120	2.2359583
N	1.6601608	-0.7718884	-2.0291222
H	-2.2130526	-0.2800831	-2.8089978
H	-3.9177087	0.2244749	-3.0225624
H	-3.2192317	0.1881209	-1.4158243
H	-3.2003963	5.1665443	-3.2159928
H	-4.0357723	5.1843215	-1.6495507
H	-4.2459307	3.8072595	-2.7060734
H	-2.2036119	5.0024740	0.0959281

H	-1.4531131	5.7328644	-1.3348758
H	-0.6873266	4.3434054	-0.5500635
N	-2.3111074	2.8879669	2.7404288
N	-3.9117906	1.3490005	2.0094946
N	1.8048650	-2.0808738	0.4265612
C	2.6281260	-1.6885880	-2.1837967
C	-0.9304160	3.3484214	2.6802438
C	-3.0883873	3.4600617	3.8402940
C	-4.3016041	-0.0300080	1.7176803
C	-4.9859799	2.3323343	1.8353260
C	2.2475354	-2.5696441	1.5958540
N	2.3090204	-2.8896920	-2.7395195
N	3.9107735	-1.3507266	-2.0108769
H	-0.9221016	4.4287237	2.8273845
H	-0.3133391	2.8850365	3.4576592
H	-0.5039559	3.1347580	1.7059287
H	-3.9205509	2.8150255	4.1016742
H	-2.4432926	3.5519620	4.7184770
H	-3.4735450	4.4508252	3.5891634
H	-3.5176137	-0.7128841	2.0313658
H	-5.2150754	-0.2513124	2.2723391
H	-4.5180111	-0.1503320	0.6557709
H	-5.5448756	2.0705758	0.9395571
H	-5.6607108	2.3381030	2.6950464
H	-4.5698188	3.3236654	1.6902406
N	2.6124545	-1.7827050	2.6304640
N	2.3831751	-3.9127624	1.7136042
C	0.9282761	-3.3497925	-2.6777911
C	3.0851963	-3.4626502	-3.8397138
C	4.3012572	0.0283783	-1.7203744
C	4.9848222	-2.3342590	-1.8370091
C	2.6647704	-2.2447214	4.0141188
C	3.0036971	-0.3897629	2.4559758
C	3.5277993	-4.5463877	2.3774450
C	1.6408893	-4.7892706	0.8217651
H	0.9195731	-4.4301782	-2.8243347
H	0.3105691	-2.8866878	-3.4548871
H	0.5027841	-3.1355107	-1.7031694
H	3.9172071	-2.8178940	-4.1023147
H	2.4392595	-3.5550636	-4.7172280
H	3.4704603	-4.4532981	-3.5882673
H	3.5170655	0.7112490	-2.0336152
H	5.2141596	0.2491090	-2.2762138
H	4.5188768	0.1492968	-0.6587696
H	5.5445809	-2.0720726	-0.9418907
H	5.6587979	-2.3407966	-2.6973219
H	4.5684842	-3.3253787	-1.6909081
H	3.6951669	-2.3575532	4.3529960
H	2.1707295	-1.4943711	4.6370062
H	2.1303827	-3.1842235	4.1214663
H	2.2145522	0.2820775	2.8072322
H	3.9194225	-0.2220284	3.0201886
H	3.2199269	-0.1872595	1.4138412
H	3.2025307	-5.1640837	3.2184443
H	4.0367390	-5.1834055	1.6513936
H	4.2477523	-3.8053345	2.7064492
H	2.2032989	-5.0029643	-0.0930001
H	1.4538405	-5.7320802	1.3390078
H	0.6875178	-4.3432598	0.5535704
H	-3.6922403	2.3611631	-4.3533229
H	-2.1677908	1.4979335	-4.6370947

H	-2.1274432	3.1873260	-4.1200412
F	6.1025822	-3.1623846	1.0695747
B	6.3383825	-1.9621327	1.7811971
F	5.7437763	-0.8950976	1.0445317
F	7.6913341	-1.7377685	1.9432090
F	5.6864532	-2.0486557	3.0387404

(**1**+2H)$(BF_4)_2$, ε_r = 1

Illustration of the structure (C-H atoms omitted)

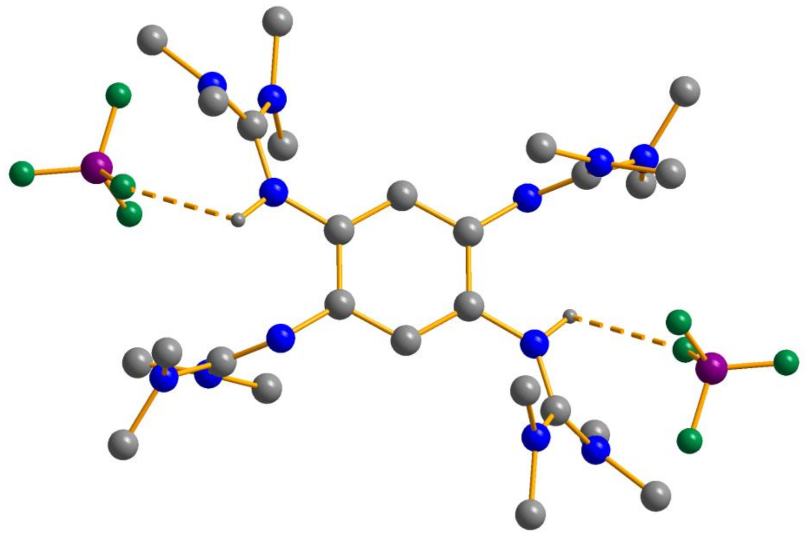

Energy = -2527.606308625 hartree

<u>Coordinates in Å</u>

F	-5.9291476	1.1879791	-0.9327727
B	-5.7448328	2.5268806	-1.3217085
F	-6.8193454	3.3155423	-0.9566299
F	-5.5399756	2.5716101	-2.7275033
F	-4.5514179	3.0156388	-0.6958963
N	-1.5648679	2.2078811	-0.7356396
C	-0.8167482	1.0583911	-0.3895458
C	-2.0556409	2.6141053	-1.9262716
C	-0.6624376	0.7939279	0.9887155
C	-0.1676079	0.2749384	-1.3369730
N	-2.5202239	1.7322406	-2.8173374
N	-2.0307557	3.9351703	-2.1944486
N	-1.1391887	1.6612175	1.9691595
C	0.1680467	-0.2741558	1.3363474
C	0.6628739	-0.7931525	-0.9893491
H	-0.2927528	0.4824004	-2.3900658
C	-2.5452545	1.9941423	-4.2513844
C	-3.1683231	0.4843197	-2.4113912
C	-3.1181581	4.6156761	-2.9054519
C	-1.1046124	4.8274993	-1.5069396
C	-2.3509159	1.7927839	2.4216535
C	0.8170697	-1.0577106	0.3889116
H	0.2932502	-0.4815984	2.3894339
N	1.1397234	-1.6603173	-1.9698809
H	-2.5508809	-0.3757657	-2.6796843

H	-4.1336246	0.4284246	-2.9117858
H	-3.3549396	0.4914322	-1.3443901
H	-2.7504719	5.0686376	-3.8294761
H	-3.5094469	5.4023687	-2.2579082
H	-3.9342377	3.9305512	-3.1057464
H	-1.5695323	5.2935490	-0.6318135
H	-0.8153429	5.6225362	-2.1960540
H	-0.2105037	4.2918240	-1.1978053
N	-2.6324310	2.9336081	3.1584224
N	-3.4018139	0.9246061	2.2676308
N	1.5649014	-2.2074089	0.7349678
C	2.3516788	-1.7920384	-2.4217824
C	-1.6781327	4.0272152	3.1379486
C	-3.3718005	2.8394103	4.4120830
C	-3.1992022	-0.4911590	2.0198185
C	-4.7904912	1.3740033	2.1461344
C	2.0550176	-2.6140811	1.9257308
N	2.6333595	-2.9327432	-3.1585851
N	3.4026094	-0.9240081	-2.2670798
H	-2.1803727	4.9299719	3.4907597
H	-0.8078407	3.8344696	3.7770058
H	-1.3135108	4.1988320	2.1285106
H	-3.8951508	1.8919179	4.4808750
H	-2.6855911	2.9123364	5.2653594
H	-4.1027805	3.6475805	4.4919368
H	-2.2477694	-0.8193663	2.4278935
H	-3.9972136	-1.0458918	2.5197093
H	-3.2312974	-0.7456942	0.9556590
H	-5.2235110	0.9769831	1.2278314
H	-5.3922618	1.0355797	2.9965369
H	-4.8318082	2.4561517	2.0819353
N	2.5198602	-1.7325511	2.8169885
N	2.0291967	-3.9351421	2.1937854
C	1.6789293	-4.0262420	-3.1388635
C	3.3739883	-2.8387295	-4.4115063
C	3.2000113	0.4917719	-2.0193379
C	4.7911466	-1.3736159	-2.1447591
C	2.5444207	-1.9945642	4.2510249
C	3.1687695	-0.4849731	2.4112775
C	3.1158384	-4.6164229	2.9052089
C	1.1026759	-4.8268192	1.5059328
H	2.1813703	-4.9291096	-3.4911077
H	0.8092652	-3.8334503	-3.7787636
H	1.3133280	-4.1976773	-2.1297506
H	3.8967280	-1.8908941	-4.4802503
H	2.6886759	-2.9125479	-5.2654127
H	4.1056113	-3.6464324	-4.4902480
H	2.2488754	0.8201142	-2.4280108
H	3.9984069	1.0464320	-2.5186939
H	3.2314329	0.7462660	-0.9551459
H	5.2238289	-0.9763390	-1.2264080
H	5.3934140	-1.0356335	-2.9949838
H	4.8322400	-2.4557480	-2.0801089
H	3.5575116	-2.2169575	4.5902098
H	2.1921291	-1.0989677	4.7675027
H	1.8832641	-2.8207802	4.4978110
H	2.5518152	0.3754448	2.6796306
H	4.1340570	-0.4297307	2.9117724
H	3.3554507	-0.4920483	1.3442854
H	2.7474909	-5.0690546	3.8291303
H	3.5067525	-5.4034499	2.2578473

H	3.9323661	-3.9319021	3.1057402
H	1.5676102	-5.2932037	0.6309946
H	0.8125757	-5.6216418	2.1949451
H	0.2090605	-4.2905212	1.1964537
H	-3.5585549	2.2158726	-4.5903816
H	-2.1925059	1.0987372	-4.7678841
H	-1.8846814	2.8207670	-4.4983646
F	4.5511313	-3.0172887	0.6964840
B	5.7445729	-2.5293515	1.3229094
F	5.9302207	-1.1907216	0.9337651
F	6.8186575	-3.3190168	0.9587051
F	5.5388260	-2.5735723	2.7285948
H	1.7201806	-2.8313281	-0.0453945
H	-1.7203079	2.8317803	0.0447059

($\mathbf{1}$+2H)$(BF_4)_4$, $\varepsilon_r = 1$

Illustration of the structure (C-H atoms omitted)

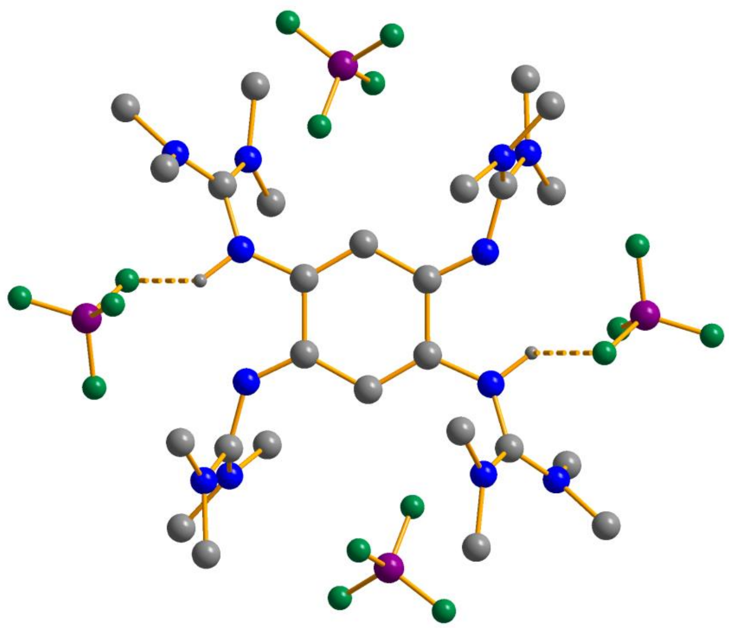

Energy = -3376.623575238 hartree

<u>Coordinates in Å</u>

F	5.8570700	1.7111454	4.3644926
B	5.3371698	2.9816370	4.2602899
F	4.1158331	3.0595393	5.0456497
F	5.0206599	3.3207307	2.9405328
F	6.2245081	3.9413943	4.8059997
C	2.9415904	8.9604363	6.6217603
C	3.6010814	7.7354131	6.9947232
C	2.5044299	8.0714062	4.3599248
C	3.6847675	6.6972544	6.1286336
C	3.1641788	6.8464731	4.7327985
N	3.4183787	5.8643990	3.9473297
F	1.4935985	8.2147259	1.3485472
B	3.7513789	6.6242648	10.6284482
B	2.3514150	9.1814677	0.7258269

F	3.0102446	7.2553451	11.6236827
F	2.8910268	6.1212258	9.6210225
F	4.5351405	5.5857611	11.1346680
F	3.0914773	8.5496412	-0.2697714
F	3.2128471	9.6851408	1.7319908
F	1.5671580	10.2196033	0.2197104
N	2.6878324	9.9427564	7.4070679
C	2.4207324	9.1095390	5.2260293
N	4.2217020	5.4662923	6.4124892
C	3.1590556	5.6961658	2.6155225
F	4.6098394	7.5914142	10.0072924
C	2.9480886	10.1113447	8.7386342
N	1.8836681	10.3404700	4.9422192
H	4.0260396	4.7042041	5.7495579
C	5.1396608	5.1250038	7.3810465
N	4.0453623	6.1162594	1.7218178
N	2.0776735	4.9727107	2.2866925
N	2.0619799	9.6923815	9.6330222
N	4.0303265	10.8340429	9.0664576
H	2.0798636	11.1027427	5.6047829
C	0.9651572	10.6815774	3.9741224
N	4.9467738	3.9774830	8.0184174
N	6.2163303	5.8906399	7.5847815
C	5.0806494	7.0922360	2.0641439
C	4.2769587	5.4260070	0.4493263
C	1.3905858	5.1000127	1.0012487
C	1.4178858	4.1241773	3.2791970
C	1.0255627	8.7172728	9.2915802
C	1.8318206	10.3831575	10.9054720
C	4.7184376	10.7063818	10.3513128
C	4.6901040	11.6816421	8.0731423
N	1.1578094	11.8288468	3.3362315
N	-0.1116273	9.9158969	3.7711771
C	3.6138228	3.3895144	8.1802879
C	6.0504008	3.1358763	8.4772408
C	6.9142120	5.9620287	8.8701614
C	6.7788791	6.7164667	6.5196491
C	2.4907422	12.4165760	3.1733353
C	0.0540085	12.6704189	2.8777498
C	-0.8102032	9.8441382	2.4861996
C	-0.6735029	9.0902471	4.8368005
F	1.9920756	12.7473594	6.3087610
F	-0.1180484	11.8686493	6.5471043
B	0.7705496	12.8267132	7.0937350
F	0.2527115	14.0980902	6.9900388
F	1.0860159	12.4863807	8.4133976
H	3.9600952	7.6441080	8.0113216
H	2.1452732	8.1625693	3.3433571
H	0.1348444	9.2346933	8.9258589
H	0.7901153	8.1558549	10.1928605
H	1.3816281	8.0030020	8.5591322
H	2.1887138	9.7810952	11.7411953
H	0.7569795	10.5357836	11.0029357
H	2.3016892	11.3601710	10.8954279
H	4.5823676	11.5968088	10.9689031
H	5.7837505	10.5860492	10.1469917
H	4.3837194	9.8190877	10.8767613
H	5.5010188	11.1493215	7.5666855
H	5.1138271	12.5397984	8.5946563
H	3.9662655	12.0451393	7.3490456
H	2.8561517	4.1600598	8.0947362

H	3.5547324	2.9711938	9.1847103
H	3.4587128	2.6063137	7.4370716
H	6.9799503	3.4486674	8.0128516
H	5.8404116	2.1150994	8.1567527
H	6.1409876	3.1596473	9.5643189
H	6.3035956	5.5400979	9.6611603
H	7.0710125	7.0141176	9.1070831
H	7.8829933	5.4602948	8.8173007
H	6.4429643	6.3579642	5.5515074
H	7.8647705	6.6229335	6.5574270
H	6.5136510	7.7682313	6.6540628
H	3.2483589	11.6459537	3.2586629
H	2.5492626	12.8345606	2.1687416
H	2.6464504	13.1999855	3.9162044
H	-0.8752621	12.3580064	3.3429434
H	0.2643862	13.6913095	3.1976133
H	-0.0373184	12.6461500	1.7907450
H	-0.2000749	10.2659628	1.6947714
H	-0.9670285	8.7919807	2.2495953
H	-1.7790037	10.3457911	2.5394829
H	-0.3371041	9.4490067	5.8046779
H	-1.7594243	9.1836408	4.7995880
H	-0.4082268	8.0384795	4.7025091
H	5.9716683	6.5755839	2.4302354
H	5.3161252	7.6541248	1.1631609
H	4.7234942	7.8059830	2.7965682
H	3.9198482	6.0278700	-0.3864446
H	5.3520165	5.2745552	0.3524198
H	3.8081567	4.4484757	0.4589274
H	1.5280086	4.2098113	0.3836330
H	0.3249952	5.2193831	1.2046595
H	1.7249985	5.9876892	0.4762483
H	0.6059423	4.6555309	3.7850181
H	0.9955689	3.2656865	2.7570905
H	2.1414407	3.7612738	4.0038768

($\mathbf{1}$+4H)(BF_4)$_4$, ε_r = 1

Illustration of the structure (C-H atoms omitted)

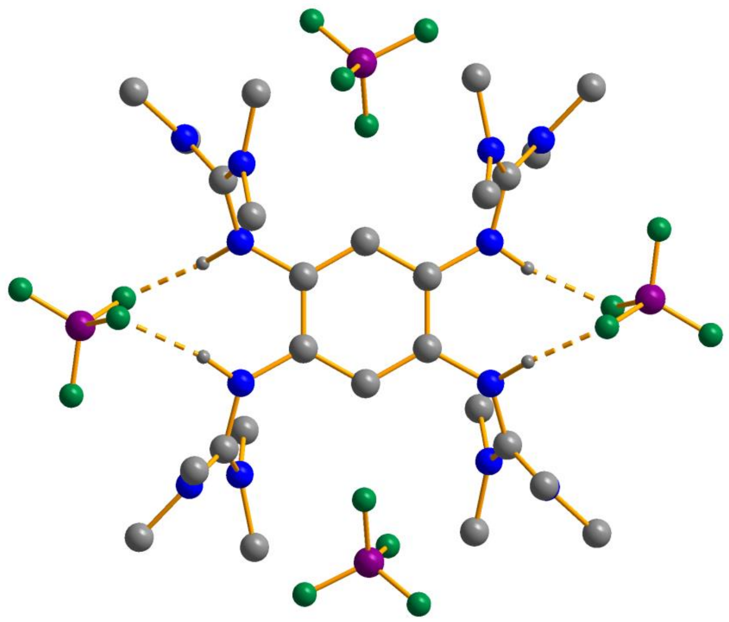

Energy = -3377.888141278 hartree

Coordinates in Å

F	2.8419913	-5.9706793	-1.3343576
B	2.0967978	-4.8732892	-1.6797975
F	1.0055502	-4.7053610	-0.7510545
F	1.5703702	-4.9409692	-2.9658626
F	2.8990470	-3.6791527	-1.5776916
C	-0.3982685	0.8942293	1.0078163
C	0.3210216	-0.2530217	1.3265420
C	-0.3210281	0.2544107	-1.3266058
C	0.7181415	-1.1464683	0.3382167
C	0.3981546	-0.8928819	-1.0078591
N	0.9064911	-1.7461754	-2.0080537
F	-1.1869046	1.2283722	-4.2573975
B	-0.0382377	-1.5916083	4.9186356
B	0.0374640	1.5909896	-4.9197570
F	-0.3511230	-0.5765133	5.8369417
F	-1.0414187	-1.6616028	3.9270258
F	0.1295706	-2.8180522	5.5411671
F	0.3508410	0.5753333	-5.8372810
F	1.0405999	1.6621827	-3.9281985
F	-0.1309229	2.8168719	-5.5432283
N	-0.9064137	1.7474941	2.0080935
C	-0.7183544	1.1477533	-0.3382388
N	1.3495702	-2.3653170	0.6789593
C	0.3952529	-1.9913329	-3.2463965
F	1.1862974	-1.2290605	4.2565818
C	-0.3952439	1.9917239	3.2466937
N	-1.3501788	2.3663930	-0.6791044
H	1.0332712	-3.2110607	0.1981337
C	2.2962455	-2.5817448	1.6226579
N	1.2464103	-2.0530468	-4.2661707
N	-0.9184609	-2.2198883	-3.4031956

N	-1.2463331	2.0523207	4.2665606
N	0.9184373	2.2202566	3.4035820
H	-1.0337711	3.2125477	-0.1990607
C	-2.2968755	2.5820136	-1.6229706
N	2.2132102	-3.7034877	2.3392885
N	3.3209296	-1.7227078	1.7737555
C	2.5222008	-1.3345786	-4.2553053
C	1.0401233	-2.9289170	-5.4162457
C	-1.6417328	-1.8662488	-4.6236128
C	-1.7130681	-2.8247563	-2.3391641
C	-2.5220510	1.3337442	4.2550701
C	-1.0400875	2.9271313	5.4174535
C	1.6418200	1.8659207	4.6237201
C	1.7127936	2.8265712	2.3401819
N	-2.2138631	3.7030372	-2.3407074
N	-3.3215443	1.7227543	-1.7732117
C	0.9280830	-4.3590751	2.6092553
C	3.3886555	-4.4255677	2.8158885
C	3.9643644	-1.4828582	3.0630360
C	3.8167039	-0.9281012	0.6545220
C	-0.9287580	4.3584597	-2.6112175
C	-3.3892736	4.4244018	-2.8184425
C	-3.9648229	1.4812887	-3.0622681
C	-3.8170441	0.9291163	-0.6531620
F	-1.0067010	4.7082751	0.7476238
F	-2.8978804	3.6819648	1.5793647
B	-2.0937644	4.8748004	1.6813931
F	-2.8390047	5.9741960	1.3427723
F	-1.5615872	4.9379451	2.9654911
H	0.4994373	-0.5079135	2.3621503
H	-0.4992119	0.5093724	-2.3622552
H	-2.4651342	0.4821396	3.5846146
H	-3.3357125	2.0031213	3.9663144
H	-2.6976883	0.9510072	5.2593671
H	-0.7898333	2.3468941	6.3071010
H	-1.9671780	3.4737279	5.5912141
H	-0.2634423	3.6534162	5.2027590
H	1.9304102	2.7580517	5.1848145
H	2.5458403	1.3259070	4.3393193
H	1.0438329	1.2022084	5.2399954
H	2.2980280	2.0785768	1.7985219
H	2.3990737	3.5436034	2.7929144
H	1.0699879	3.3633301	1.6480308
H	0.1146113	-3.6502383	2.4902376
H	0.9277938	-4.6875104	3.6480375
H	0.7881813	-5.2133293	1.9442812
H	4.2858271	-4.0505489	2.3326541
H	3.2717424	-5.4764549	2.5474182
H	3.4895296	-4.3485480	3.9003244
H	3.3459711	-1.8698132	3.8656565
H	4.0569001	-0.4042764	3.2037381
H	4.9656258	-1.9191842	3.0994488
H	3.5740809	-1.4147985	-0.2856292
H	4.9028133	-0.8654982	0.7338920
H	3.4080886	0.0856819	0.6603523
H	-0.1152347	3.6498251	-2.4913686
H	-0.9283795	4.6857947	-3.6503458
H	-0.7889992	5.2133926	-1.9470964
H	-4.2865322	4.0498246	-2.3350244
H	-3.2725558	5.4756320	-2.5512320
H	-3.4898489	4.3460979	-3.9028127

H	-3.3464362	1.8674231	-3.8652897
H	-4.0571748	0.4025216	-3.2017156
H	-4.9661590	1.9173970	-3.0992824
H	-3.5746854	1.4169337	0.2864834
H	-4.9031186	0.8659419	-0.7325500
H	-3.4079792	-0.0844967	-0.6578433
H	2.4652752	-0.4822605	-3.5857451
H	3.3357664	-2.0037152	-3.9657398
H	2.6979902	-0.9529352	-5.2599918
H	0.7903343	-2.3494534	-6.3065312
H	1.9670140	-3.4761093	-5.5891732
H	0.2630781	-3.6546413	-5.2010901
H	-1.9305258	-2.7587199	-5.1840649
H	-2.5456049	-1.3257620	-4.3396477
H	-1.0435403	-1.2031374	-5.2403292
H	-2.2977568	-2.0759374	-1.7980454
H	-2.3998760	-3.5417227	-2.7912040
H	-1.0704992	-3.3613521	-1.6466504
H	1.7826595	-2.2310206	-1.8140276
H	-1.7824777	2.2325805	1.8142403

$C_6H_4O_2$ (BQ), $\varepsilon_r = 1$

Illustration of the structure

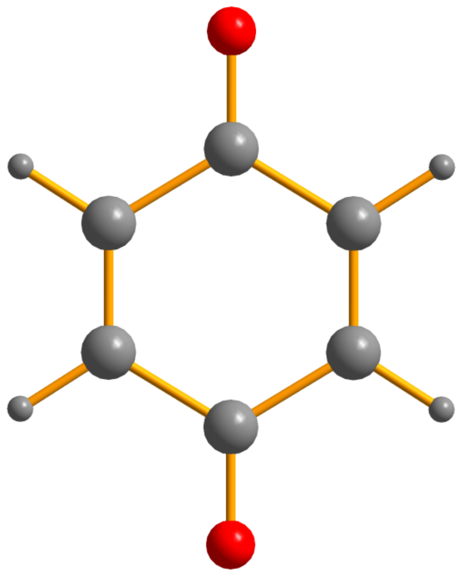

Energy = -381.3976586665 hartree

<u>Coordinates in Å</u>

C	1.4394615	-0.0000004	-0.0002648
C	0.6681633	1.2661677	0.0003196
C	-0.6681607	1.2661685	0.0003945
C	-1.4394562	0.0000005	-0.0001927
C	-0.6681624	-1.2661682	0.0003165
C	0.6681617	-1.2661680	0.0003486
O	2.6573346	-0.0000015	-0.0014168
H	1.2539444	2.1774916	0.0004059
H	-1.2539457	2.1774944	0.0005065
O	-2.6573381	0.0000017	-0.0012447

H	-1.2539460	-2.1774949	0.0003697
H	1.2539435	-2.1774914	0.0004575

$C_6H_4(OH)_2$, ε_r = 1

Illustration of the structure

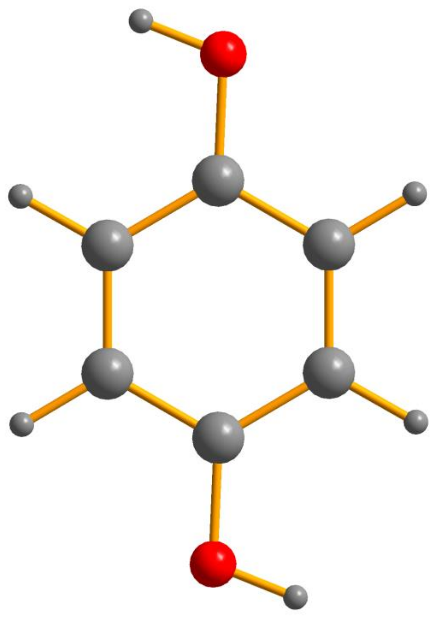

Energy = -382.6309021723 hartree

Coordinates in Å

C	1.3959206	-0.0293616	0.0000988
C	0.7162670	1.1861318	0.0002096
C	-0.6727162	1.2169398	0.0001333
C	-1.3959207	0.0293617	0.0000212
C	-0.7162671	-1.1861319	0.0000233
C	0.6727162	-1.2169396	0.0001504
O	2.7650095	-0.1165930	-0.0002520
H	1.2716448	2.1181840	0.0001779
H	-1.2048008	2.1591512	-0.0000759
O	-2.7650095	0.1165929	-0.0000579
H	-1.2716449	-2.1181843	-0.0001015
H	1.2048009	-2.1591508	0.0002536
H	3.1445676	0.7689073	-0.0004212
H	-3.1445675	-0.7689075	-0.0001595

$(C_6H_4O_2$+2H$)^{2+}$, ε_r = 1

Illustration of the structure

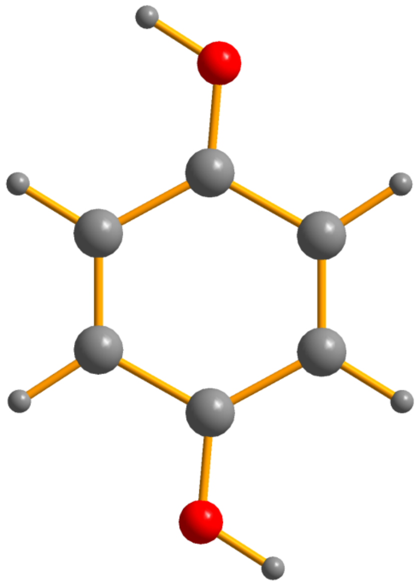

Energy = -381.8636372977 hartree

Coordinates in Å

C	1.3820963	0.0141577	-0.0000216
C	0.6727154	1.2897065	-0.0007568
C	-0.6689336	1.2758506	-0.0008508
C	-1.3821053	-0.0141578	-0.0001083
C	-0.6727262	-1.2897072	-0.0008363
C	0.6689235	-1.2758541	-0.0007892
O	2.6441825	-0.0811072	0.0015170
H	1.2355520	2.2184120	-0.0009927
H	-1.2697255	2.1799882	-0.0010251
O	-2.6441720	0.0811098	0.0014949
H	-1.2355575	-2.2184048	-0.0010616
H	1.2697239	-2.1800011	-0.0009864
H	-3.1695153	-0.7523415	0.0022128
H	3.1695419	0.7523489	0.0022039

$C_6H_4O_2(HF)_2(BF_3)_2$, ε_r = 1

Illustration of the structure

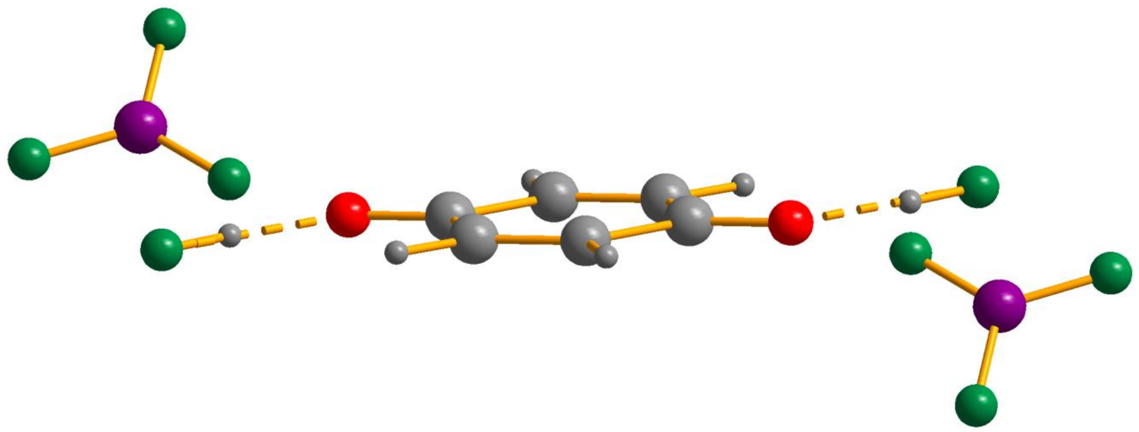

Energy = -1231.535577759 hartree

Coordinates in Å

C	-0.2291494	1.3466984	-0.4100362
C	-1.3100350	0.5301141	0.1772874
C	-1.0062157	-0.8605273	0.5735230
H	-0.4917215	2.3594610	-0.6885589
O	-2.4281087	1.0142585	0.3229566
H	-1.8111819	-1.4390834	1.0093055
H	-3.6796506	0.2392891	0.9371004
B	-4.0559103	-1.1664567	3.3458688
F	-4.3968087	-0.2865806	1.3038983
F	-3.2247087	-2.0994152	2.8879938
F	-5.3236070	-1.4607427	3.5388666
F	-3.5409705	-0.0539373	3.8444319
C	0.2208281	-1.3607874	0.3992580
C	1.3016756	-0.5442272	-0.1882222
C	0.9979935	0.8465571	-0.5840098
H	0.4834629	-2.3735484	0.6778857
O	2.4196611	-1.0286087	-0.3342984
H	1.8031348	1.4254384	-1.0191436
H	3.6749882	-0.2541758	-0.9428780
B	4.0691910	1.1940213	-3.3291159
F	4.3962369	0.2700843	-1.3036219
F	3.2376772	2.1194285	-2.8564857
F	5.3389745	1.4885554	-3.5071909
F	3.5542444	0.0941843	-3.8548145

$C_6Cl_4O_2$ (CA), $\varepsilon_r = 1$

Illustration of the structure

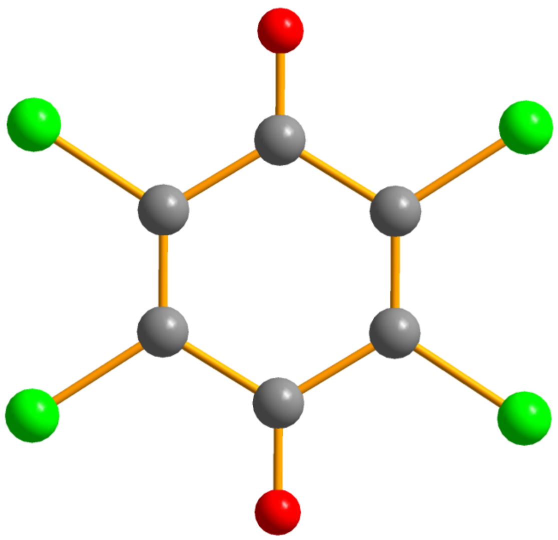

Energy = -2219.631177278 hartree

Coordinates in Å

C	1.4532675	-0.0000100	0.0000636
C	0.6725199	1.2776468	-0.0000822
C	-0.6725229	1.2776936	0.0000595
C	-1.4532293	0.0000129	0.0000183
C	-0.6725205	-1.2776768	-0.0000688
C	0.6725204	-1.2776474	0.0000437
O	2.6587402	0.0000020	0.0002055
Cl	1.6065797	2.7100788	-0.0004087
Cl	-1.6066139	2.7098906	0.0002611
O	-2.6586980	0.0000011	0.0000608
Cl	-1.6066196	-2.7098927	-0.0002778
Cl	1.6065764	-2.7100990	0.0001250

$C_6Cl_4(OH)_2$, ε_r = 1

Illustration of the structure

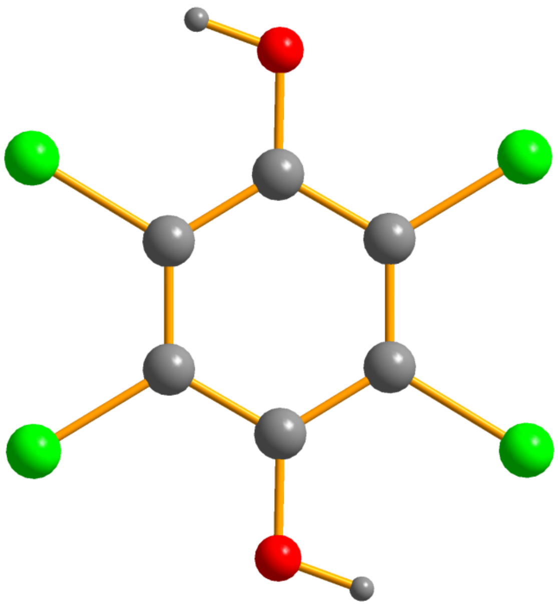

Energy = -2220.868852789 hartree

Coordinates in Å

C	1.4096088	-0.0345987	-0.0000055
C	0.7161868	1.1797023	-0.0000855
C	-0.6769822	1.2243950	0.0001093
C	-1.4096592	0.0347294	-0.0000139
C	-0.7162713	-1.1796021	0.0000956
C	0.6769669	-1.2243244	-0.0000142
O	2.7582765	-0.1036511	0.0000533
Cl	1.6593789	2.6397060	-0.0003209
Cl	-1.5269482	2.7260030	0.0004046
O	-2.7584122	0.1036043	-0.0001917
Cl	-1.6594146	-2.6397386	0.0002774
Cl	1.5268553	-2.7259428	-0.0000830
H	3.1165766	0.7963075	0.0000954
H	-3.1161620	-0.7965899	-0.0003210

$(C_6Cl_4O_2+2H)^{2+}$, $\varepsilon_r = 1$

Illustration of the structure

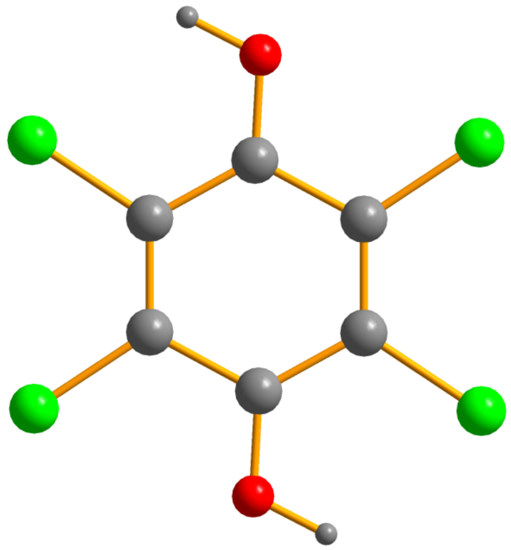

Energy = -2220.102421622

Coordinates in Å

C	1.3884024	0.0158234	-0.0000251
C	0.6954005	1.2849843	-0.0001915
C	-0.6723145	1.2912008	-0.0000563
C	-1.3884582	-0.0158255	0.0000136
C	-0.6954480	-1.2849940	-0.0001372
C	0.6722643	-1.2911965	-0.0000610
O	2.6534332	-0.0571171	0.0002274
Cl	1.6415212	2.6925623	-0.0002753
Cl	-1.5867569	2.6858136	0.0000220
O	-2.6534176	0.0571269	0.0002479
Cl	-1.6414821	-2.6925706	-0.0003699
Cl	1.5868087	-2.6858433	0.0000595
H	-3.1113740	-0.8191953	0.0002935
H	3.1114209	0.8192311	0.0002524

$C_6Cl_4O_2(HF)_2(BF_3)_2$, ε_r = 1

Illustration of the structure

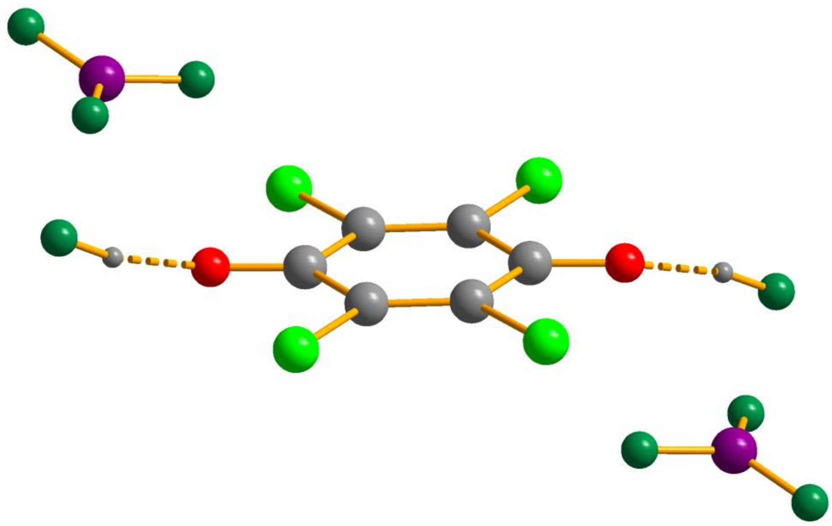

Energy = -3069.758290989 hartree

<u>Coordinates in Å</u>

C	-0.4508800	1.3707070	0.0199408
C	-1.4219516	0.2384800	0.0690943
C	-0.8811251	-1.1503709	0.0289433
Cl	-1.1290283	2.9331709	0.0599470
O	-2.6117448	0.4604932	0.1427122
Cl	-2.0345694	-2.4044974	0.0485655
H	-3.9953954	-0.3125386	0.7713459
B	-4.0778647	-0.9369416	3.5033139
F	-4.7226854	-0.6513262	1.2729966
F	-4.4625704	-2.1965351	3.5450178
F	-4.8630678	0.0175772	3.9577497
F	-2.8125292	-0.6537393	3.2084959
C	0.4466068	-1.3741230	-0.0239002
C	1.4177011	-0.2418756	-0.0726901
C	0.8768853	1.1469494	-0.0324594
Cl	1.1247464	-2.9365854	-0.0641190
O	2.6075048	-0.4639401	-0.1458683
Cl	2.0303168	2.4010802	-0.0514170
H	3.9930223	0.3094004	-0.7697744
B	4.0847410	0.9432856	-3.4995693
F	4.7223092	0.6487019	-1.2681631
F	4.4728125	2.2020314	-3.5358351
F	4.8689037	-0.0116756	-3.9548816
F	2.8178621	0.6622715	-3.2094453

$C_6Cl_2(CN)_2O_2$ (DDQ), $\varepsilon_r = 1$

Illustration of the structure

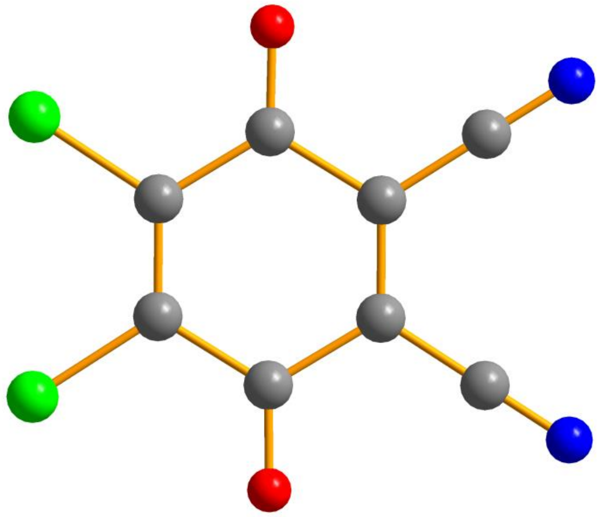

Energy = -1484.953539242 hartree

<u>Coordinates in Å</u>

C	1.4541230	0.0513589	0.0002833
C	0.6748537	1.3238294	0.0002496
C	-0.6748269	1.3238354	0.0001182
C	-1.4541073	0.0513718	-0.0002618
C	-0.6759145	-1.2334638	-0.0001499
C	0.6759189	-1.2334697	-0.0000357
O	2.6594395	0.0325547	0.0005661
Cl	1.6057874	2.7541341	-0.0003888
Cl	-1.6057480	2.7541484	-0.0001119
O	-2.6594239	0.0325782	-0.0007080
C	-1.4417411	-2.4332035	0.0000849
C	1.4417349	-2.4332162	-0.0001618
N	-2.0605033	-3.4046022	0.0002860
N	2.0604884	-3.4046205	-0.0002930

$C_6Cl_2(CN)_2(OH)_2$, $\varepsilon_r = 1$

Illustration of the structure

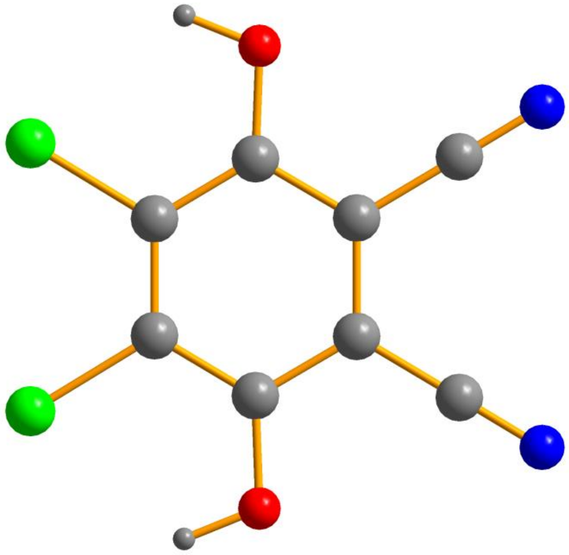

Energy = -1486.204688409 hartree

Coordinates in Å

C	1.4120512	0.0472929	-0.0001089
C	0.6958235	1.2507080	0.0001551
C	-0.6958240	1.2507079	0.0002077
C	-1.4120513	0.0472925	0.0000047
C	-0.7050584	-1.1608556	-0.0000503
C	0.7050587	-1.1608553	-0.0000264
O	2.7553738	-0.0015616	-0.0005004
Cl	1.5959757	2.7302455	0.0002095
Cl	-1.5959766	2.7302450	0.0003285
O	-2.7553740	-0.0015626	-0.0001424
C	-1.4340657	-2.3857273	-0.0001508
C	1.4340666	-2.3857266	0.0002744
H	3.1176874	0.8965946	-0.0005148
H	-3.1176882	0.8965933	-0.0000591
N	-2.0277600	-3.3727516	-0.0003481
N	2.0277613	-3.3727507	0.0007213

Coordinates for calculated structures at ε_r = 40
B3LYP+COSMO/def2-TZVP

1$(BF_4)_2$, ε_r = 40

Illustration of the structure (H atoms omitted)

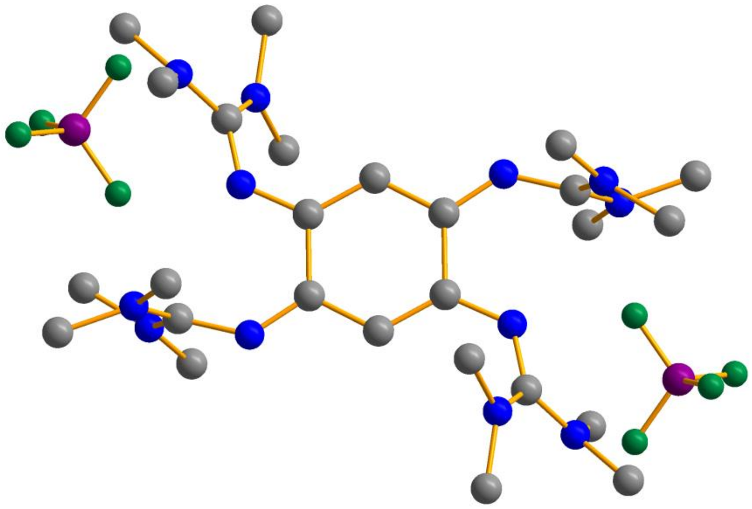

Energy = -2526.451653161 hartee

Coordinates in Å

F	-6.5401505	1.0585744	-1.3260030
B	-7.0057466	2.1798562	-2.0378747
F	-8.3204313	1.9330644	-2.4879008
F	-6.1726238	2.4161781	-3.1495089
F	-7.0040583	3.3079290	-1.1985141
N	-1.8041512	2.0934467	-0.3495274
C	-0.9372709	1.0801824	-0.2698289
C	-2.2794461	2.6030621	-1.4947680
C	-0.8266295	0.4335911	1.0910739
C	-0.1049863	0.6198360	-1.2685335
N	-2.7501320	1.8334803	-2.5062470
N	-2.3673498	3.9458680	-1.6072803
N	-1.5343181	0.8164813	2.1124351
C	0.1045030	-0.6204282	1.2681505
C	0.8270999	-0.4332051	-1.0911019
H	-0.1422107	1.0716468	-2.2503354
C	-2.7522623	2.2615799	-3.9037134
C	-3.1881745	0.4611129	-2.2984539
C	-3.4780759	4.6224556	-2.2777370
C	-1.5345296	4.8098038	-0.7796115
C	-2.5488509	1.7000684	2.2253738
C	0.9362858	-1.0811945	0.2693338
H	0.1414493	-1.0723885	2.2498853
N	1.5366560	-0.8144078	-2.1118850
H	-2.4406627	-0.2540369	-2.6525531
H	-4.1171740	0.3083807	-2.8477192
H	-3.3797283	0.2824637	-1.2457687
H	-3.1452457	5.1598141	-3.1681921
H	-3.9050404	5.3470729	-1.5807957
H	-4.2535918	3.9118826	-2.5469906

H	-2.0671115	5.1298356	0.1205797
H	-1.2707567	5.6962668	-1.3585335
H	-0.6241582	4.2942588	-0.4911796
N	-2.3086912	2.9080435	2.7614384
N	-3.8116821	1.2793714	2.0171706
N	1.8016110	-2.0960465	0.3483276
C	2.5529888	-1.6959322	-2.2243594
C	-0.9502241	3.4422519	2.7665413
C	-3.2374334	3.5779129	3.6741687
C	-4.1068399	-0.1401579	1.8493459
C	-4.8926298	2.1874273	1.6293289
C	2.2761010	-2.6073507	1.4931380
N	2.3161626	-2.9033691	-2.7631877
N	3.8146318	-1.2735932	-2.0123509
H	-1.0082759	4.5299405	2.7881815
H	-0.3968965	3.1033918	3.6472456
H	-0.4203635	3.1444307	1.8683249
H	-4.0442590	2.9093933	3.9573900
H	-2.6882374	3.8516203	4.5766715
H	-3.6543444	4.4853501	3.2336992
H	-3.3999353	-0.7412412	2.4144891
H	-5.1125862	-0.3253653	2.2264572
H	-4.0735954	-0.4425247	0.7992318
H	-5.3476882	1.8117127	0.7118208
H	-5.6618894	2.2480527	2.4016982
H	-4.5000846	3.1796767	1.4319142
N	2.7461554	-1.8396295	2.5063026
N	2.3636138	-3.9504375	1.6036369
C	0.9585411	-3.4394043	-2.7726273
C	3.2474187	-3.5690038	-3.6764578
C	4.1073963	0.1460545	-1.8413690
C	4.8965047	-2.1807543	-1.6249690
C	2.7474890	-2.2704022	3.9029505
C	3.1852453	-0.4671516	2.3013512
C	3.4741877	-4.6282697	2.2731093
C	1.5313407	-4.8127986	0.7738587
H	1.0180435	-4.5269337	-2.7976709
H	0.4062152	-3.0982384	-3.6530446
H	0.4268517	-3.1453488	-1.8742472
H	4.0536868	-2.8984043	-3.9562991
H	2.7001509	-3.8407548	-4.5807201
H	3.6649698	-4.4771421	-3.2380587
H	3.4000173	0.7472114	-2.4058449
H	5.1132088	0.3336325	-2.2171421
H	4.0727644	0.4461549	-0.7906551
H	5.3497485	-1.8062600	-0.7060881
H	5.6668996	-2.2385992	-2.3964273
H	4.5052156	-3.1740223	-1.4301893
H	3.7611969	-2.4410296	4.2693020
H	2.2908702	-1.4794892	4.5025963
H	2.1575738	-3.1739034	4.0233271
H	2.4382681	0.2477323	2.6571108
H	4.1145821	-0.3163204	2.8505533
H	3.3766604	-0.2864315	1.2490074
H	3.1411357	-5.1672519	3.1625097
H	3.9013307	-5.3516596	1.5749990
H	4.2496901	-3.9182595	2.5438701
H	2.0633988	-5.1291367	-0.1279458
H	1.2692812	-5.7014858	1.3501053
H	0.6198519	-4.2977032	0.4881226
H	-3.7661984	2.4310375	-4.2699920

H	-2.2955185	1.4697621	-4.5020729
H	-2.1628105	3.1651115	-4.0260686
F	6.9976336	-3.3027595	1.1959858
B	6.9986589	-2.1798926	2.0423099
F	6.5314523	-1.0547100	1.3378719
F	8.3135486	-1.9345463	2.4929331
F	6.1667641	-2.4240891	3.1531158

(**1**+2H)$(BF_4)_2$, $\varepsilon_r = 40$

Illustration of the structure (C-H atoms omitted)

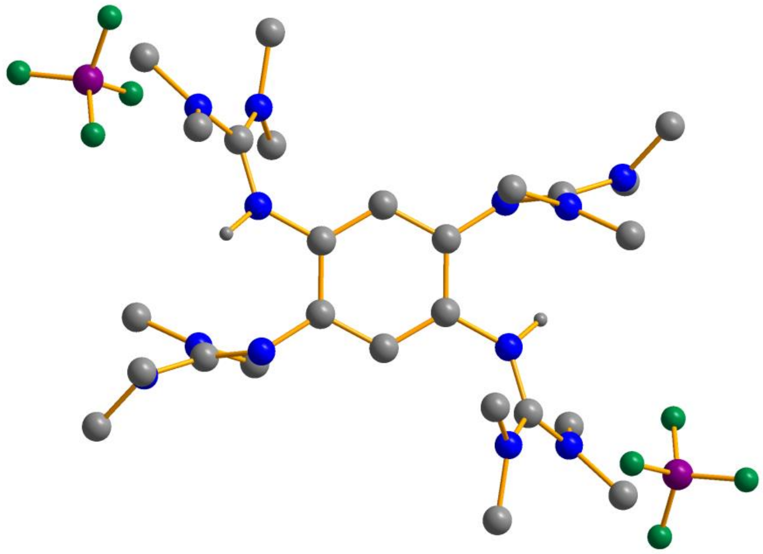

Energy = -2527.683935834 hartree

<u>Coordinates in Å</u>

F	-6.9670444	1.1527320	-2.3744461
B	-6.7721555	2.5443371	-2.2834910
F	-8.0122290	3.1784787	-2.0698484
F	-6.2089628	3.0160963	-3.4885735
F	-5.9053152	2.8332576	-1.2160511
N	-1.5018004	2.3014388	-0.5562634
C	-0.7870580	1.1044318	-0.2950355
C	-2.1144663	2.7179218	-1.6880412
C	-0.5658065	0.7751906	1.0593218
C	-0.2361940	0.3375095	-1.3147093
N	-2.7431039	1.8511617	-2.4928930
N	-2.0884514	4.0307380	-1.9671440
N	-0.9703511	1.6061998	2.1064642
C	0.2295043	-0.3438139	1.3151384
C	0.5590132	-0.7814332	-1.0587779
H	-0.3984500	0.6118479	-2.3480341
C	-2.8320758	2.0475824	-3.9405795
C	-3.3678424	0.6287032	-1.9948766
C	-3.2039019	4.7159236	-2.6250844
C	-1.0251474	4.9060843	-1.4744526
C	-2.2068768	1.8284666	2.4578748
C	0.7804502	-1.1105244	0.2954523
H	0.3921151	-0.6182635	2.3484219

N	0.9636328	-1.6129855	-2.1054209
H	-2.8063650	-0.2538265	-2.3077804
H	-4.3794926	0.5715705	-2.3961564
H	-3.4264017	0.6527384	-0.9123432
H	-2.9241824	5.0520966	-3.6248608
H	-3.4641061	5.5895933	-2.0250786
H	-4.0705763	4.0652212	-2.6811438
H	-1.3425633	5.4566908	-0.5846456
H	-0.7858327	5.6287675	-2.2546960
H	-0.1315266	4.3304282	-1.2499625
N	-2.4828714	2.9606128	3.1899456
N	-3.2837134	1.0236713	2.1651749
N	1.4956309	-2.3072501	0.5565575
C	2.1997886	-1.8338155	-2.4582162
C	-1.4829190	4.0131381	3.2767724
C	-3.3852962	2.9152504	4.3388581
C	-3.1577416	-0.4232289	2.0811549
C	-4.6213086	1.5484004	1.9177672
C	2.1164253	-2.7203216	1.6849963
N	2.4766191	-2.9667672	-3.1893225
N	3.2759808	-1.0272050	-2.1679288
H	-1.9805713	4.9395636	3.5672292
H	-0.7077541	3.7862302	4.0177256
H	-0.9954656	4.1607322	2.3161630
H	-3.9313710	1.9779706	4.3636959
H	-2.8088373	2.9968090	5.2666348
H	-4.0995023	3.7409781	4.3050605
H	-2.2432632	-0.7513298	2.5661552
H	-4.0039748	-0.8794841	2.6015858
H	-3.1585059	-0.7896395	1.0498426
H	-4.9915113	1.1744553	0.9598440
H	-5.3278888	1.2421929	2.6956582
H	-4.5979067	2.6332553	1.8698868
N	2.7478393	-1.8512780	2.4850843
N	2.0956607	-4.0330924	1.9657226
C	1.4773321	-4.0202852	-3.2732627
C	3.3734880	-2.9190627	-4.3426779
C	3.1483984	0.4197988	-2.0875514
C	4.6151260	-1.5494122	-1.9236820
C	2.8482668	-2.0479833	3.9320375
C	3.3641331	-0.6260324	1.9830547
C	3.2183855	-4.7152033	2.6143645
C	1.0305938	-4.9112973	1.4821066
H	1.9748573	-4.9458371	-3.5666930
H	0.6988753	-3.7934954	-4.0107857
H	0.9940603	-4.1695008	-2.3107800
H	3.9213055	-1.9827940	-4.3669840
H	2.7925493	-2.9965674	-5.2680513
H	4.0862598	-3.7462018	-4.3148130
H	2.2320246	0.7453064	-2.5706612
H	3.9924427	0.8757335	-2.6118310
H	3.1517940	0.7891116	-1.0573062
H	4.9873870	-1.1742182	-0.9669859
H	5.3191340	-1.2424658	-2.7036168
H	4.5937801	-2.6342963	-1.8752686
H	3.8682706	-2.2984010	4.2259869
H	2.5609399	-1.1154258	4.4209768
H	2.1685835	-2.8311807	4.2541433
H	2.8007392	0.2539146	2.2997733
H	4.3783362	-0.5647930	2.3771899
H	3.4151311	-0.6496256	0.9001378

H	2.9481528	-5.0519856	3.6165542
H	3.4759326	-5.5882821	2.0123140
H	4.0839336	-4.0624362	2.6634950
H	1.3421966	-5.4622567	0.5904405
H	0.7988695	-5.6337077	2.2649190
H	0.1341111	-4.3377135	1.2638813
H	-3.8498418	2.2979092	-4.2420766
H	-2.5410685	1.1149059	-4.4272306
H	-2.1497609	2.8305382	-4.2575877
F	5.9326384	-2.7897884	1.2313689
B	6.7832191	-2.5180532	2.3163237
F	6.9244519	-1.1256102	2.4723180
F	8.0474541	-3.0939671	2.0811303
F	6.2331331	-3.0660351	3.4947235
H	1.4083862	-2.9920189	-0.1817155
H	-1.4179803	2.9845063	0.1839899

(**1**+2H)$(BF_4)_4$, ε_r = 40

Illustration of the structure (C-H atoms omitted)

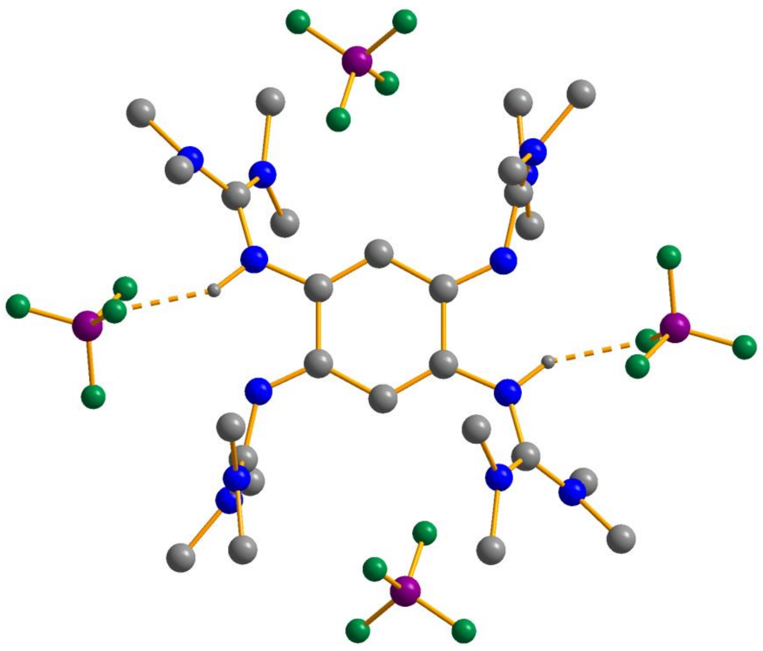

Energy = -3376.710257680 hartree

<u>Coordinates in Å</u>

F	6.6178211	1.8005450	4.3483027
B	6.0752939	3.0921186	4.2791082
F	4.7630539	3.0754275	4.8263941
F	6.0168009	3.5151666	2.9435209
F	6.8621969	3.9846875	5.0319964
C	2.9168296	8.9503084	6.6272396
C	3.6119460	7.7451546	6.9906743
C	2.4919786	8.0592738	4.3618314
C	3.7561115	6.7302317	6.1045382
C	3.1847350	6.8534155	4.7261383
N	3.3999233	5.8469664	3.9549918

F	2.0178813	8.4967075	1.1031483
B	3.2505898	6.2638814	10.7086576
B	2.8627329	9.5360012	0.6475099
F	2.4397173	6.7310422	11.7558660
F	2.4471224	5.8209825	9.6425260
F	4.0552655	5.2030976	11.1674122
F	3.6631051	9.0616013	-0.4044406
F	3.6761235	9.9652101	1.7116671
F	2.0695755	10.6081904	0.1954721
N	2.6986226	9.9554196	7.3985005
C	2.3497806	9.0752826	5.2472243
N	4.3843396	5.5363139	6.3493080
C	2.9755530	5.6862696	2.6540272
F	4.0839811	7.3123020	10.2533843
C	3.1146489	10.1169737	8.7017672
N	1.7281178	10.2715852	4.9997336
H	4.2691918	4.8122674	5.6363525
C	5.2268072	5.2042725	7.3955828
N	3.8460875	5.9210104	1.6806560
N	1.7546741	5.1766619	2.4571252
N	2.2417683	9.8725197	9.6706106
N	4.3297137	10.6374766	8.9058251
H	1.8451514	10.9960338	5.7119733
C	0.8871691	10.6061011	3.9530364
N	5.0404518	4.0206476	7.9646676
N	6.2091318	6.0365286	7.7423536
C	5.0515834	6.7208422	1.9223531
C	3.8438969	5.2062965	0.4000266
C	0.9983136	5.3370493	1.2106115
C	1.0159234	4.5396176	3.5516985
C	1.0436535	9.0642696	9.4214711
C	2.2304607	10.5876786	10.9508606
C	5.0812223	10.4799512	10.1556521
C	5.0694546	11.2813397	7.8158637
N	1.0799299	11.7874873	3.3815122
N	-0.1004743	9.7790584	3.6087627
C	3.7441882	3.3347598	7.9371238
C	6.1335378	3.2384671	8.5468365
C	6.7769544	6.0861757	9.0933012
C	6.8180713	6.9600886	6.7847666
C	2.3804624	12.4655064	3.4059665
C	-0.0088057	12.5744723	2.7976082
C	-0.6695002	9.7299118	2.2582945
C	-0.7141566	8.8615642	4.5690275
F	1.3721999	12.7402459	6.5137258
F	-0.7196710	11.8038683	6.3668349
B	0.0737383	12.7132351	7.0926705
F	-0.4864783	13.9975068	7.0231209
F	0.1684945	12.3048931	8.4302094
H	3.9718830	7.6552106	8.0069448
H	2.1338544	8.1500011	3.3449840
H	0.2250030	9.7029372	9.0848793
H	0.7700357	8.5803550	10.3563809
H	1.2385076	8.2871835	8.6908360
H	2.5421186	9.9382898	11.7690246
H	1.2070428	10.9156973	11.1312414
H	2.8658763	11.4652841	10.9078170
H	5.1226140	11.4122980	10.7193942
H	6.0988210	10.1918910	9.8899989
H	4.6508126	9.6887423	10.7591939
H	5.7533760	10.5815446	7.3303968

H	5.6522562	12.0966799	8.2415752
H	4.3836960	11.6910021	7.0804063
H	2.9441732	4.0432871	7.7535099
H	3.5852169	2.8802004	8.9139659
H	3.7471717	2.5567883	7.1729654
H	7.0928546	3.6291032	8.2249619
H	6.0345658	2.2159537	8.1828134
H	6.0772371	3.2295665	9.6354874
H	6.1213095	5.5871805	9.7980212
H	6.8537162	7.1345864	9.3810324
H	7.7735811	5.6445845	9.1166164
H	6.5721497	6.6730012	5.7677694
H	7.8994782	6.9087301	6.9075849
H	6.4978378	7.9871867	6.9707611
H	3.1764065	11.7522710	3.5890515
H	2.5403074	12.9177032	2.4281877
H	2.3836005	13.2443786	4.1691564
H	-0.9703481	12.1906838	3.1212471
H	0.0967060	13.5975288	3.1582515
H	0.0466130	12.5794930	1.7088946
H	-0.0109952	10.2228186	1.5519806
H	-0.7533418	8.6813191	1.9731789
H	-1.6632321	10.1779354	2.2344945
H	-0.4659114	9.1494501	5.5851892
H	-1.7952718	8.9181953	4.4460601
H	-0.3994918	7.8321845	4.3862073
H	5.8670148	6.0769328	2.2566608
H	5.3242596	7.2007586	0.9851262
H	4.8654675	7.5017378	2.6513352
H	3.5337798	5.8589003	-0.4161397
H	4.8634147	4.8694360	0.2137110
H	3.2009302	4.3343781	0.4462602
H	0.9468425	4.4037875	0.6492632
H	-0.0159436	5.6332039	1.4803973
H	1.4315948	6.1236647	0.6031573
H	0.3422335	5.2455683	4.0427136
H	0.4222763	3.7302023	3.1298381
H	1.7022956	4.1232422	4.2827834

(**1**+4H)(BF_4)$_4$, ε_r = 40

Illustration of the structure (C-H atoms omitted)

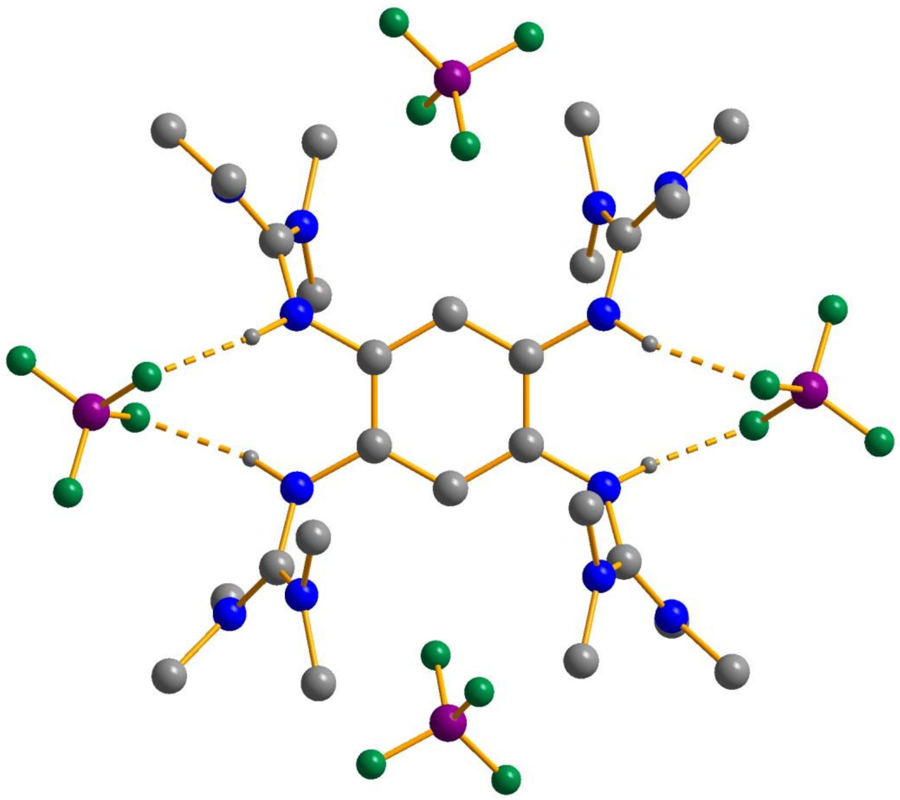

Energy = -3377.971978507 hartree

<u>Coordinates in Å</u>

F	2.9777584	-6.0553513	-1.2447481
B	2.1670984	-5.0197401	-1.7022271
F	1.0773482	-4.8306968	-0.8037982
F	1.6683341	-5.2981309	-2.9754936
F	2.9135088	-3.8089665	-1.7407193
C	-0.3930010	0.8869907	1.0139167
C	0.3199075	-0.2673576	1.3239892
C	-0.3151838	0.2703823	-1.3255063
C	0.7186936	-1.1513318	0.3269471
C	0.3976377	-0.8839423	-1.0154422
N	0.8869570	-1.7486458	-2.0168441
F	-0.9151328	1.4720656	-4.3441810
B	-0.2566252	-1.7270579	5.1034997
B	0.2455034	1.7254696	-5.1116322
F	-0.4312090	-0.6881830	6.0392395
F	-1.3662746	-1.7803026	4.2417749
F	-0.1172933	-2.9506346	5.7789482
F	0.4087524	0.6902787	-6.0534461
F	1.3612939	1.7695353	-4.2573946
F	0.1072806	2.9529351	-5.7803273
N	-0.8827570	1.7515952	2.0150134
C	-0.7143745	1.1541631	-0.3284796
N	1.3784355	-2.3616595	0.6471284
C	0.3622989	-1.9896141	-3.2536305
F	0.9081734	-1.4718453	4.3429573
C	-0.3611215	1.9904145	3.2535969
N	-1.3753589	2.3639452	-0.6488598

H	1.0590962	-3.2072155	0.1796028
C	2.3305126	-2.5574969	1.6007779
N	1.2130081	-2.1082943	-4.2723820
N	-0.9580794	-2.1492109	-3.4072084
N	-1.2146698	2.1088878	4.2699313
N	0.9589653	2.1484331	3.4110072
H	-1.0536959	3.2107119	-0.1849984
C	-2.3307604	2.5571421	-1.5996842
N	2.2847161	-3.6912635	2.3022622
N	3.3067253	-1.6568408	1.7739596
C	2.5325546	-1.4713290	-4.2647398
C	0.9611661	-2.9883265	-5.4144017
C	-1.6591011	-1.7908342	-4.6420213
C	-1.7965501	-2.6964886	-2.3421303
C	-2.5345006	1.4725767	4.2579335
C	-0.9652314	2.9871387	5.4138074
C	1.6561510	1.7879774	4.6473916
C	1.8007836	2.6962904	2.3488477
N	-2.2883795	3.6895139	-2.3036072
N	-3.3067542	1.6552207	-1.7678751
C	1.0300991	-4.4068570	2.5497233
C	3.4869913	-4.3630567	2.7972804
C	3.9416518	-1.4234341	3.0723663
C	3.7942597	-0.8244289	0.6747335
C	-1.0353859	4.4060047	-2.5565696
C	-3.4929467	4.3587740	-2.7964307
C	-3.9452186	1.4181622	-3.0638848
C	-3.7902857	0.8248312	-0.6653346
F	-1.0632318	4.8335486	0.7967555
F	-2.9003410	3.8203197	1.7411550
B	-2.1490557	5.0279923	1.6987111
F	-2.9571287	6.0663687	1.2429382
F	-1.6447366	5.3056207	2.9699553
H	0.5062853	-0.5257655	2.3581919
H	-0.5018763	0.5286594	-2.3597034
H	-2.5389261	0.6222930	3.5830888
H	-3.3061879	2.1885590	3.9689838
H	-2.7426059	1.1119908	5.2641206
H	-0.7547160	2.4088569	6.3141883
H	-1.8629436	3.5827555	5.5792282
H	-0.1408010	3.6599321	5.2028541
H	1.9646895	2.6752438	5.2019222
H	2.5489824	1.2231180	4.3788668
H	1.0250381	1.1562554	5.2640975
H	2.3814907	1.9164465	1.8530693
H	2.4925532	3.4095250	2.7967897
H	1.1945749	3.2181270	1.6144455
H	0.1815811	-3.7490441	2.3898379
H	1.0239091	-4.7319274	3.5897167
H	0.9496308	-5.2804946	1.9010449
H	4.3733760	-3.9531420	2.3235570
H	3.4115940	-5.4196852	2.5387636
H	3.5739032	-4.2757035	3.8812413
H	3.3345771	-1.8473050	3.8654519
H	4.0102662	-0.3459884	3.2258040
H	4.9507328	-1.8362910	3.1048079
H	3.5632914	-1.2870008	-0.2803214
H	4.8773374	-0.7465165	0.7631624
H	3.3700834	0.1806283	0.7062074
H	-0.1855543	3.7496191	-2.3978018
H	-1.0327494	4.7287094	-3.5973047

H	-0.9538576	5.2811604	-1.9100657
H	-4.3772819	3.9495683	-2.3182812
H	-3.4175367	5.4162445	-2.5414308
H	-3.5837231	4.2679962	-3.8798020
H	-3.3404372	1.8400387	-3.8597928
H	-4.0138842	0.3402751	-3.2141758
H	-4.9545236	1.8306046	-3.0947225
H	-3.5568243	1.2899717	0.2878875
H	-4.8735781	0.7459156	-0.7502569
H	-3.3654941	-0.1800310	-0.6957037
H	2.5388111	-0.6209908	-3.5900298
H	3.3054413	-2.1869945	-3.9782430
H	2.7373179	-1.1107850	-5.2716446
H	0.7479993	-2.4114180	-6.3150385
H	1.8587779	-3.5836798	-5.5812632
H	0.1376594	-3.6613087	-5.2004613
H	-1.9679283	-2.6789845	-5.1949707
H	-2.5520056	-1.2270116	-4.3715669
H	-1.0306137	-1.1587672	-5.2610617
H	-2.3774552	-1.9167708	-1.8463769
H	-2.4882050	-3.4116790	-2.7871601
H	-1.1877885	-3.2160045	-1.6082195
H	1.7678669	-2.2202088	-1.8328874
H	-1.7615508	2.2262510	1.8288043

$C_6H_4O_2$ (BQ), ε_r = 40

Illustration of the structure

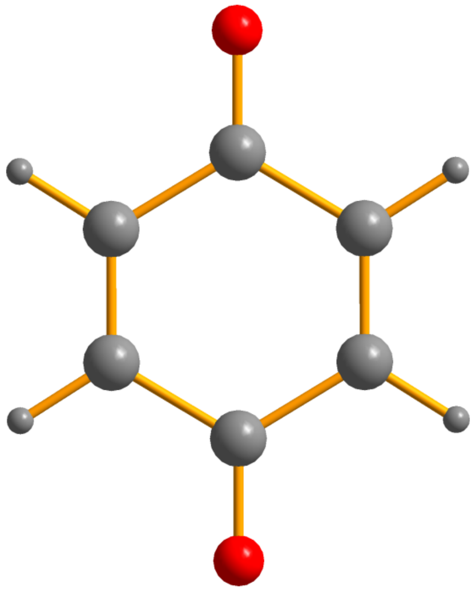

Energy = -381.4109504508 hartree

Coordinates in Å

C	1.4322862	-0.0000050	0.0000412
C	0.6682407	1.2669082	0.0000734
C	-0.6682460	1.2669415	0.0001413
C	-1.4322858	0.0000205	0.0000912
C	-0.6682328	-1.2669005	0.0000484

C	0.6682468	-1.2669168	0.0001177
O	2.6559619	-0.0000054	-0.0005880
H	1.2478738	2.1819144	0.0001103
H	-1.2478867	2.1819368	0.0001534
O	-2.6559420	-0.0000457	-0.0003672
H	-1.2478635	-2.1819070	0.0000491
H	1.2478476	-2.1819410	0.0001291

$C_6H_4(OH)_2$, $\varepsilon_r = 40$

Illustration of the structure

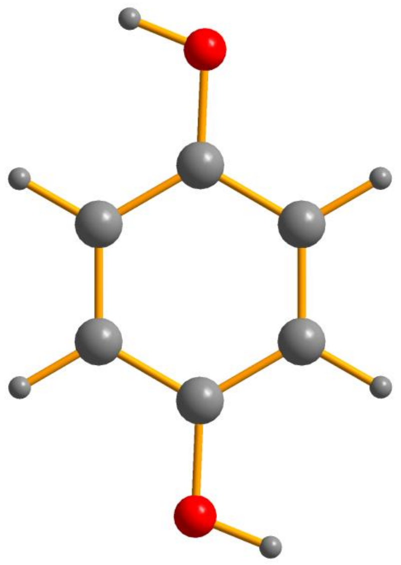

Energy = -382.6470112666 hartree

Coordinates in Å

C	1.3952972	-0.0287187	-0.0001132
C	0.7188433	1.1892124	-0.0000590
C	-0.6716969	1.2178767	-0.0001909
C	-1.3952999	0.0287490	-0.0002088
C	-0.7188434	-1.1891816	-0.0000904
C	0.6716871	-1.2178352	-0.0001401
O	2.7657244	-0.1118678	-0.0002734
H	1.2768110	2.1189175	0.0000200
H	-1.1990264	2.1636866	-0.0000489
O	-2.7657172	0.1118293	-0.0002580
H	-1.2767983	-2.1188934	0.0001121
H	1.1989293	-2.1636993	-0.0001258
H	3.1486472	0.7751728	0.0006409
H	-3.1485573	-0.7752483	0.0007357

($\mathbf{C_6H_4O_2}$+2H)$(BF_4)_2$, ε_r = 40

Illustration of the structure
(almost symmetric F⋯H⋯O bonds with F-H 1.360 Å, O-H 1.059 Å)

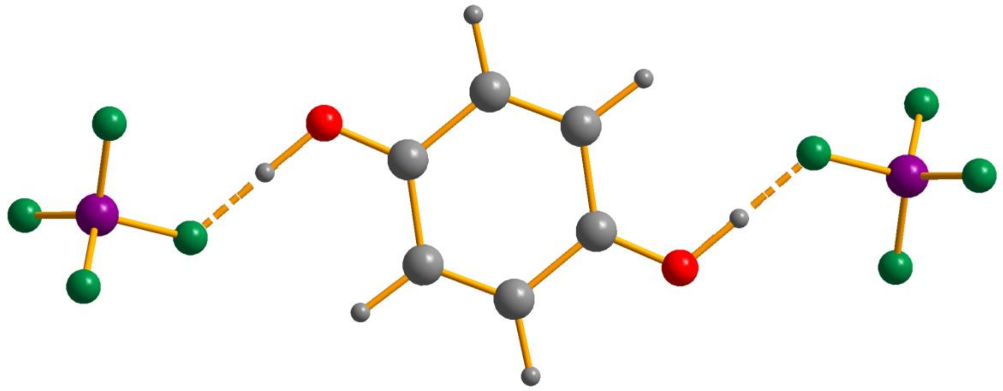

Energy = -1231.573823076 hartree

Coordinates in Å

C	1.3529137	-0.2923861	0.0135079
C	0.9267697	1.1053189	-0.0084731
C	-0.3801483	1.3838885	-0.0260458
O	2.5642423	-0.6155185	0.0328176
H	1.6812764	1.8805255	-0.0106560
H	-0.7638064	2.3947898	-0.0434775
H	-3.2670605	-0.1784509	-0.0408680
B	-5.4860508	-1.0866713	0.5908817
F	-4.1447597	-1.2171987	-0.0529536
F	-5.5319656	0.1882712	1.1367553
F	-5.5622051	-2.0712791	1.5584761
F	-6.4273490	-1.2613427	-0.4064217
C	-1.3544220	0.2916425	-0.0236340
C	-0.9282725	-1.1061400	-0.0029225
C	0.3786461	-1.3847217	0.0142687
O	-2.5657934	0.6149600	-0.0401522
H	-1.6826870	-1.8814713	-0.0008676
H	0.7622450	-2.3956728	0.0310449
H	3.2650769	0.1783105	0.0373919
B	5.4884010	1.0877479	-0.5769266
F	4.1424207	1.2172752	0.0573014
F	5.5386536	-0.1867746	-1.1234538
F	5.5716540	2.0730855	-1.5431342
F	6.4222211	1.2618122	0.4275413

$C_6Cl_4O_2$ (CA), ε_r = 40

Illustration of the structure

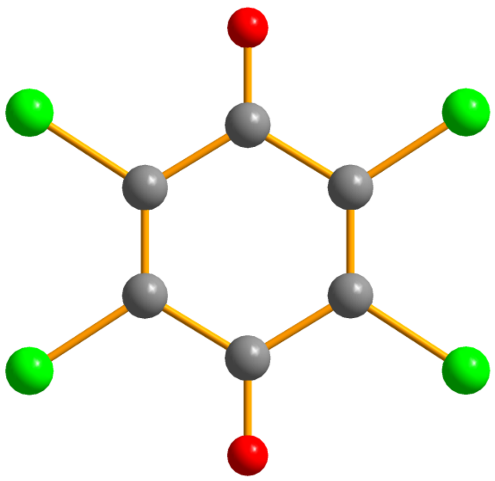

Energy = -2219.642096420 hartree

Coordinates in Å

C	1.4482053	-0.0000044	0.0000030
C	0.6716502	1.2755345	-0.0000673
C	-0.6716576	1.2755290	-0.0000142
C	-1.4481820	0.0000266	-0.0000486
C	-0.6716472	-1.2755393	-0.0000424
C	0.6716528	-1.2755368	-0.0000795
O	2.6569488	-0.0000022	0.0001418
Cl	1.6046409	2.7092145	-0.0001082
Cl	-1.6046665	2.7092140	0.0002064
O	-2.6569238	0.0000051	-0.0001015
Cl	-1.6046616	-2.7092007	0.0001033
Cl	1.6046407	-2.7092402	0.0000073

$C_6Cl_4(OH)_2$, ε_r = 40

Illustration of the structure

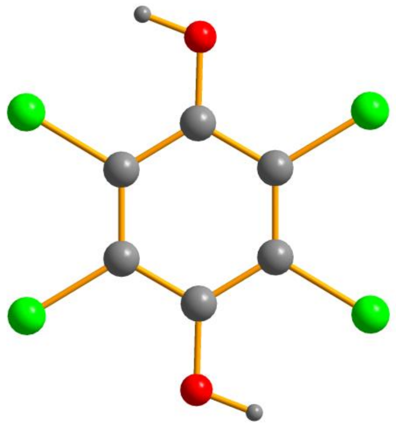

Energy = -2220.879308487 hartree

<u>Coordinates in Å</u>

C	1.4108543	-0.0313106	-0.0002219
C	0.7174583	1.1826838	-0.0001960
C	-0.6754763	1.2188624	-0.0002130
C	-1.4108527	0.0313106	-0.0001977
C	-0.7174556	-1.1826848	-0.0002535
C	0.6754729	-1.2188617	-0.0002679
O	2.7588524	-0.1051107	-0.0002705
Cl	1.6534966	2.6441532	-0.0001586
Cl	-1.5308001	2.7226666	-0.0003411
O	-2.7588577	0.1051091	-0.0001668
Cl	-1.6534932	-2.6441494	-0.0001118
Cl	1.5308000	-2.7226643	-0.0002269
H	3.1373557	0.7883611	0.0013776
H	-3.1373545	-0.7883653	0.0012481

$C_6Cl_4O_2(HF)_2(BF_3)_2$, ε_r = 40

Illustration of the structure

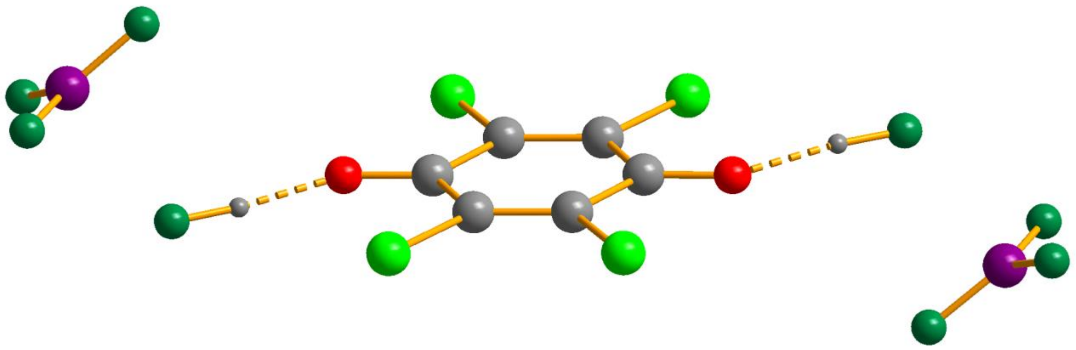

Energy = -3069.779806366

Coordinates in Å

C	-0.4401940	1.3106243	0.3375877
C	-1.3113594	0.1202829	0.5458477
C	-0.7690013	-1.2159650	0.1877978
Cl	-1.1155182	2.8143822	0.7678798
O	-2.4312636	0.2627582	0.9972159
Cl	-1.8041537	-2.5455805	0.4521066
H	-3.7464611	-0.6009326	1.3711316
B	-5.0873458	-0.9025372	3.8928683
F	-4.5819055	-0.9763258	1.6527029
F	-5.5989629	-2.1210371	3.8748702
F	-5.8847274	0.1441720	3.7643902
F	-3.8278536	-0.7247539	4.2568206
C	0.4729752	-1.3445629	-0.3137997
C	1.3429723	-0.1539004	-0.5252275
C	0.8019130	1.1819580	-0.1636419
Cl	1.1490480	-2.8486236	-0.7419794
O	2.4607042	-0.2955767	-0.9822104
Cl	1.8379811	2.5115100	-0.4248549
H	3.7700080	0.5722470	-1.3705294
B	5.0304402	0.9644620	-3.9248318
F	4.5997557	0.9517170	-1.6630303
F	5.5348236	2.1851705	-3.8788063
F	5.8378559	-0.0803099	-3.8608364
F	3.7602694	0.7908216	-4.2514713

$C_6Cl_2(CN)_2O_2$ (DDQ), ε_r = 40

Illustration of the structure

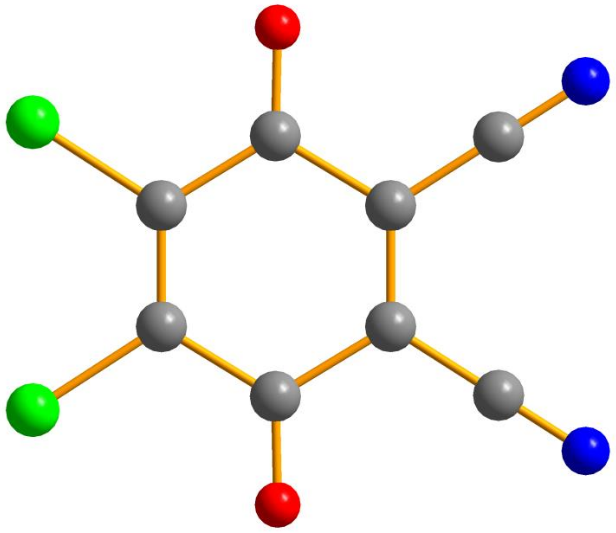

Energy = -1484.973474636

Coordinates in Å

C	1.4506101	0.0524352	-0.0000978
C	0.6743583	1.3203734	0.0000527
C	-0.6743944	1.3203524	-0.0000405
C	-1.4504768	0.0524349	-0.0001888
C	-0.6736507	-1.2299910	-0.0000356
C	0.6738629	-1.2300236	0.0000396
O	2.6582818	0.0273321	-0.0003546
Cl	1.6030087	2.7518292	0.0001247
Cl	-1.6031819	2.7517457	0.0000753
O	-2.6581552	0.0271383	-0.0004127
C	-1.4387910	-2.4307157	-0.0000960
C	1.4388319	-2.4309394	0.0001928
N	-2.0591861	-3.4001716	-0.0001948
N	2.0589633	-3.4005649	0.0004129

$C_6Cl_2(CN)_2(OH)_2$, ε_r = 40

Illustration of the structure

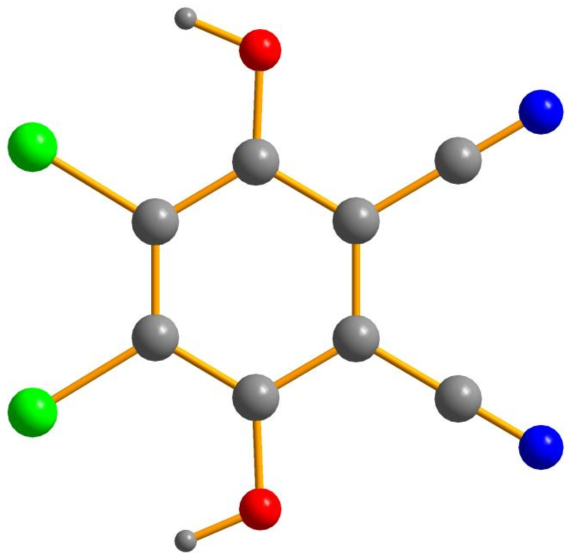

Energy = -1486.228543189

<u>Coordinates in Å</u>

C	1.4146333	0.0478979	-0.0002892
C	0.6945514	1.2539785	-0.0000347
C	-0.6945445	1.2539605	-0.0001085
C	-1.4145593	0.0478592	0.0002043
C	-0.7064138	-1.1550621	0.0002274
C	0.7065305	-1.1550638	-0.0002138
O	2.7543249	-0.0040769	-0.0007033
Cl	1.5928611	2.7270684	-0.0000252
Cl	-1.5929076	2.7270230	0.0002598
O	-2.7542670	-0.0042104	0.0006277
C	-1.4294776	-2.3801921	0.0003187
C	1.4295033	-2.3802829	-0.0002804
H	3.1353995	0.8889105	0.0010331
H	-3.1354172	0.8887405	-0.0011090
N	-2.0130494	-3.3742128	0.0004294
N	2.0128323	-3.3744493	-0.0003362

References

[1] TURBOMOLE V7.2 2017, a development of University of Karlsruhe and Forschungszentrum Karlsruhe GmbH, 1989-2007, TURBOMOLE GmbH, since 2007; available from http://www.turbomole.com.
[2] R. Ahlrichs, M. Bär, M. Häser, H. Horn, C. Kölmel, *Chem. Phys. Lett.* **1989**, *162*, 165–169.
[3] O. Treutler, R. Ahlrichs, *J. Chem. Phys.* **1995**, *102*, 346–354.
[4] A. D. Becke, *J. Chem. Phys.* **1993**, *98*, 5648–5652.
[5] P. J. Stephens, F. J. Devlin, C. F. Chabalowski, M. J. Frisch, *J. Phys. Chem.* **1994**, *98*, 11623–11627.
[6] F. Weigend, R. Ahlrichs, *Phys. Chem. Chem. Phys.* **2005**, *7*, 3297–3305.
[7] A. Klamt, G. Schürmann, *J. Chem. Soc. Perkin Trans. 2* **1993**, 799–805.

13) Acknowledgements

The authors gratefully acknowledge financial support by the *Deutsche Forschungsgemeinschaft* (DFG).
